# The Logic of Circadian Organization in *Drosophila*

**DOI:** 10.1016/j.cub.2014.08.023

**Published:** 2014-10-06

**Authors:** Stephane Dissel, Celia N. Hansen, Özge Özkaya, Matthew Hemsley, Charalambos P. Kyriacou, Ezio Rosato

**Affiliations:** 1 Department of Genetics, University of Leicester, Leicester LE1 7RH, UK

## Abstract

**Background:**

In the fruit fly *Drosophila melanogaster*, interlocked negative transcription/translation feedback loops provide the core of the circadian clock that generates rhythmic phenotypes. Although the current molecular model portrays the oscillator as cell autonomous, cross-talk among clock neurons is essential for robust cycling behavior. Nevertheless, the functional organization of the neuronal network remains obscure.

**Results:**

Here we show that shortening or lengthening of the circadian period of locomotor activity can be obtained either by targeting different groups of clock cells with the same genetic manipulation or by challenging the same group of cells with activators and repressors of neuronal excitability.

**Conclusions:**

Based on these observations we interpret circadian rhythmicity as an emerging property of the circadian network and we propose an initial model for its architectural design.

## Highlights

• Locomotor activity rhythms in *Drosophila* have 24 hr periodicity
• Different clock neurons promote either longer or shorter activity rhythms
• Circadian period is an emerging property of a network of diverse oscillators
• The logic connecting the network is beginning to emerge

## Graphical Abstract

**Figure:**
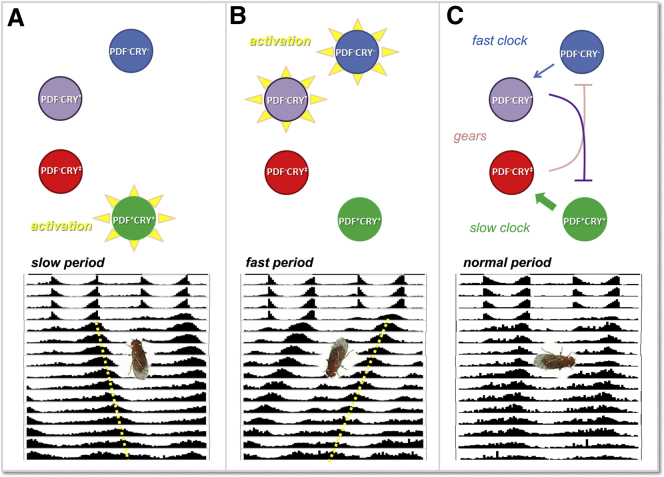

## Teaser

Our understanding of the circadian clock relies on the assumption that all rhythmic cells cycle with the same ∼24 hr rhythm that is characteristic of the organism as a whole. Dissel et al. show that the brain of *Drosophila* harbors a mosaic of cellular periodicities whose integration results in the ∼24 hr clock as we know it.

## Introduction

The circadian clock provides the interface between an organism and its 24 hr geophysical environment. As currently accepted, the fly clock is constituted by interlocked negative transcription/translation feedback loops (TTFLs). At the core of the system are the activators CLOCK (CLK) and CYCLE (CYC) that bind to the promoters of the *period* (*per*) and *timeless* (*tim*) genes, initiating their transcription. After translation, the negative autoregulators PER and TIM become the substrate of several kinases and phosphatases, they dimerize, translocate into the nucleus, and repress the CLK/CYC complex. The second feedback loop is centered around *Clk* and involves the rhythmic expression of *PAR domain protein 1* (*Pdp1*) and *vrille* (*vri*) (reviewed in [1]). The blue-light-sensitive protein CRYPTOCHROME (CRY) regulates photo-responsiveness and flies with altered CRY function display aberrant circadian light entrainment [2, 3, 4, 5] and visual behavior [6]. Moreover, immunofluorescence (IF) and confocal microscopy reveal that α-CRY immunoreactivity (IR) is often found at the level of neuronal projections [7], suggesting that CRY may play additional roles. Null mutants for *cry* (*cry*^*0*^) show defects in rhythmic behavior under constant light (LL) [8], so it is possible that CRY exerts direct functions in central clock neurons. Indeed, light-activated CRY increases neuronal firing via an unknown mechanism [9].

Clock neurons constitute a network of cells expressing clock genes that are divided into lateral neurons (six dorsal lateral neurons [LNds], four large ventral lateral neurons [l-LNvs], four small ventral lateral neurons [s-LNvs], a single *Pdf*-null ventral lateral neuron [pn-LNv], and three lateral posterior neurons [LPNs]) and dorsal neurons (∼16 DN1s, 2 DN2s, and ∼40 DN3s) (reviewed in [10]). Although the classic molecular model portrays the clock as cell autonomous, cellular cross-talk appears to be essential for its function [11, 12, 13, 14].

The circadian neurons can be further grouped or differentiated by the activation of promoters of clock and clock-related genes. For instance, all clock neurons express *tim* [15] whereas the *Pigment-dispersing factor* (*Pdf*) promoter is active only in the s- and l-LNvs [16]. A *cry* 5.5-kb promoter [17] is seemingly expressed in all LNvs, in the LNds, and in two DN1s [11] although Shafer et al. [18] reported further expression in two additional DN1s (called DN1a) and two DN3s. By combining the expression of these promoters, coupled to either *GAL4* (to drive transcription of a reporter gene) or *GAL80* (to inhibit GAL4 function), it is possible to define subsets of clock cells and to manipulate them selectively by expressing genes whose products alter the electrical properties of the neurons or the running of the clock.

In our study we have introduced local alterations in the neuronal network and have investigated the period of locomotor activity under constant darkness and temperature (DD), an artificial condition where the interactions among neurons is independent from the light-dark (LD) cycle. We did this by initially distinguishing among clock cells based on whether they express both the *Pdf* and *cry* promoters (PDF^+^CRY^+^), the *cry* promoter only (PDF^−^CRY^+^), or neither (PDF^−^CRY^−^). Our results suggest a model for the logic that regulates the neuronal network under these conditions.

## Results and Discussion

### CRYΔ Lengthens or Shortens the Endogenous Period of Locomotor Activity when Expressed in Different Clock Neurons

CRYΔ, a C-terminal deletion of CRY, renders CRY constitutively active, as revealed in a number of molecular (light-independent binding to PER and TIM) and behavioral (long period of locomotor activity in DD) phenotypes [4, 5]. Based on the discovery that light-activated CRY increases neuronal firing [9], we presumed that CRYΔ could activate neurons and enhance their output in DD, which might be reflected in period changes. We used a *tim-GAL4* driver and several *UAS-cryΔ* lines and confirmed that overexpression of CRYΔ in all clock cells, namely PDF^+^CRY^+^ ∩ PDF^−^CRY^+^ ∩ PDF^−^CRY^−^, results in ∼1 hr lengthening of the endogenous period of locomotor activity compared to controls [5] (Table S1 available online). We noticed that the vast majority of CRYΔ flies had simple rhythmicity (SR) although a few showed complex rhythms (CR, more than one periodicity in a single fly) or arrhythmicity (AR) but in proportions no different from controls.

We asked whether all clock cells contribute to the free running period or whether one group of neurons imposes its own rhythmicity to behavior [13]. We focused on one line (*UAS-cryΔ14.6*), which will hereafter be referred to as *cryΔ.*

**Figure 1:**
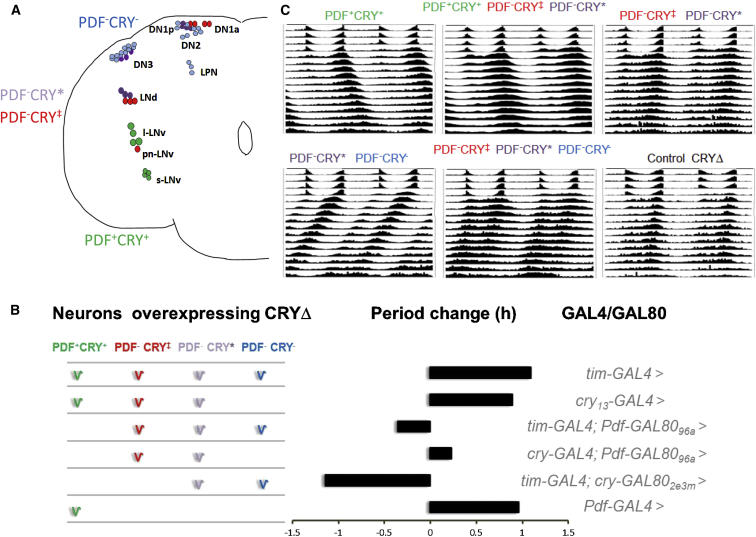
Different Groups of Neurons Affect Rhythmic Behavior (A) Operational classification of clock neurons according to the expression of the *Pdf* and *cry* promoters (see also text and Figure S1). (B) Period differences between CRYΔ-expressing and control flies. Horizontal black bars refer to the period change (in hours) compared to controls when CRYΔ (*UAS-cryΔ14.6*) is expressed in particular groups of neurons (shown on the left) as a result of different GAL4/GAL80 combinations (shown on the right). (C) Average locomotor activity profiles of CRYΔ-expressing flies showing 4 days in LD 12:12 and 12 days in DD. Genotypes and statistics are as reported in Table S1; control CRYΔ was *w*, *UAS-cryΔ14.6*.

PDF^+^CRY^+^ > CRYΔ (*Pdf-GAL4* > *UAS-cryΔ*) and PDF^+^CRY^+^ ∩ PDF^−^CRY^+^ > CRYΔ (*cry*_*13*_*-GAL4* > *UAS-cryΔ*) flies both revealed a ∼1 hr longer period compared to controls. We obtained a similar value for the PDF^+^CRY^+^ ∩ PDF^−^CRY^+^ ∩ PDF^−^CRY^−^ > CRYΔ genotype (*tim-GAL4* > *UAS-cryΔ*), which argues against the assumption that these three drivers largely differ in their “strength” [13] (Figure 1, Table S1). Then, we expressed CRYΔ in smaller groups of neurons by combining the GAL80 repressor with GAL4 drivers. We used the lines *cry-GAL80*_*2e3m*_ and *Pdf-GAL80*_*96a*_, each carrying two copies of GAL80, that have been reported to repress UAS-dependent expression in the PDF^+^CRY^+^ ∩ PDF^−^CRY^+^ and the PDF^+^CRY^+^ cells, respectively [11]. To confirm the extent of GAL80-mediated inhibition of GAL4 activity, we measured anti-GFP IR of PDF^+^CRY^+^ cells in *tim-GAL4* > *UAS-GFP*, in *tim-GAL4* ∩ *cry-GAL80*_*2e3m*_ > *UAS-GFP*, and in *tim-GAL4* ∩ *Pdf-GAL80*_*96a*_ > *UAS-GFP* flies. For both GAL80 configurations, we were able to confirm a good and equivalent level of repression of GAL4 activity in PDF^+^CRY^+^ cells (Figures S1A and S1B). However, in all *tim-GAL4* ∩ *cry-GAL80*_*2e3m*_ > *UAS-GFP* preparations, we observed a robust anti-GFP signal in three LNds, meaning that GAL80 cannot efficiently inhibit GAL4 in these cells because of lower *cry* expression (Figures S1A and S1C). GAL80-mediated repression of GAL4 was inefficient also in four DN1ps and in three or four *cry*-positive DN3s. Conversely, good repression was achieved in the DN1as, in two DN1ps, and in the pn-LNv (Figure S1C and data not shown). Thus, the PDF^−^CRY^+^ group is heterogeneous and should be divided into two. Henceforth we identify cells that strongly or weakly express *cry* as PDF^−^CRY‡ and PDF^−^CRY^∗^, respectively.

We tested PDF^−^CRY‡ ∩ PDF^−^CRY^∗^ > CRYΔ (*cry*_*13*_*-GAL4* ∩ *Pdf-GAL80*_*96a*_ > *UAS-cryΔ*) and PDF^−^CRY‡ ∩ PDF^−^CRY^∗^ ∩ PDF^−^CRY^−^ > CRYΔ (*tim-GAL4* ∩ *Pdf-GAL80*_*96a*_ > *UAS-cryΔ*) flies; for both genotypes the free running period of locomotor activity was not significantly different from controls. However, when we analyzed PDF^−^CRY^∗^ ∩ PDF^−^CRY^−^ > CRYΔ (*tim-GAL4* ∩ *cry-GAL80*_*2e3m*_ > *UAS-cryΔ*) flies, we observed a significant 1.2 hr shortening of the period (Figure 1; Table S1). This result cannot be explained in terms of residual GAL4 expression in the PDF^+^CRY^+^ cells as we detected similar levels of IR in them when driving GFP in both *tim-GAL4* ∩ *Pdf-GAL80*_*96a*_ and *tim-GAL4* ∩ *cry-GAL80*_*2e3m*_ flies (Figures S1A and S1B). CRYΔ overexpression resulted in a faster rhythm only for the latter genotype, thereby unveiling the contribution of the PDF^−^CRY^∗^ and PDF^−^CRY^−^ groups to period, which would otherwise be inhibited by the PDF^−^CRY‡ cells (this is because the two genotypes above differ in period and in the inclusion of the latter group of cells).

Interestingly, in wild-type flies the DN2 cluster of PDF^−^CRY^−^ neurons show faster (∼22 hr) endogenous molecular cycling that is not reflected in the ∼24 hr behavioral rhythms [19]. Under seminatural conditions, they show an advance in molecular cycling compared to other clock neurons, also consistent with a faster period [20, 21]. Conversely, it has been reported that by reducing the contribution of the PDF^+^CRY^+^ cells to the network, either by eliminating their main signaling molecule, the neuropeptide PDF [16] (Figure S2), or by reducing their numbers [11] (Table S2), a short activity period is generated. Thus, the overall evidence supports a role for the PDF^−^CRY^−^ neurons in generating a short-rhythm phenotype when the balance of the network is tilted in their favor, with the DN2s being a likely but perhaps not exclusive component of this function.

**Figure 2:**
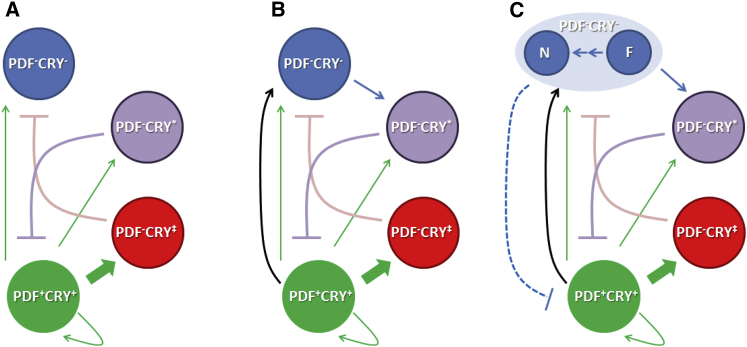
Circadian Logic We operationally divided the circadian neurons into PDF^+^CRY^+^, PDF^−^CRY‡, PDF^−^CRY^∗^, and PDF^−^CRY^−^ groups (see also Figures 1 and S1). (A) The PDF^+^CRY^+^ cells have a large influence on the network as they can communicate with a significant number of neurons through PDF (green arrows). Probably, the PDF^−^CRY‡ neurons are particularly responsive to PDF signaling (thick green arrow) as they express the PDF receptor (PDFR) at the highest level [37]. Activation of the PDF^+^CRY^+^ cells results first in a longer period of locomotor activity and then, as activation increases, in complex rhythms (Figure S3). Activation of the PDF^−^CRY^∗^ ∩ PDF^−^CRY^−^ cells results in a shorter period of locomotor activity and then in arrhythmicity. The PDF^−^CRY‡ and the PDF^−^CRY^∗^ cells have an inhibitory role toward the output of the PDF^+^CRY^+^ and the PDF^−^CRY^−^ neurons, respectively, thus balancing slower and faster components in the network. This could provide a simple mechanism to achieve phase changes under different environmental conditions. (B) The PDF^+^CRY^+^ also use PDF-independent connections (black arrow) to promote the activity of the PDF^−^CRY^−^ cells (see also Figure S2). The latter exert a negative feedback on the former through activation of the PDF^−^CRY^∗^ neurons. Thus, activation of PDF^+^CRY^+^ results in direct (PDF-independent) activation and in indirect (mediated and amplified by PDF and the PDF^−^CRY‡ neurons) repression of the PDF^−^CRY^−^ cells. These integrate both pathways and feedback to regulate the activity of the PDF^+^CRY^+^ neurons. This could explain how the PDF^+^CRY^+^ and PDF^−^CRY^−^ groups synchronize together. (C) Within the PDF^−^CRY^−^ group, the N neurons (including the majority of DN1s) are involved in stabilizing behavioral rhythms through synchronization with the PDF^+^CRY^+^, which suggests they might have an independent connection with those cells (broken blue line). The F neurons (which include the DN2s and perhaps unrecognized PDF^−^CRY^−^ neurons) have an intrinsically faster molecular rhythm. We assume that the signal to inhibit the PDF^+^CRY^+^ group can be passed on to the PDF^−^CRY^∗^ before the PDF^−^CRY‡ cells have time to exert their repression. This would explain how the fast PDF^−^CRY^−^ counteract the slower cycling of the PDF^+^CRY^+^ neurons, resulting in a 24 hr period. However, the signal for a faster rhythm would not usually reach the N neurons before the repression from the PDF^−^CRY‡ cells takes effect, suggesting a delay in the connection between the F and N groups of PDF^−^CRY^−^ neurons. The activation of the PDF^−^CRY^∗^ and PDF^−^CRY^−^ groups (or a reduction in the activation of the PDF^−^CRY‡ cells) would overcome that delay, causing the N cells (such as the DN1s) to cycle in synchrony with the F cells (such as the DN2s); see Figure 4. The N neurons would then pass on the shorter-period signal to the PDF^+^CRY^+^ group (such as the s-LNvs, Figure 4), causing a shorter behavioral period.

We can rationalize this first set of observations as follows. The overexpression of CRYΔ in PDF^+^CRY^+^ cells (*Pdf-GAL4* > *UAS-cryΔ*) results in a longer period. However, these slower-paced cells are not alone in controlling self-sustained behavior because increasing the impact on the network of the PDF^−^CRY^∗^ and PDF^−^CRY^−^ neurons (*tim-GAL4* ∩ *cry-GAL80*_*2e3m*_ > *UAS-cryΔ*) results in a shorter period. The effect is reversed by adding PDF^−^CRY‡ to the ensemble (*tim-GAL4* ∩ *Pdf-GAL80*_*96a*_ > *UAS-cryΔ*), suggesting the latter intervene in a negative control, possibly on the faster PDF^−^CRY^−^ neurons (Figure 2A). The simultaneous activation of PDF^−^CRY‡ and PDF^−^CRY^∗^ neurons (*cry*_*13*_*-GAL4* ∩ *Pdf-GAL80*_*96a*_ > *UAS-cryΔ*) had no consequence in terms of period (Figure 1; Table S1). This suggests a balance, with the PDF^−^CRY‡ neurons inhibiting the faster-paced PDF^−^CRY^−^ cells and in turn the PDF^−^CRY^∗^ neurons inhibiting the slower-paced PDF^+^CRY^+^ cells (Figure 2A). Note that in this context, the words “activation” and “inhibition” do not have a physiological connotation but are used as logical operators.

### Additional Manipulations Consolidate the Model

We wanted to show that the effects observed on period are not specific to CRYΔ but are reproducible by other manipulations. Thus, we employed the same GAL4/GAL80 combinations described above, using effectors that have a known and consistent mode of action independent of the type of neuron.

**Figure 3:**
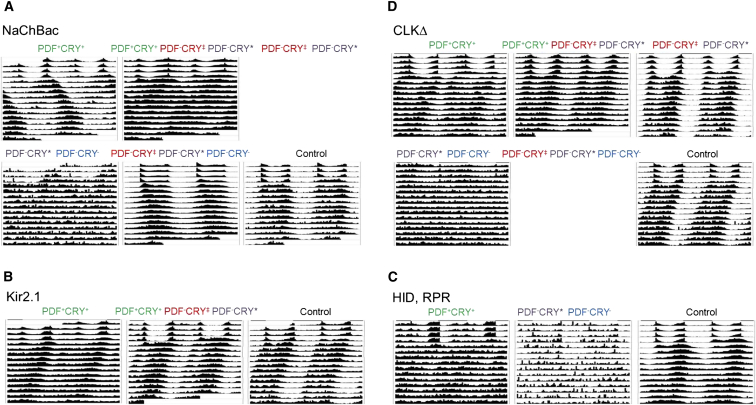
Locomotor Activity Profiles after Manipulation of Clock Neurons Average locomotor activity profiles of flies showing 3–4 days in LD 12:12 and 12 days in DD. Genotypes and statistics are reported in Table S2. (A) NaChBac overexpression, line *NaChBac4* is shown. Control: *w; +/ UAS-NaChBac4;+/+*. (B) Kir2.1 overexpression, line *Kir2.1(III)* is shown. Control: *w; +/+; UAS-Kir2.1/+*. (C) HID, RPR overexpression. Control: *w, UAS-hid,UAS-rpr; +/+;+/+*. (D) CLKΔ overexpression, line *CLKΔ1* is shown. Control: *w; UAS-ClkΔ1/+; +/+*.

The overexpression of the depolarization-activated bacterial sodium channel NaChBac [19] using *tim-GAL4* (PDF^+^CRY^+^ ∩ PDF^−^CRY‡ ∩ PDF^−^CRY^∗^ ∩ PDF^−^CRY^−^) resulted predominantly in arrhythmicity, confirming the effectiveness of this manipulation (Table S2). Limiting NaChBac expression to the PDF^+^CRY^+^ (*Pdf-GAL4>UAS-NaChBac*) neurons generated ∼50% of flies with complex rhythms, showing a major long-period and a minor short-period component. Moreover, those ∼30%–40% individuals with a single activity rhythm had a period 1.5–2.5 hr longer than controls (Figure 3A; Table S2). NaChBac overexpression in PDF^+^CRY^+^ induces longer molecular cycles in these cells and directly increases their output, explaining the origin of the major, slower (longer period) activity component [22, 23]. Immunocytochemistry experiments have also indicated that the DN2s show a faster cycle in both controls and NaChBac-overexpressing flies [19]. Thus, it is tempting to speculate that fast PDF^−^CRY^−^ neurons (we cannot exclude that neurons in addition to the DN2s might also be involved) might be indirectly activated through a positive, PDF-independent connection linking the PDF^+^CRY^+^ and the PDF^−^CRY^−^ cells and be responsible for the minor, short activity component detected in this genotype (Figures 2B and S2).

Only 13%–25% of PDF^−^CRY^∗^ ∩ PDF^−^CRY^−^ > NaChBac (*tim-GAL4* ∩ *cry-GAL80*_*2e3m*_ > *UAS-NaChBac*) flies showed single rhythms (Figure 3A; Table S2). This result supports the hypothesis that the PDF^−^CRY^∗^ cells might inhibit the PDF^+^CRY^+^ neurons, explaining why they do not sustain behavioral rhythms in this genotype (Figure 2B). Subsequently we tested PDF^−^CRY‡ ∩ PDF^−^CRY^∗^ ∩ PDF^−^CRY^−^ > NaChBac flies (*tim-GAL4* ∩ *2xPdf-GAL80* > *UAS-NaChBac*; Figure 3A; Table S2). Now, about 70%–80% of individuals had a single activity component with a period 0.5–1 hr longer than controls. This implies that the addition of the PDF^−^CRY‡ group (which is not included in the largely arrhythmic *tim-GAL4* ∩ *cry-GAL80*_*2e3m*_ > *UAS-NaChBac* genotype) to the ensemble of activated neurons results in the inhibition of the output of the PDF^−^CRY^−^ cells (because of the longer activity period) and of the PDF^−^CRY^∗^ cells (because the majority of flies are rhythmic). Possibly, the latter inhibition is indirect, in agreement with our previous hypothesis (Figure 2B).

The expression of NaChBac in PDF^+^CRY^+^ ∩ PDF^−^CRY‡ ∩ PDF^−^CRY^∗^ (*cry*_*13*_*-GAL4 > UAS-NaChBac*) neurons caused a decrease in the number of rhythmic individuals and a shortening in the activity period (now reaching wild-type values for single-rhythm flies) compared to the PDF^+^CRY^+^ > NaChBac (*PdfGAL4* > *UAS-NaChBac*) genotype (Figure 3A; Table S2). This result is different from the comparison between the same genotypes for CRYΔ flies, which were both rhythmic with a period about 1 hr longer than controls (Figure 1; Table S1). The different strength of the two activators likely accounts for these discrepancies, which in general result from exaggerated effects on period and on rhythmicity following NaChBac overexpression (Figures S3).

We also applied the opposite manipulation, namely the overexpression of the mammalian inward rectifier K^+^ channel KIR2.1, a tool for decreasing membrane excitability in vivo [24, 25]. PDF^+^CRY^+^ > KIR2.1 (*Pdf-GAL4 > UAS-kir2.1*) resulted in more than a third of the flies being arrhythmic and in the tendency for a shorter period for those showing a single rhythm. This argues for a disruption in the balance of the network and, in the flies still rhythmic, for a tilt toward the faster PDF^−^CRY^−^ neurons. Extending the misexpression of KIR2.1 to include also the PDF^−^CRY‡ and the PDF^−^CRY^∗^ cells (*cry*_*13*_*-GAL4 > UAS-kir2.1*) abolished these effects (Figure 3B; Table S2). Finally, we tested the expression of KIR2.1 in PDF^−^CRY^∗^ ∩ PDF^−^CRY^−^ neurons (*tim-GAL4* ∩ *cry-GAL80*_*2e3m*_ > *UAS-kir2.1*), but this genotype was not viable.

We then overexpressed the proapoptotic genes *head involution defective* (*hid*) and *reaper* (*rpr*) [26] in PDF^+^CRY^+^ cells (*Pdf-GAL4 > UAS-hid, UAS-rpr*) and observed that 50% of the flies were equally either arrhythmic or showed complex rhythms. The remaining half cycled with a period of locomotor activity ∼1 hr shorter than controls. Expression of HID and RPR in PDF^−^CRY^∗^ ∩ PDF^−^CRY^−^ cells (*tim-GAL4* ∩ *cry-GAL80*_*2e3m*_ > *UAS-hid, UAS-rpr*) resulted in poor viability and ∼60% of the surviving flies were arrhythmic, further underscoring the importance of these neurons to the network (Figure 3C; Table S2). The remaining flies showed complex or single rhythms, the latter having periods on average intermediate to their corresponding controls (Table S2). Although we did not verify this experimentally, it is likely that viable and rhythmic flies must have largely escaped the apoptotic response triggered by HID and RPR. This explanation is in line with the lethality of the *tim-GAL4* ∩ *cry-GAL80*_*2e3m*_ > *UAS-kir2.1* genotype and with previously reported variability in the induction of the apoptotic response in neurons [27].

Driving the production of the dominant-negative CLKΔ mutant [28] in PDF^−^CRY^∗^ ∩ PDF^−^CRY^−^ cells (*tim-GAL4* ∩ *cry-GAL80*_*2e3m*_ > *UAS-ClkΔ*) resulted in virtually complete arrhythmicity. Thus, as with NachBac and HID-RPR overexpression, disrupting (independently of mechanism) the PDF^−^CRY^∗^ ∩ PDF^−^CRY^−^ groups generated a largely arrhythmic profile, which argues against a simple dominant effect of the PDF^+^CRY^+^ cells on the neuronal network (Figure 3D; Table S2). The expression of CLKΔ in PDF^+^CRY^+^ cells caused 20%–50% arrhythmicity and 10%–30% of complex rhythms, depending on the line. The remaining 40%–50% of flies showed a single rhythm of activity with a period 0.5–1 hr shorter than controls. This is in line with an increased influence of the PDF^−^CRY^−^ cells on the network. By expressing CLKΔ in PDF^−^CRY‡ ∩ PDF^−^CRY^∗^ cells in addition to the PDF^+^CRY^+^ group (*cry*_*13*_*-GAL4 > UAS-ClkΔ*), we increased the ratio of single-rhythm flies to 72%–94% and reverted to a period closer to that of the parental control strains, but slightly shorter (Figure 3D; Table S2). Thus, as previously seen with the overexpression of KIR2.1, we could rescue rhythmicity in flies with weakened PDF^+^CRY^+^ neurons by reducing the inhibitory influence of the PDF^−^CRY^∗^ cells on them and by buffering changes in activation of the PDF^−^CRY‡ cells. Again, this shows that the PDF^+^CRY^+^ cells do not act alone in determining circadian rhythmicity and its period. We were unable to obtain viable PDF^−^CRY‡ ∩ PDF^−^CRY^∗^ ∩ PDF^−^CRY^−^ > CLKΔ (*tim-GAL4,* ∩ *Pdf-GAL80*_*96a*_ > *UAS-ClkΔ*) adults but PDF^−^CRY‡ ∩ PDF^−^CRY^∗^ > CLKΔ (*cry*_*13*_*-GAL4,* ∩ *Pdf-GAL80*_*96a*_ > *UAS-ClkΔ*) flies were rhythmic with periods indistinguishable from controls (Figure 3D; Table S2). This result is the same as that observed with CRYΔ, again suggesting that the PDF^−^CRY‡ and the PDF^−^CRY^∗^ cells inhibit the faster PDF^−^CRY^−^ and the slower PDF^+^CRY^+^ cycling groups respectively, so the balance of the network is undisturbed (Figure 2B).

### Expression of CRYΔ Sets off Nonautonomous Effects on Cellular Clocks

**Figure 4:**
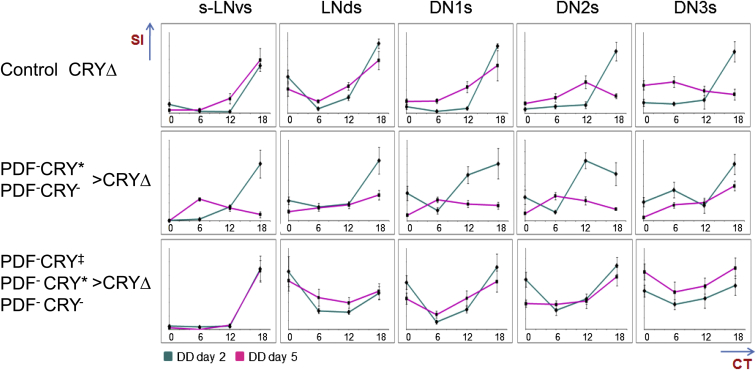
PDP1ε Immunoreactivity after Expression of CRYΔ in Different Groups of Neurons Staining index (SI, error bars correspond to the standard error of the mean) for PDP1ε at CT0, CT6, CT12, and CT18 during day 2 (green) and 5 (magenta) in DD. ANOVA showed a significant effect (p < 0.05) of time of day on SI values, with the following exceptions. Control CRYΔ, DN3s at day 5. PDF^−^CRY^∗^ ∩ PDF^−^CRY^−^ > CRYΔ, LNds at day 5 and DN3s at day 5. PDF^−^CRY‡ ∩ PDF^−^CRY^∗^ ∩ PDF^−^CRY^−^ > CRYΔ, LNds at day 5 and DN3s at day 2 and 5. For the two CRYΔ-expressing genotypes, we also tested the interaction among factors by comparing the SI of cell types s-LNvs, DN1s, and DN2s at different time points, at day 2 and 5. (PDF^−^CRY^∗^ ∩ PDF^−^CRY^−^ > CRYΔ, ANOVA, Day, F_1,215_ = 31.09, p < < 0.01; Time, F_3,215_ = 24.83, p < < 0.01; Cell type, F_2,215_ = 6.29, p < 0.01; Day^∗^Time, F_3,215_ = 18.26, p < < 0.01; Day^∗^Cell type, F_2,215_ = 0.88, p = 0.42; Time^∗^Cell type, F_6,215_ = 4.50, p < < 0.01; Day^∗^Time^∗^Cell type, F_6,215_ = 3.50, p < 0.01. PDF^−^CRY‡ ∩ PDF^−^CRY^∗^ ∩ PDF^−^CRY^−^ > CRYΔ, ANOVA, Day, F_1,193_ = 2.33, p = 0.13; Time, F_3,193_ = 50.73, p < < 0.01; Cell type, F_2,193_ = 36.67, p < < 0.01; Day^∗^Time, F_3,193_ = 2.71, p = 0.046; Day^∗^Cell type, F_2,193_ = 0.83, p = 0.44; Time^∗^Cell type, F_6,193_ = 3.06, p < 0.01; Day^∗^Time^∗^Cell type, F_6,193_ = 0.82, p = 0.56.) PDP1ε did not cycle in l-LNvs, so they are not shown. We did not measure the PDF-negative LNv and the lateral posterior neurons (LPNs) because we were unable to identify them unequivocally in all preparations. For each cluster of neurons, we calculated the average SI per hemisphere (each considered as an independent observation) and we used those values for statistical comparisons. The number of hemispheres analyzed are given below as GENOTYPE, DAY [cell type1 (time points), cell type2 (time points), etc.]. Cell types are s-LNvs, LNds, DN1s, DN2s, DN3s, respectively. Time point are CTO, CT6, CT12, CT18, respectively. Control CRYΔ, DD2 [(9, 10, 11, 8), (9, 8, 11, 7), (10, 9, 11, 9), (10, 6, 10, 8), (10, 10, 10, 6]; DD5 [(11, 9, 12, 14), (11, 9, 11, 14), (9, 10, 9, 11), (10, 5, 9, 10), (10, 9, 10, 12)]. PDF^−^CRY^∗^ ∩ PDF^−^CRY^−^ > CRYΔ, DD2 [(11, 9, 10, 9), (11, 9, 10, 10), (12, 7, 10, 10), (11, 8, 9, 10), (11, 8, 8, 10)]; DD5 [(11, 10, 12, 9), (11, 10, 13, 11), (10, 10, 10, 11), (10, 9, 10, 11), (9, 10, 10, 8)]. PDF^−^CRY‡ ∩ PDF^−^CRY^∗^ ∩ PDF^−^CRY^−^ > CRYΔ, DD2 [(9, 13, 10, 8), (9, 10, 10, 8), (8, 7, 10, 8), (8, 5, 10, 7), (8, 7, 9, 8)]; DD5 [(11, 10, 10, 11), (12, 11, 10, 11), (9, 9, 8, 11), (9, 6, 8, 12), (10, 9, 9, 9)]. Genotypes: Control CRYΔ, *w, UAS-cryΔ14.6; +/+; +/+*. PDF^−^CRY^∗^ ∩ PDF^−^CRY^−^ > CRYΔ, *w, UAS-cryΔ14.6; tim-GAL4/+; cry-GAL80*_*2e3m*_*/+*. PDF^−^CRY‡ ∩ PDF^−^CRY^∗^ ∩ PDF^−^CRY^−^ > CRYΔ, *w, UAS-cryΔ14.6; tim-GAL4/Pdf-GAL80*_*96a*_*; +/+*.

CRYΔ flies with a long behavioral period exhibit a delay in the cycling of clock proteins in PDF^+^CRY^+^ neurons [5]. Here we investigated the cellular changes caused by CRYΔ overexpression in PDF^−^CRY^∗^ ∩ PDF^−^CRY^−^ (*tim-GAL4* ∩ *cry-GAL80*_*2e3m*_ > *UAS-cryΔ*) and in PDF^−^CRY‡ ∩ PDF^−^CRY^∗^ ∩ PDF^−^CRY^−^ (*tim-GAL4* ∩ *Pdf-GAL80*_*96a*_ > *UAS-cryΔ*) neurons, resulting in short and normal (compensatory) activity rhythms, respectively. We analyzed the cycling of the clock protein PDP1ε in the major classes of clock neurons by IF. Figure 4 shows the staining index (SI) for PDP1 ε at CT0, CT6, CT12, and CT18 (circadian time, CT0 = subjective lights ON, CT12 = subjective lights OFF) during day 2 and 5 in DD.

Control flies (*UAS-cryΔ*) showed, at day 2, maximum SI at about CT18 for all cell types. At day 5 the SI peaked also at CT18 for s-LNvs, LNds, and DN1s, suggesting that these cells cycle with a period of about 24 hr. Instead the SI maximum was reached earlier (about CT12) by the DN2s, suggesting that these neurons run with a shorter period of ∼22 hr. These cells also showed a pronounced reduction in their cycling amplitude compared to the other clusters. The DN3 did not cycle at day 5. Thus, in control flies, s-LNvs, LNds, and DN1s showed molecular oscillations that were compatible with the corresponding behavioral period attributed to them.

The overexpression of CRYΔ in PDF^−^CRY^∗^ ∩ PDF^−^CRY^−^ cells (*tim-GAL4* ∩ *cry-GAL80*_*2e3m*_ > *UAS-cryΔ*) targeted directly the DN2s, the large majority of DN1s, the DN3s, and half of the LNds. The DN1s and even more so the DN2s showed a phase advance in PDP1ε immune staining at day 2; at day 5 both groups reached their maximum at around CT6. However, while the DN1s experienced a shortening of their period (in this genotype compared to the control), the DN2s maintained the same faster rhythm seen in wild-type flies. The DN3 did not show significant cycling at day 5. The LNds were considered as a group because we could not distinguish the three PDF^−^CRY‡ from the three PDF^−^CRY^∗^ cells (we could not discriminate them by anti-CRY staining because both the anti-CRY and the anti-PDP1ε antibodies available to us are made in rabbit), which limits our interpretation of these data. Overall, the LNds showed a reduction in cycling amplitude especially at day 5, perhaps resulting from averaging different cycling profiles in the two groups. The s-LNvs experienced phase advance and shortening of their rhythm such that by day 5 they reached the SI maximum at CT6 (although because of the 6 hr resolution of our data, the actual peak might be a few hours later). Thus, in this genotype, the s-LNvs, the DN1s, and the DN2s were synchronous and showed endogenous cycling compatible with the short behavioral period. A significant “time × cell type” ANOVA interaction (we compared the SI of cell types s-LNvs, DN1s, and DN2s, at different time points, at day 2 and 5; see legend to Figure 4) provides support to the view that the advance in the phase of PDP1ε expression occurred first in the DN2s, then in the DN1s, and finally in the s-LNvs. The changes above appeared first at day 2 and were consolidated at day 5, confirmed with a significant “day × time × cell type” ANOVA interaction (Figure 4).

We then overexpressed CRYΔ in PDF^−^CRY‡ ∩ PDF^−^CRY^∗^ ∩ PDF^−^CRY^−^ cells (*tim-GAL4* ∩ *Pdf-GAL80*_*96a*_ > *UAS-cryΔ*), namely in the DNs and in both types of LNds (the pn-LNv was also affected but it has not been analyzed here). Extending the expression of CRYΔ to the PDF^−^CRY‡ cells caused a broadening in the expression profile of PDP1ε in the DNs, supporting our hypothesis that the PDF^−^CRY‡ modulate the PDF^−^CRY^−^ cells. Especially at day 2, the levels of PDP1ε started to rise earlier (compared to controls) than for PDF^−^CRY^∗^ ∩ PDF^−^CRY^−^ > CRYΔ flies, but declined later in all dorsal neurons, suggesting a combination of phase advance and phase delay inputs. The DN2s maintained their shorter molecular cycling while the DN1s and seemingly the DN3s moved to a ∼24 hr period. The LNds, also expressing CRYΔ directly, showed the most pronounced phase delay (supported by a significant “time × cell type” ANOVA interaction) and a reduction in cycling amplitude (although smaller than for the previous genotype). Those differences were maintained at day 5, in agreement with a nonsignificant “day × time × cell type” ANOVA interaction (see legend to Figure 4). The profile of PDP1ε IR in the s-LNvs (not expressing CRYΔ) remained unchanged at day 2 and 5, which, compared to the controls, corresponds to a small delay in phase but no change in period. In this genotype, only the sLNvs and the DN1s showed robust endogenous cycling compatible with the behavioral period.

Overall, only the s-LNvs and the DN1s consistently showed cellular cycling matching the behavioral periods of the flies in all genotypes. We suggest that locomotor activity with simple rhythmicity requires synchronization between these two groups of neurons, although the contribution of neurons from other clusters may also be significant. In the PDF^−^CRY^∗^ ∩ PDF^−^CRY^−^ > CRYΔ (*tim-GAL4* ∩ *cry-GAL80*_*2e3m*_ > *UAS-cryΔ*) flies, the faster rhythm of the DN2s and perhaps of additional unidentified fast PDF^−^CRY^−^ cells was shared by DN1s and s-LNvs. In PDF^−^CRY‡ ∩ PDF^−^CRY^∗^ ∩ PDF^−^CRY^−^ > CRYΔ (*tim-GAL4* ∩ *Pdf-GAL80*_*96a*_ > *UAS-cryΔ*) individuals, the DN2s were still cycling with a faster pace but, as in wild-type flies, the shorter period did not spread to DN1s and s-LNvs, suggesting this is the role of the highly PDF-responsive PDF^−^CRY‡ cells (included in the second but not in the first genotype). The expressions of CRYΔ in the DN1ps only [29] did not result in faster behavioral rhythms, showing that the shorter period originates from a different group of neurons (Table S3); see Figure 2C for a possible scenario.

### Network Perturbations Can Drive Changes in the Period of Locomotor Activity Independently of Development, although Ontogeny Is Not without Effect

**Figure 5:**
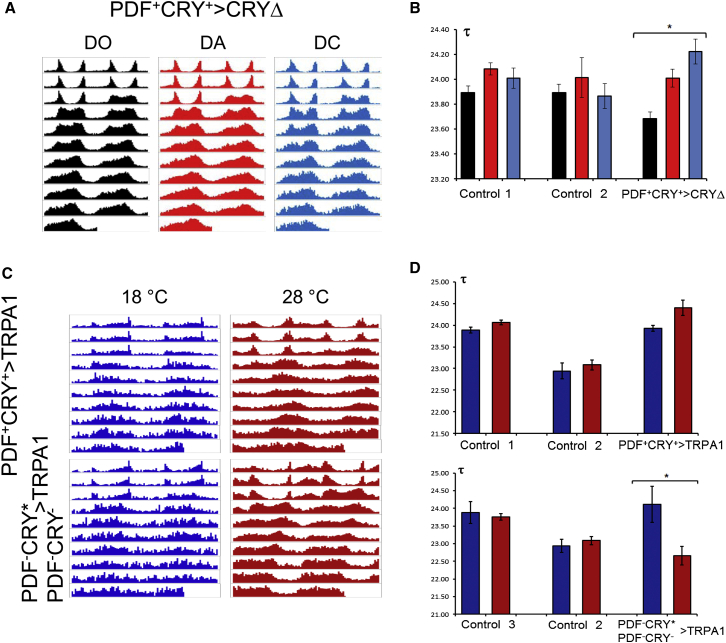
Network Perturbations Independently of Development (A and B) Expression of CRYΔ in PDF^+^CRY^+^ neurons after activation of the *Geneswitch* system by the drug RU486. (A) Average locomotor activity profiles (3 days LD, 8 days DD) of *Geneswitch* PDF^+^CRY^+^ > CRYΔ flies that were never subjected to drug treatment (DO), that were subjected to treatment as adults only (DA), or that were exposed to the drug since early development (DC). (B) The period of locomotor activity of *Geneswitch* PDF^+^CRY^+^ > CRYΔ flies and their parental controls were compared across the three different treatments: DO (black), DA (red), and DC (blue). ANOVA showed a nonsignificant effect of genotype (F_2,477_ = 0.554, p = 0.58) but a significant effect of treatment (F_2,477_ = 7.22, p < 0.01) and (asterisk) of the interaction term (genotype × treatment, F_4,477_ = 2.53, p = 0.04). Post-hoc analyses revealed significant differences comparing DA (Bonferroni, p < 0.01) and DC (Bonferroni, p = 0.04) to DO but not between DA and DC (Bonferroni, p = 1). Genotypes: *Geneswitch* PDF^+^CRY^+^ > CRYΔ, *w, UAS-cryΔ14.6; UAS-CD8GFP/+; Pdf-GS/+*; Control 1, *w; UAS-CD8GFP/+; Pdf-GS/+*; Control 2, *w, UAS-cryΔ14.6; +/+; +/+*. See also Table S4. (C and D) Expression of the temperature sensitive cation channel TRPA1 in PDF^+^CRY^+^ and in PDF^−^CRY^∗^ ∩ PDF^−^CRY^−^ cells under restrictive (18°C) and permissive (28°C) temperature. (C) Average locomotor activity profiles (3 days LD, 7 days DD) of both genotypes under both conditions. (D) The period of locomotor activity of PDF^+^CRY^+^ > TRPA1 (top) and PDF^−^CRY^∗^ ∩ PDF^−^CRY^−^ > TRPA1 (bottom) flies were compared, at the two temperatures (18°C, blue and 28°C, red), to their parental controls. The increase in period length for PDF^+^CRY^+^ > TRPA1 flies at higher temperature did not reach significance compared to controls as ANOVA showed a significant effect of genotype (F_2,104_ = 57.69, p < < 0.01) and temperature (F_1,104_ = 9.35, p < 0.01) but not of the interaction term (genotype × temperature, F_2,104_ = 1.45, p = 0.24). Conversely, we observed a significant decrease (asterisk) in period length for PDF^−^CRY^∗^ ∩ PDF^−^CRY^−^ > TRPA1 flies at higher temperature (ANOVA, genotype, F_2,75_ = 6.22, p < 0.01; temperature, F_1,75_ = 6.20, p = 0.02; genotype × temperature, F_2,75_ = 6.32, p < 0.01). Genotypes: PDF^+^CRY^+^ > TRPA1, *yw; Pdf-GAL4/+; +/UAS-TrpA1*; Control 1, *yw; Pdf-GAL4/+; +/+*; Control 2 *w; +/+; +/ UAS-TrpA1*; PDF^−^CRY^∗^ ∩ PDF^−^CRY^−^ > TRPA1 *yw; tim-GAL4/+; cry-GAL80*_*2e3m*_*/ UAS-TrpA1*; Control 3, *w; tim-GAL4/+; cry-GAL80*_*2e3m*_*/+*. See also Table S4.

The behavioral changes discussed above might depend on development. We employed *Pdf*-*Geneswitch* (*Pdf-GS*) [30] to trigger drug-dependent expression of CRYΔ in PDF^+^CRY^+^ neurons either in adults only (acute) or through the whole development (chronic). For acute treatment, flies were fed for 48 hr from the day of eclosion with medium supplemented with either 200 μg/ml of the drug RU486 (induction) or with an equal volume of vehicle (80% ethanol, no induction). Note, all flies were raised on this medium and were then examined (on their respective medium) for locomotor activity rhythms. For chronic treatment, flies were exposed to either drug or vehicle during their whole life and adult locomotor activity was examined accordingly. As controls, we tested (heterozygous) driver and effector flies raised under acute or chronic exposure to drug or on vehicle only (Table S4). Under both treatment conditions, we observed a significant, albeit small, lengthening of the period of locomotor activity for CRYΔ-overexpressing flies compared to controls (Figures 5A and 5B).

As a further test we directed the expression of the temperature-sensitive cation channel TRPA1 in PDF^+^CRY^+^ and in PDF^−^CRY^∗^ ∩ PDF^−^CRY^−^ cells (Figures 5C and 5D; Table S4). This channel is inactive at temperatures below 25°C in *Drosophila* and originally it was reported as being endogenously expressed in the adult brain in only about a dozen cells, which are not part of the circadian system [31]. However, recent evidence has shown that *trpa1* transcription does occur in at least some cells in each cluster of clock neurons but that the effects of *trpa1*-null mutations on rhythmic behavior are quite modest [32]. Flies overexpressing TRPA1 and (heterozygous) parental controls were raised at 18°C (TRPA1 is inactive) and then tested as adults at 18°C and 28°C (TRPA1 is active). Compared to controls, we would expect a longer and a shorter period for PDF^+^CRY^+^ > TRPA1 (*Pdf-GAL4* > *UAS-TrpA1*) and PDF^−^CRY^∗^ ∩ PDF^−^CRY^−^ > TRPA1 (*tim-GAL4* ∩ *cry-GAL80*_*2e3m*_ > *UAS-TrpA1*) flies at 28°C, respectively. For PDF^+^CRY^+^ > TRPA1 flies, the increase in period length with temperature was comparable to controls (Figures 5C and 5D). Conversely, we observed a significant decrease in period length for PDF^−^CRY^∗^ ∩ PDF^−^CRY^−^ > TRPA1 flies at higher temperature but not for controls (Figures 5C and 5D).

In summary, the manipulation of the circadian network limited to adults can affect the free-running period of locomotor activity. However, the modest effects obtained with adult PDF^+^CRY^+^ cells suggest that the ontogeny of the clock is particularly sensitive to the status of these neurons. Similar conclusions were reached by Depetris-Chauvin et al. [30] and Gorostiza and Ceriani [33]. Conversely, here we show that the PDF^−^CRY^∗^ and the PDF^−^CRY^−^ cells appear to be less sensitive to developmental effects (Figures 5C and 5D).

### Confirming the Model

Stoleru et al. [13] had shown that overexpression of a constitutive active form of the kinase SHAGGY (SGG), although able to shorten the period of locomotor activity when driven in PDF^+^CRY^+^ cells, was unable to elicit a behavioral effect when directed in PDF^−^CRY^∗^ ∩ PDF^−^CRY^−^ neurons [13]. This was in spite of causing a faster molecular oscillation in some cells, for instance the DN2s [13], and was considered evidence that those cells are not relevant for free-running behavior. This explanation contradicts our model, so we investigated further.

**Figure 6:**
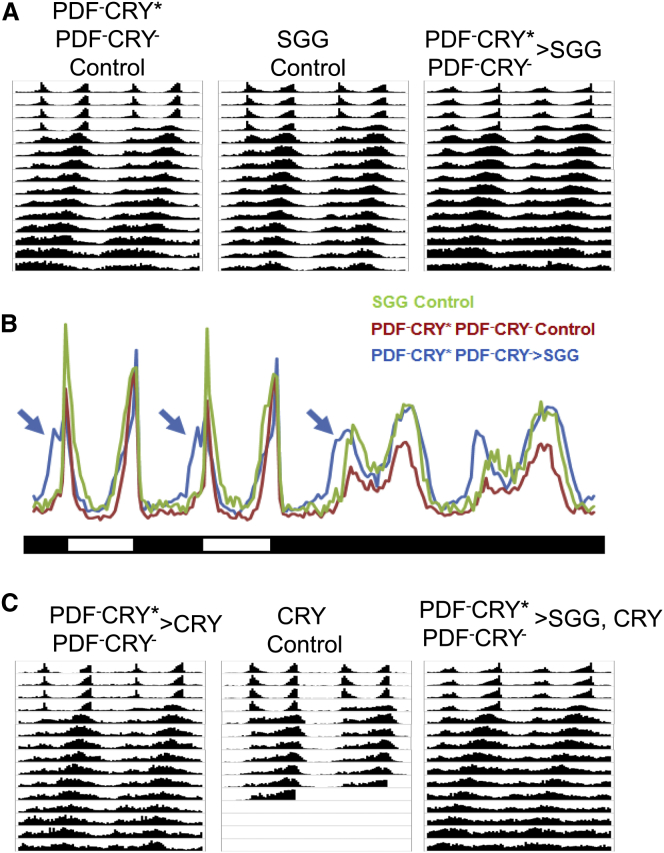
Ectopic Expression of CRY Reveals a Functional Interaction with SGG (A) Average locomotor activity profiles (4 days LD, 11 days DD) of PDF^−^CRY^∗^ ∩ PDF^−^CRY^−^ > SGG flies and controls. (B) Same data as in (A) but limited to the last 2 days of LD and first 2 days of DD. The profile for PDF^−^CRY^∗^ ∩ PDF^−^CRY^−^ > SGG flies (blue) shows earlier anticipation of the dark-to-light transitions (blue arrows) compared to the other genotypes (PDF^−^CRY^∗^ ∩ PDF^−^CRY^−^ Control, red and SGG Control, green). (C) Average locomotor activity profiles (4 days LD, 11 days DD) of PDF^−^CRY^∗^ ∩ PDF^−^CRY^−^ > CRY, CRY Control and PDF^−^CRY^∗^ ∩ PDF^−^CRY^−^ > SGG, CRY flies. Only the latter genotype showed a shorter period of locomotor activity (see Table S5). Genotypes: PDF^−^CRY^∗^ ∩ PDF^−^CRY^−^ > SGG, *w, UAS-sgg; tim-GAL4/+; cry-GAL80*_*2e3m*_*/ +*; SGG Control, *w, UAS-sgg; +/+; +/+*; PDF^−^CRY^∗^ ∩ PDF^−^CRY^−^ Control, *w; tim-GAL4/+; cry-GAL80*_*2e3m*_*/+*; PDF^−^CRY^∗^ ∩ PDF^−^CRY^−^ > CRY, *w; tim-GAL4/+; cry-GAL80*_*2e3m*_*/UAS-HAcry16.1*; CRY Control, *w; +/+; +/UAS-HAcry16.1*; PDF^−^CRY^∗^ ∩ PDF^−^CRY^−^ > SGG, CRY, *w, UAS-sgg; tim-GAL4/+; cry-GAL80*_*2e3m*_*/UAS-HAcry16.1*. See also Table S5.

PDF^−^CRY^∗^ ∩ PDF^−^CRY^−^ > SGG (*tim-GAL4* ∩ *cry-GAL80*_*2e3m*_ > *UAS-sgg*) flies showed a free-running period of locomotor activity no different from controls, in agreement with previous results [13] (Figure 6A; Table S5). However, a visual inspection of the activity profiles revealed that these flies, both in LD and DD, showed earlier anticipation of the actual or subjective dark-to-light transition (Figure 6B). Perhaps this anticipation of phase reflects a tendency for the shorter rhythm to propagate to the network, which instead is prevented by other neuronal groups. Previous work has suggested that there is a physical and functional interaction between CRY and SGG [14]. There is little or no CRY in PDF^−^CRY^∗^ and PDF^−^CRY^−^ neurons, which could preclude SGG from exerting any robust effect. According to our model, the PDF^−^CRY^∗^ repress the PDF^+^CRY^+^ cells; thus, increasing the output of the former should reduce the resistance of the network to the propagation of the faster rhythm. We coexpressed CRY and SGG and indeed observed a faster free-running period of locomotor activity in PDF^−^CRY^∗^ ∩ PDF^−^CRY^−^ > SGG, CRY (*tim-GAL4* ∩ *cry-GAL80*_*2e3m*_ > *UAS-sgg, UAS-cry*) but not in PDF^−^CRY^∗^ ∩ PDF^−^CRY^−^ > CRY (*tim-GAL4* ∩ *cry-GAL80*_*2e3m*_ > *UAS-cry*) flies (Figure 6C; Table S5). These results reveal that the DN2s are indeed part of the circuit that generates self-sustained behavior, although they do not exclude that the establishment of a faster rhythm might require additional PDF^−^CRY^−^ neurons. Implicitly, they also contradict the view of a defined hierarchical organization of the clock under constant conditions.

Considering all results reported above, we favor a model based on a flexible network of circadian neurons, which operates under any environmental condition. Our model complements and extends evidence by others of network organization as opposed to hierarchical dominance among neurons under LD and DD conditions [34, 35, 36].

### Conclusions

We have used genetic manipulations limited to discrete groups of neurons to unveil the logic governing self-sustained rhythmic locomotor activity in *Drosophila*. Activity rhythms and observations of molecular cycling in defined cellular groups consistently support a model (Figure 2) where endogenous behavioral rhythmicity is based upon synchronization between PDF^+^CRY^+^ and PDF^−^CRY^−^ cells. This requires modulation of the PDF^−^CRY^−^ cells by the PDF^+^CRY^+^ neurons, largely but not exclusively via PDF signaling, and multistep feedback adjustment of the latter group of neurons by the former. The behavioral outcomes of manipulations that alter but do not destroy the equilibrium of the network reveal that the PDF^+^CRY^+^ cells have a tendency for driving rhythms longer than 24 hr whereas some PDF^−^CRY^−^ cells promote rhythms shorter than 24 hr. However, the organization of the network is such that wild-type flies reach a combined ∼24 hr oscillation. This requires the PDF^−^CRY‡ and the PDF^−^CRY^∗^ cells that tune, through inhibitory interactions, the relative contribution of the other two groups of neurons to the network. We note that the interposition of the PDF^−^CRY‡ and the PDF^−^CRY^∗^ cells adds delay and signal amplification to the feedback regulation that links the PDF^+^CRY^+^ to the PDF^−^CRY^−^ neurons and vice versa.

In conclusion, we have shown that the endogenous behavioral period reflects the nonadditive interplay of all clock cells and not the dominant action of a single group of neurons (i.e., the s-LNvs), as previously suggested. Second, our overall results suggest that intercellular coupling is fundamental for synchronizing different cellular oscillators in a coherent clock, and therefore additional elements must complement the known molecular model focused around transcriptional regulators. Finally, we have shown that excitatory and inhibitory interactions are instrumental for changing the strength and the time delay in the coupling among neurons, resulting in different (collective) behavioral periods. This suggests that contrary to current belief, the period of circadian rhythms is not a fixed feature genetically encoded in a group of neurons. We propose that it is an emerging property of a network of multiple, different circadian oscillators and that it is the wiring of the system, whose logic is genetically encoded, that determines period length. Perhaps not surprisingly, the same principles of negative feedback with amplification and delay, which are central to the TTL model, re-emerge in the constitution of the clock at the intercellular level. We hope this view will inspire formal modeling of the *Drosophila* circadian network. The development of novel tools will be required to investigate how the known molecular mechanisms translate into different cellular features and for the experimental validation of the “logical” connections we postulate in our model. These matters will be the subject of our future work.

## Author Contributions

S.D. and E.R. generated the hypotheses. S.D., C.N.H., and E.R. designed the experiments. S.D., C.N.H., Ö.Ö., M.H., and E.R. performed experiments and analyzed data. S.D., C.P.K., and E.R. wrote the manuscript.

## Supplemental Information

Document S1. Figures S1–S3, Tables S1–S5, and Supplemental Experimental Procedures

Document S2. Article plus Supplemental Information
