# Supplementary material for: The Logic of Circadian Organization in Drosophila
Source: Curr Biol. 2014 Oct 6;24(19):2257–66. doi: 10.1016/j.cub.2014.08.023 (PMC4188814; doi:10.1016/j.cub.2014.08.023)
Supplement: Document S1. Figures S1–S3, Tables S1–S5, and Supplemental Experimental Procedures [file mmc1.pdf]

Current Biology, Volume *24*

Supplemental Information

The Logic of Circadian Organization in *Drosophila*

Stephane Dissel, Celia N. Hansen, Özge Özkaya, Matthew Hemsley, Charalambos P. Kyriacou, and Ezio Rosato

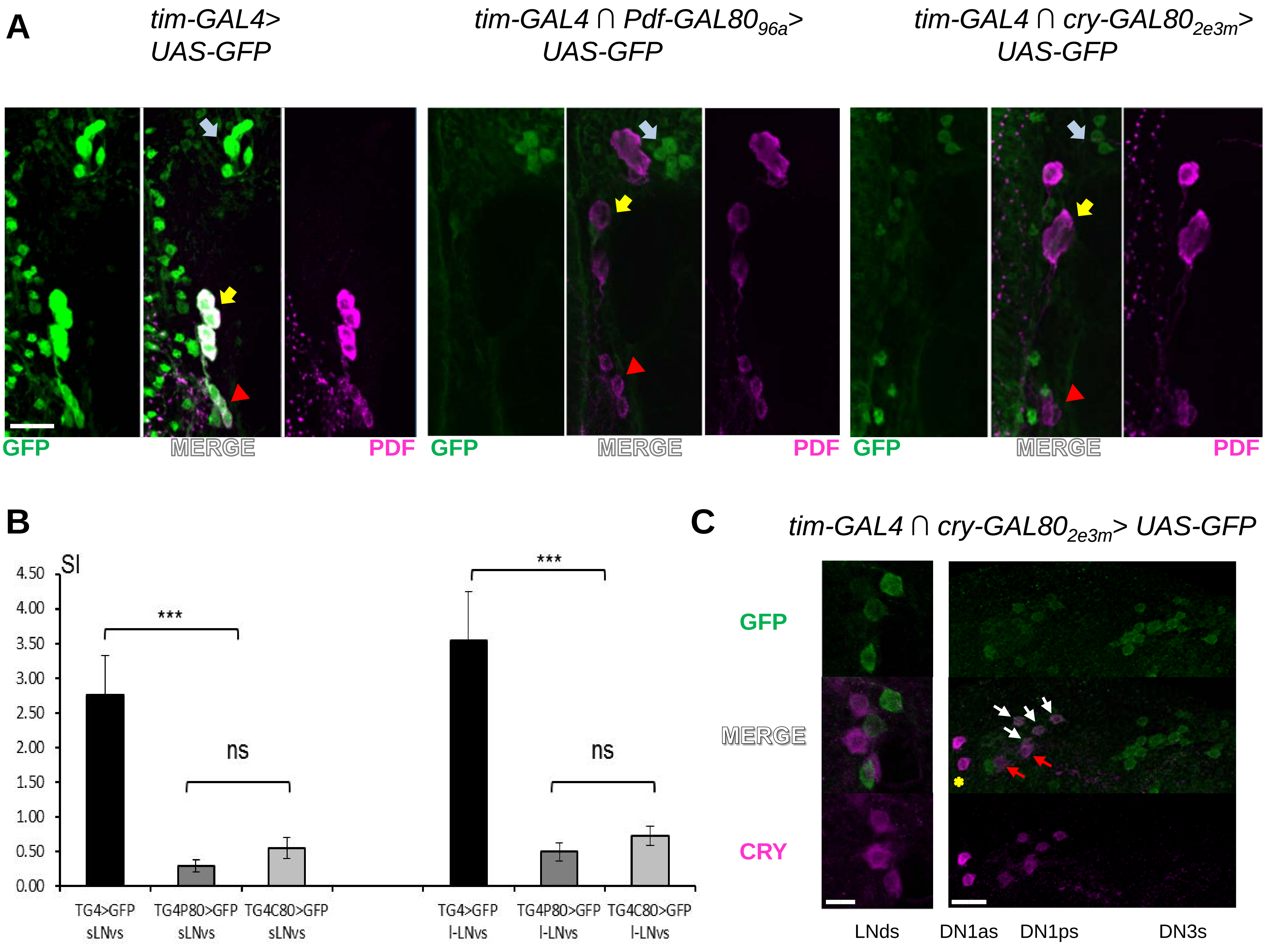
A
tim-GAL4> UAS-GFP
tim-GAL4 ∩ Pdf-GAL80$_{96a}$> UAS-GFP
tim-GAL4 ∩ cry-GAL80$_{2e3m}$> UAS-GFP
GFP
MERGE
PDF
B
SI
4.50
4.00
3.50
3.00
2.50
2.00
1.50
1.00
0.50
0.00
***
ns
TG4>GFP sLNvs
TG4P80>GFP sLNvs
TG4C80>GFP sLNvs
TG4>GFP l-LNvs
TG4P80>GFP l-LNvs
TG4C80>GFP l-LNvs
C
tim-GAL4 ∩ cry-GAL80$_{2e3m}$> UAS-GFP
GFP
MERGE
CRY
LNds
DN1as
DN1ps
DN3s

Figure S1. *Pdf-GAL8096a* and *cry-GAL80*$_{2e3m}$ mediated repression of *tim-GAL4* activity in clock neurons. Related to Figure 1.

(A) Anti-GFP immunoreactivity (green) in *tim-GAL4>UAS-GFP*, *tim-GAL4* ∩ *Pdf-GAL80*$_{96a}$*>UAS-GFP* and *tim-GAL4* ∩ *cry-GAL80*$_{2e3m}$*>UAS-GFP* flies. Anti-PDF staining (magenta) was used as a cellular marker. Intense GFP staining of the s-LNvs (red arrowheads) and l-LNvs (yellow arrows) was observed in *tim-GAL4>UAS-GFP* but not in the two GAL80 expressing genotypes. Six LNds (blue arrows) were observed in *tim-GAL4>UAS-GFP* and *tim-GAL4*, *Pdf-GAL80*$_{96a}$*>UAS-GFP* flies. Unexpectedly, three LNds were consistently observed in *tim-GAL4*, *cry-GAL80*$_{2e3m}$*>UAS-GFP* flies. Size bar = 20 µm.

(B) Anti-GFP immunoreactivity was quantified in s-LNvs (right) and l-LNvs (left) of *tim-GAL4>UAS-GFP*, *tim-GAL4* ∩ *Pdf-GAL80*$_{96a}$*>UAS-GFP* and *tim-GAL4* ∩ *cry-GAL80*$_{2e3m}$*>UAS-GFP* flies and reported as Staining Index (SI); the error bars correspond to the standard error of the mean (SEM). SI was calculated as described in Materials and Methods, with the exception that values were not normalized for the expected number of cells according to cell type. The two GAL80 expressing strains showed a significant reduction in SI compared to *tim-GAL4>UAS-GFP* flies for both cell types (ANOVA, Genotype $F_{2,66}$=39.79, P<<0.01; Cell type $F_{1,66}$=2.10, P=0.15, Genotype*Cell type $F_{2,66}$=0.52, P=0.60). Importantly, there was no difference between the GAL80 carrying strains (Bonferroni post-hoc: *tim-GAL4>UAS-GFP vs. tim-GAL4* ∩ *cry-GAL80>UAS-GFP*, P<<0.01; *tim-GAL4>UAS-GFP vs. tim-GAL4* ∩ *Pdf-GAL80>UAS-GFP*, P<<0.01; *tim-GAL4* ∩ *Pdf-GAL80>UAS-GFP vs. tim-GAL4* ∩ *cry-GAL80>UAS-GFP*, P=1.00). We analysed 10, 14, and 12 hemispheres for the genotypes *tim-GAL4>UAS-GFP*, *tim-GAL4* ∩ *Pdf-GAL80>UAS-GFP* and *tim-GAL4* ∩ *cry-GAL80>UAS-GFP*, respectively.

(C) Published observations suggest that the *cry* promoter is expressed in all 6 LNds in *cry-GAL4* transgenics [11, 18], but that the CRY protein is robustly detected in only 3 LNds in wild type flies [7]. This would suggest that the GFP-positive and negative LNds in *tim-GAL4* ∩ *cry-GAL80*$_{2e3m}$*>UAS-GFP* as seen in (A) correspond to the CRY immunonegative and immunopositive LNds, respectively. Anti-GFP (green) and anti-CRY (magenta) immunoreactivities did not overlap in the LNds of *tim-GAL4* ∩ *cry-GAL80>UAS-GFP* flies, verifying this hypothesis (left panel, size bar = 5 µm.). We also compared anti-GFP and anti-CRY staining in the dorsal brain (right panel, size bar = 15 µm.). Two DN1as (yellow asterisk) and two DN1ps (red arrows) showed anti-CRY staining only (*cry* expression is high). Four DN1ps (white arrows) were labelled by both anti-GFP and anti-CRY antibodies (*cry* expression is low). The DN3s showed anti-GFP immunoreactivity only (*cry* expression is very low or absent).

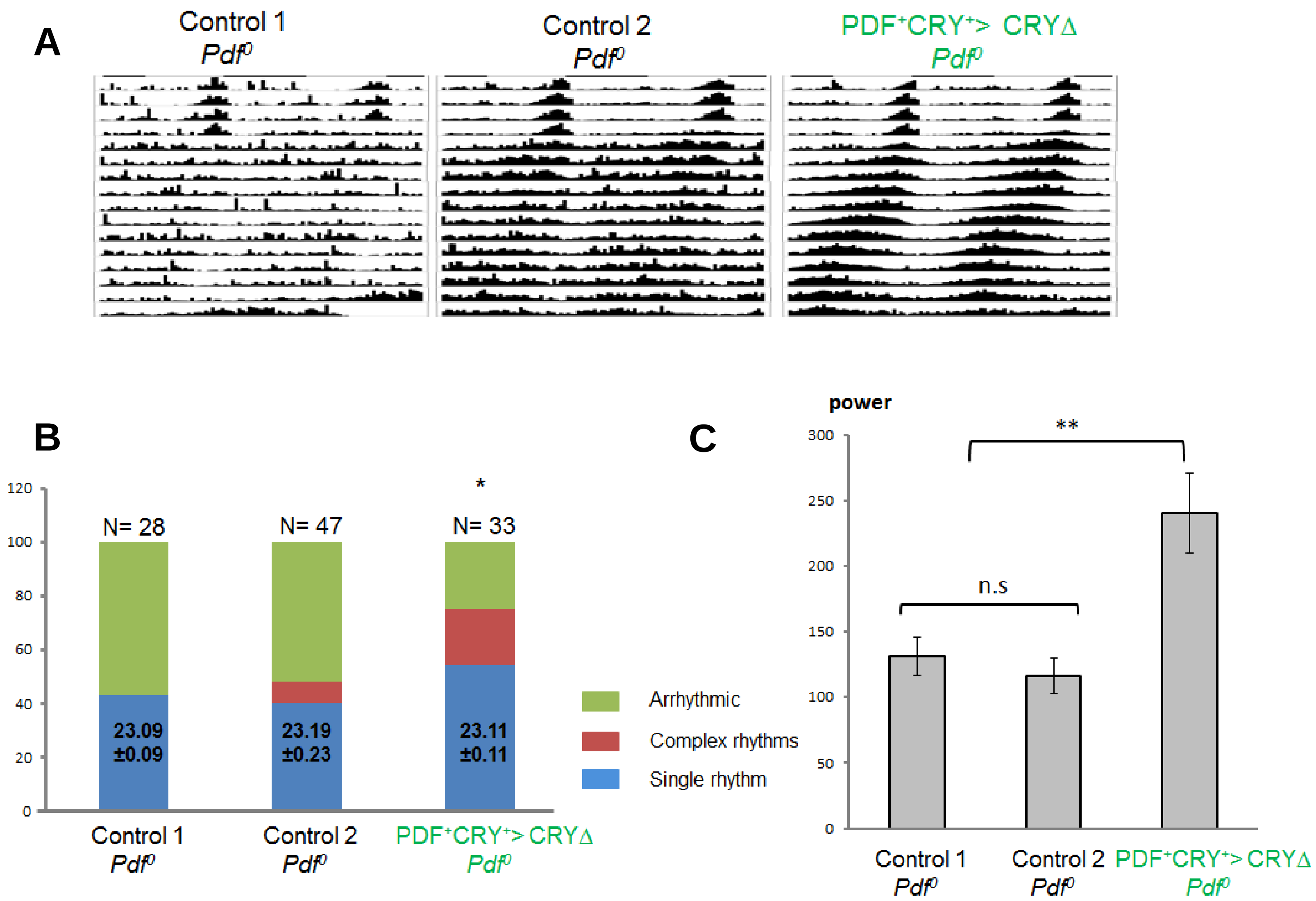

A
Control 1
Pdf0
Control 2
Pdf0
PDF+CRY+> CRYΔ
Pdf0
B
120
100
80
60
40
20
0
N= 28
N= 47
*
N= 33
23.09
±0.09
23.19
±0.23
23.11
±0.11
Arrhythmic
Complex rhythms
Single rhythm
Control 1
Pdf0
Control 2
Pdf0
PDF+CRY+> CRYΔ
Pdf0
C
power
300
250
200
150
100
50
0
**
n.s
Control 1
Pdf0
Control 2
Pdf0
PDF+CRY+> CRYΔ
Pdf0

Figure S2.Expression of CRYΔ in PDF+CRY+ neurons improves behavioural rhythmicity in *Pdf*^{0} flies suggesting a link between PDF+CRY+ and PDF-CRY- cells. Related to Figure 2.
Although mainly arrhythmic, a moderate proportion of *Pdf*^{0} mutants are still rhythmic in DD but have a (~ 1 h) shorter activity period [16], suggesting that faster PDF-CRY- neurons might be responsible for it. A significant increase in the number of rhythmic individuals (from ~30% to ~60%) has been reported for PDF+CRY+>NaChBac (*Pdf*^{0}) flies (*Pdf-GAL4, Pdf*^{0}*/Pdf*^{0}*>UAS-NaChBac*) [23]. We confirmed those results by testing PDF+CRY+>CRYΔ (*Pdf*^{0}) mutants (*Pdf-GAL4, Pdf*^{0}*/Pdf*^{0}*>UAS-cryΔ*) and parental controls.
(A) Average locomotor activity profiles of flies showing 4 days in LD 12:12 and 12 days in DD. The majority of PDF+CRY+>CRYΔ (*Pdf*^{0}) flies sustained rhythmic behaviour under DD, contrary to *Pdf*^{0} controls.
(B) Each column shows the percentage of single rhythm (blue) complex rhythms (red) and arrhythmic flies (green) per genotype. The proportion of rhythmic (single+complex) flies was significantly higher for the PDF+CRY+>CRYΔ (*Pdf*^{0}) genotype than controls (two-tailed Fisher's exact test, P<0.01), this is indicated by an asterisk. The (average ±SEM) period of locomotor activity of single rhythm flies is shown in the blue sections. There were no significant differences in period among genotypes (ANOVA, $F_{2,46}$=0.10, P=0.91).
(C) Columns represent the mean power (robustness) calculated for single rhythm flies of each genotype. Error bars correspond to SEM. The power was significantly higher for the PDF+CRY+>CRYΔ (*Pdf*^{0}) genotype than controls (ANOVA, $F_{2,46}$=9.95, P<0.001. Bonferroni post-hoc comparisons: Control1 (*Pdf*^{0}) *vs*. Control2 (*Pdf*^{0}), P=1.00. Control1 (*Pdf*^{0}) *vs*. PDF+CRY+>CRYΔ (*Pdf*^{0}), P=0.006. Control2 (*Pdf*^{0}) *vs*. PDF+CRY+>CRYΔ (*Pdf*^{0}), P<0.001).
Genotypes: PDF+CRY+>CRYΔ (*Pdf*^{0}): *w, UAS-cryΔ14.6; Pdf-GAL4/+; Pdf*^{01}*/Pdf*^{01}. Control1 *Pdf*^{0}: *w; Pdf-GAL4/+; Pdf*^{01}*/Pdf*^{01}; Control2 *Pdf*^{0}: *w, UAS- cryΔ14.6; +/+; Pdf*^{01}*/Pdf*^{01}. N=total number of flies. The same number of flies contributed to the activity profiles shown in (A).
Thus, in *Pdf*^{0} mutants the overexpression of NaChBac or CRYΔ restricted to the PDF+CRY+ cells (*Pdf-GAL4* driver) results in more rhythmic flies with shorter behavioural periods. The latter suggest the involvement of fast PDF-CRY- neurons, although the *Pdf-GAL4* driver does not directly target them. We conclude that a positive, PDF-independent connection likely links the PDF+CRY+ and the PDF-CRY- cells. In support of this hypothesis, we notice that the s-LNvs, which project to the dorsal area of the brain where the PDF-CRY- cells are located, express a small neurotransmitter in addition to PDF [S1, S2]. This signalling mechanism seems important as preferential activation of the s-LNvs through a membrane tethered PDF molecule (PDF+CRY+>t-PDF stimulates predominantly the s-LNvs because the l-LNvs express very little or no PDFR, see ref. [37]) results in short period rhythms [S1].
Furthermore, these data suggest that CRYΔ is an activator of neuronal function but weaker than NaChBac, which possibly explains why the short period behavioural component is absent in PDF+CRY+>CRYΔ flies (see also Figures S3).

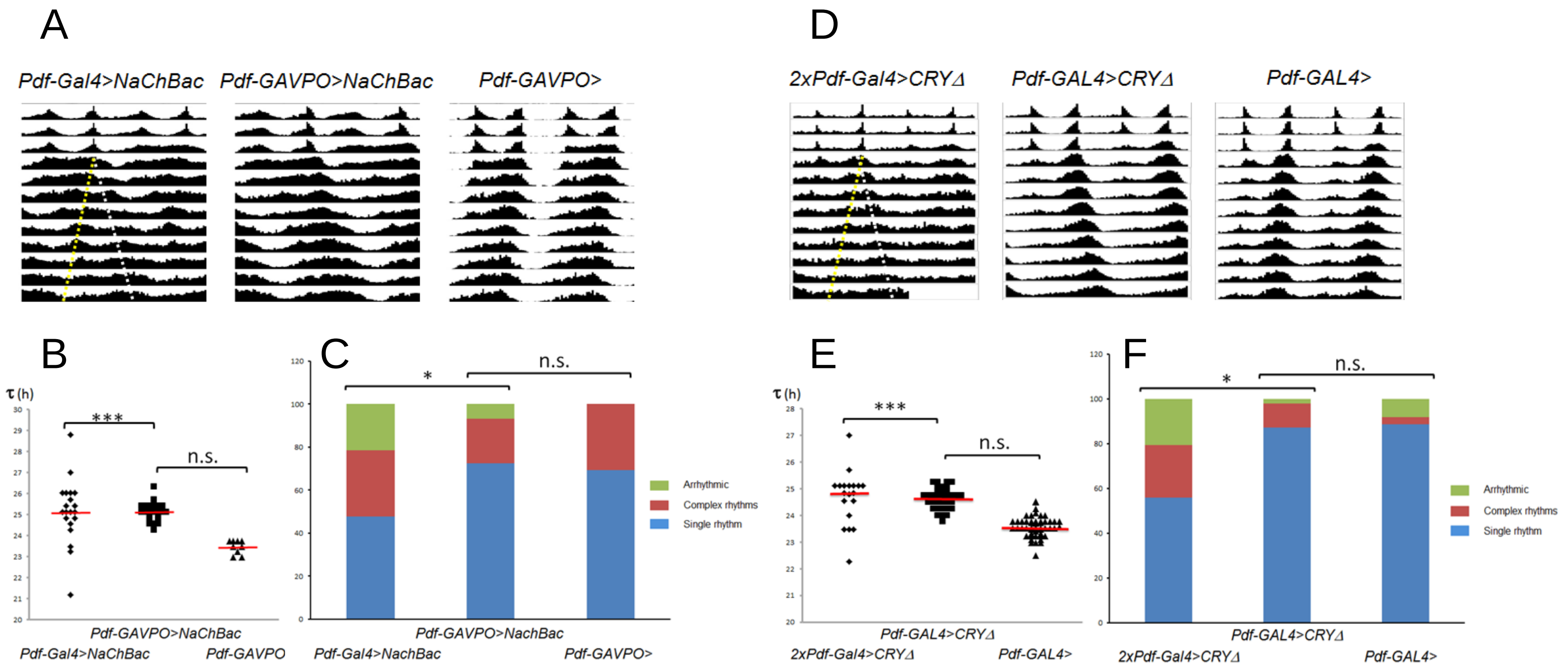
A
D
Pdf-Gal4>NaChBac
Pdf-GAVPO>NaChBac
Pdf-GAVPO>
2xPdf-Gal4>CRYΔ
Pdf-GAL4>CRYΔ
Pdf-GAL4>
B
C
E
F
τ (h)
***
n.s.
*
Arrhythmic
Complex rhythms
Single rhythm
Pdf-GAVPO>NaChBac
Pdf-Gal4>NaChBac
Pdf-GAVPO
Pdf-GAVPO>NachBac
Pdf-Gal4>NachBac
Pdf-GAVPO>
Pdf-GAL4>CRYΔ
2xPdf-Gal4>CRYΔ
Pdf-GAL4>

Figure S3. NaChBac and CRYΔ generate similar phenotypes when expressed in PDF+CRY+ neurons, but at different levels. Related to Figure 3.

(A-B) To reduce the expression of NachBac we implemented in *Drosophila* a recently described chimeric GAL4-VIVID-P65 transcription factor called GAVPO that requires induction by blue light to initiate UAS-dependent transcription. GAVPO can only dimerize (which is required for transcriptional activity) in the presence of light but the dimer is then stable until cleared by cellular turnover. [S3]. We generated a *Pdf-GAVPO* line and we used it to drive NaChBac expression under LD 12:12; then we released the flies into DD for nine days so that active GAVPO would not be further produced.

(A) Average locomotor activity profiles of NaChBac expressing and control flies showing 3 days in LD 12:12 and 9 days in DD. The DD activity profile of *Pdf-GAL4>NaChBac* flies showed a long and a short activity components. The latter was lost when NaChBac overexpression was reduced in DD using the *Pdf-GAVPO* driver. *Pdf-GAVPO>* flies were included as a control. The white and the yellow hatched lines indicate the long and the short activity components, respectively.

(B) Scatter plot showing the distribution of free run single rhythm periodicities for *Pdf-GAL4>NaChBac*, *Pdf-GAVPO>NaChBac* and *Pdf-GAVPO>* flies. *Pdf-GAL4>NaChBac* had significantly higher variance (two-tailed F-test, P<<0.01) than *Pdf-GAVPO>NaChBac* flies, whereas the latter were not significantly different from controls (two-tailed F-test, P=0.31). Red Bar = median.

(C) Columns represent the percentage of single rhythm (blue), complex rhythm (red) and arrhythmic (green) individuals for *Pdf-GAL4>NaChBac*, *Pdf-GAVPO>NaChBac* and *Pdf-GAVPO>* flies in DD. Reducing DD expression of NaChBac *via Pdf-GAVPO* resulted in an increased proportion of single rhythm flies, as expected (one-tailed Fisher's exact test, P=0.03). The proportions of single rhythm flies were not different between *Pdf-GAVPO>NaChBac* and *Pdf-GAVPO>* controls (one-tailed Fisher's exact test, P=0.55).

Genotypes: *yw; Pdf-GAL4/UAS-NaChBac4;+/+* (N=42), *w; +/UAS-NaChBac4; Pdf-GAVPO/+* (N=29), *w; +/+; Pdf-GAVPO/+* (N=13). All genotypes were tested in a single, parallel experiment.

(D-F) We increased the expression of CRYΔ in PDF+CRY+ neurons by combining two copies of the driver, namely *2xPdf-GAL4.*

(D) Average locomotor activity profiles of CRYΔ expressing and control flies showing 3 days in LD 12:12 and 9 days in DD. The DD activity profile of *Pdf-GAL4>CRYΔ* flies showed a long activity components. An additional short period component was acquired when CRYΔ overexpression was increased by virtue of two copies of the *Pdf-GAL4* driver. *Pdf-GAL4>* flies were included as a control. The white and the yellow hatched lines indicate the long and the short activity components, respectively.

(E) Scatter plot showing the distribution of free run single rhythm periodicities for 2x*Pdf-GAL4>CRYΔ*, *Pdf-GAL4>CRYΔ* and *Pdf-GAL4>* flies. 2x*Pdf-GAL4>CRYΔ* had significantly higher variance (two-tailed F-test, P<<0.01) than *Pdf-GAL4>CRYΔ* flies, whereas the latter were not significantly different from controls (two-tailed F-test, P=0.90). Red Bar = median.

(F) Columns represent the percentage of single rhythm (blue), complex rhythm (red) and arrhythmic (green) individuals for 2x*Pdf-GAL4>CRYΔ*, *Pdf-GAL4>CRYΔ* and *Pdf-GAL4>* flies. Increasing CRYΔ expression via 2x*Pdf-GAL4* resulted in a reduced proportion of single rhythm flies, as expected (one-tailed Fisher's exact test, P=0.002). The proportions of single rhythm flies were not different between *Pdf-GAL4>CRYΔ* flies and *Pdf-GAL4>* controls (one-tailed Fisher's exact test, P=0.53).

Genotypes: *w, UAS-cryΔ14.6; Pdf-GAL4/Pdf-GAL4; +/+* (N=34), *w, UAS-cryΔ14.6; Pdf-GAL4/+; +/+* (N=47*), *yw; Pdf-GAL4/+; +/+* (N=61*). * Data are the same as in Table S1.

Table S1. Locomotor activity period (τ) after overexpression of CRYΔ in combinations of PDF+/- and CRY+/- neurons. Related to Figure 1

Genotype	Over-expressing	N (n)	τ ± S.E.M.	SR(%)	CR(%)	AR(%)	Neurons
w, UAS-cryΔ14.6; tim-GAL4/+; +/+	CRYΔ	48 (3)	24.90 ± 0.07[a1]	71	25	4	PDF^{+} CRY^{+}, PDF^{-} $CRY^{‡}$,
w; tim-GAL4/+; UAS-cryΔ4.1/+	CRYΔ	38 (3)	25.75 ± 0.22	84	11	5	PDF^{-} CRY^{*}, PDF^{-} CRY^{-}
w, UAS-HAcryΔ15.3; tim-GAL4/+; +/+	CRYΔ	27 (2)	25.44 ± 0.16	89	11	0	
w, UAS-cryΔ14.6; Pdf-GAL4/+; +/+	CRYΔ	47 (3)	24.64 ± 0.06[a2]	87	11	2	$PDF^{+}$$CRY^{+}$
w; Pdf-GAL4/+;UAS-cryΔ4.1/+	CRYΔ	32 (2)	25.13 ± 0.12	94	3	3	
w, UAS-HAcryΔ15.3; Pdf-GAL4/+; +/+	CRYΔ	18 (1)	26.28 ± 0.20	72	22	6	
w, UAS-cryΔ14.6; tim-GAL4/+; cry-GAL80$_{2e3m}$/+	CRYΔ	54 (3)	22.50 ± 0.06[a3]	79	15	6	PDF^{-} CRY^{*}, PDF^{-} CRY^{-}
w; tim-GAL4/+; cry-GAL80$_{2e3m}$/UAS-cryΔ4.1	CRYΔ	48 (4)	22.77 ± 0.08	77	6	17	
w,UAS-HAcryΔ15.3; tim-GAL4/+;cry-GAL80$_{2e3m}$/+	CRYΔ	38 (2)	22.63 ± 0.06	68	11	21	
w, UAS-cryΔ14.6; Pdf-GAL80$_{96a}$/+; cry$_{13}$-GAL4/+	CRYΔ	33 (2)	24.08 ± 0.08[a4]	88	9	3	PDF^{-} $CRY^{‡}$, PDF^{-} CRY^{*}
w, UAS-cryΔ14.6; tim-GAL4/Pdf-GAL80$_{96a}$; +/+	CRYΔ	71 (5)	23.51 ± 0.09[a5]	69	28	3	PDF^{-} $CRY^{‡}$, PDF^{-} CRY^{*}, PDF^{-} CRY^{-}
w, UAS-cryΔ14.6; tim-GAL4, Pdf-GAL80/+; Pdf-GAL80/+	CRYΔ	27 (2)	23.66 ± 0.08[a5]	78	22	0	
w, UAS-cryΔ14.6; +/+; cry$_{13}$-GAL4/+	CRYΔ	67 (4)	24.67 ± 0.06[a6]	90	10	0	PDF^{+} CRY^{+}, PDF^{-} $CRY^{‡}$, PDF^{-} CRY^{*}
w, UAS-cryΔ14.6; +/+; +/+		46 (4)	23.78 ± 0.06[a7]	76	15	9	Controls
w; +/+; UAS-cryΔ4.1/+		47 (4)	23.81 ± 0.09	77	17	6	
w, UAS-HAcryΔ15.3;+/+; +/+		15 (1)	24.10 ± 0.12	100	0	0	
w; tim-GAL4/+; +/+		70 (7)	23.79 ± 0.05[a8]	82	11	7	
w; Pdf-GAL4/+; +/+		61 (5)	23.57 ± 0.05[a9]	89	3	8	

w; +/+; cry$_{13}$*-GAL4/+*	43 (3)	23.76 ± 0.05[a10]	86	12	2
w, UAS-cryΔ14.6; Pdf-GAL80$_{96a}$*/+; +/+*	24 (3)	23.93 ± 0.07[a11]	87	13	0
yw; tim-GAL4, Pdf-GAL80/+; Pdf-GAL80/+	38 (3)	23.84 ± 0.07[a12]	84	16	0
w; tim-GAL4/+; cry-GAL80$_{2e3m}$*/+*	98 (8)	23.50 ± 0.05[a13]	75	10	15

The period of locomotor activity was determined by Fourier (spectral) analysis [S4]. Only male flies were studied. **N**= total number of flies examined; n = number of independent replicates. **τ ± S.E.M.**, average period of locomotor activity ± standard error of the mean of flies showing a single period. **SR(%)**, percentage of flies showing a single period. **CR(%)**, percentage of flies showing complex rhythms. **AR(%)**, percentage of arrhythmic flies.

14.6 (X chromosome), 4.1 (3rd Chromosome) and 15.3 (X Chromosome) indicate three independent *UAS-cryΔ* insertions. *HAcryΔ15.3* carries at its 5' the sequence for production of the HA epitope tag.

ANOVA a1-a13, $F_{12,574}$ = 94.92, P<<0.01; Bonferroni post-hoc:

- PDF^{+} CRY^{+}, PDF^{-} CRY‡, PDF^{-} CRY*, PDF^{-} CRY^{-}>CRYΔ single rhythm flies had ~1h longer activity rhythms than controls, a1 ***vs*** a8, P<<0.001; a1 ***vs*** a7, P<<0.001
- PDF^{+} CRY^{+}>CRYΔ single rhythm flies had ~1h longer activity rhythms than controls, a2 ***vs*** a9, P<<0.001; a2 ***vs*** a7, P<<0.001
- PDF^{+} CRY^{+}, PDF^{-} CRY‡, PDF^{-} CRY*> CRYΔ single rhythm flies had ~1h longer activity rhythms than controls, a6 ***vs*** a10, P<<0.001; a6 ***vs*** a7, P<<0.001
- PDF^{+} CRY^{+}, PDF^{-} CRY‡, PDF^{-} CRY*, PDF^{-} CRY^{-}>CRYΔ were not different from PDF^{+} CRY^{+}>CRYΔ and PDF^{+} CRY^{+}, PDF^{-} CRY‡, PDF^{-} CRY*> CRYΔ single rhythm flies, a1 ***vs*** a2, P=0.63; a1 ***vs*** a6, P=0.82; a2 ***vs*** a6, P=1.00
- PDF^{-} CRY*, PDF^{-} CRY^{-}>CRYΔ single rhythm flies had ~1h shorter activity rhythms than controls, a3 ***vs*** a7, P<<0.001; a3 ***vs*** a13, P<<0.001
- PDF^{-} CRY‡, PDF^{-} CRY*, PDF^{-} CRY^{-}>CRYΔ single period flies were not different from controls, a5 ***vs*** a7, P=0.80; a5 ***vs*** a11, P=0.04; a5 ***vs*** a12, P=0.12
- PDF^{-} CRY‡, PDF^{-} CRY*>CRYΔ single period flies were not different from controls, a4 ***vs*** a10, P=0.14; a4 ***vs*** a11, P=1.00.

Table S2. Locomotor activity rhythms after manipulation of clock neurons. Related to Figure 3.

Genotype	Over-expressing	N (n)	τ ± S.E.M.	SR(%)	CR(%)	AR(%)	Neurons
yw; tim-GAL4/UAS-NaChBac4;+/+	NaChBac4	14 (1)		0	7	93	PDF^{+} CRY^{+}, PDF^{-} CRY‡,
yw; tim-GAL4/+;+/ UAS-NaChBac2	NaChBac2	16 (1)	24.04 ± 0.17	44	0	56	PDF^{-} CRY*, PDF^{-} CRY^{-}
w; tim-GAL4/ UAS-ClkΔ1;+/+	CLKΔ1	26 (2)		0	4	96	
yw; Pdf-GAL4/UAS-NaChBac4;+/+	NaChBac4	39 (2)	25.40 ± 0.18[a]	36	54	10	PDF^{+}CRY^{+}
yw; Pdf-GAL4/+;+/ UAS-NaChBac2	NaChBac2	26 (2)	26.28 ± 0.41[b]	42	50	8	
yw; Pdf-GAL4/+; +/ UAS-Kir2.1	KIR2.1	42 (2)	22.94 ± 0.10[i]	43	12	45	
yw; Pdf-GAL4/UAS-Kir2.1; +/+	KIR2.1	43 (3)	23.34 ± 0.08[j]	53	14	33	
yw, UAS-hid, UAS-rpr; Pdf-GAL4/+; +/+	HID, RPR	35 (1)	22.8 ± 0.12[l]	54	23	23	
yw; Pdf-GAL4/ UAS-ClkΔ1;+/+	CLKΔ1	35 (2)	23.15 ± 0.16[n]	48	29	23	
yw; Pdf-GAL4/ UAS-ClkΔ2;+/+	CLKΔ2	32 (3)	22.52 ± 0.24[o]	40	13	47	
w; tim-GAL/UAS-NaChBac4;cry-GAL80$_{2e3m}$*/+*	NaChBac4	24 (2)	23.06 ± 0.49[c]	13	8	79	PDF^{-} CRY*, PDF^{-} CRY^{-}
w; tim-GAL4/+;cry-GAL80$_{2e3m}$*/ UAS-NaChBac2*	NaChBac2	39 (2)	23.72 ± 0.16[d]	25	3	72	
yw, UAS-hid, UAS-rpr; tim-GAL4/+; cry-GAL80$_{2e3m}$ */+*	HID, RPR	18 (3)	24.12 ± 0.39[m]	28	11	61	
w; tim-GAL4/ UAS-ClkΔ1;cry-GAL80$_{2e3m}$*/+*	CLKΔ1	34 (3)	32.00	3	3	94	
w; tim-GAL4/ UAS-ClkΔ2;cry-GAL80$_{2e3m}$*/+*	CLKΔ2	16 (3)		0	0	100	
yw; tim-GAL4, Pdf-GAL80/UAS-NaChBac4; Pdf-GAL80/+	NaChBac4	52 (2)	24.36 ± 0.07[e]	81	17	2	PDF^{-} CRY‡, PDF^{-} CRY*, PDF^{-} CRY^{-}
yw; tim-GAL4, Pdf-GAL80/+; Pdf-GAL80/UAS-NaChBac2	NaChBac2	34 (2)	24.83 ± 0.07[f]	67	24	9	
yw; +/UAS-NaChBac4; cry$_{13}$*-GAL4/+*	NaChBac4	46 (2)	23.57 ± 0.37[g]	26	41	33	PDF^{+} CRY^{+}, PDF^{-} CRY‡, PDF^{-} CRY*
yw; +/+; cry$_{13}$*-GAL4/ UAS-NaChBac2*	NaChBac2	33 (2)	23.76 ± 0.35[h]	31	45	24	
w; +/ UAS-ClkΔ1; cry$_{13}$*-GAL4/+*	CLKΔ1	22 (2)	23.47 ± 0.13[p]	72	14	14	

w; +/ UAS-ClkΔ2; cry_{13}*-GAL4/+*	CLKΔ2	35 (2)	23.43 ± 0.04[q]	94	6	0	
w; +/ +; cry_{13}*-GAL4/ UAS-Kir2.1*	KIR2.1	32 (2)	23.60 ± 0.11[k]	78	13	9	
w; Pdf-GAL80$_{96a}$ */ UAS-ClkΔ1;* cry_{13}*-GAL4/+*	CLKΔ1	13 (2)	23.83 ± 0.07[r]	92	8	0	PDF$^-$ CRY‡, PDF$^-$ CRY*
yw; tim-GAL4/+; +/+		70 (7)	23.79 ± 0.05	72	11	7	Controls
yw; Pdf-GAL4/+; +/+		61 (5)	23.57 ± 0.05[a,b,i,j, l,n,o]	89	3	8	
yw; +/+; cry_{13}*-GAL4/+*		43 (2)	23.76 ± 0.05[g,h,k,p,q,r]	86	12	2	
w; tim-GAL4/+; cry-GAL80$_{2e3m}$*/+*		98 (8)	23.50 ± 0.05[c,d,m]	75	10	15	
yw; tim-GAL4, Pdf-GAL80/+; Pdf-GAL80/+		38 (3)	23.84 ± 0.07[e,f]	84	16	0	
w; +/ UAS-NaChBac4;+/+		22 (2)	24.11 ± 0.09 [a,c,e,g]	86	9	5	
w; +/+;+/ UAS-NaChBac2		24 (2)	24.08 ± 0.05 [b,d,f,h]	87	13	0	
w; +/+; +/ UAS-Kir2.1		12 (1)	23.22± 0.09[i,k]	92	0	8	
w; +/UAS-Kir2.1;+/+		22 (1)	23.67± 0.07[j]	91	9	0	
yw, *UAS-hid*, *UAS-rpr*; *+/+; +/+*		30 (1)	24.27 ± 0.15[l,m]	93	7	0	
w; +/UAS-ClkΔ1;+/+		35 (3)	23.64 ± 0.06[n,p,r]	94	3	3	
w; +/UAS-ClkΔ2;+/+		24 (4)	23.53 ± 0.06[o,q]	92	4	4	
w, UAS-cryΔ14.6; Pdf-GAL80$_{96a}$*/+; +/+*		24 (2)	23.93 ± 0.07[r]	87	13	0	

The period of locomotor activity was determined by Fourier (spectral) analysis [S4]. Only male flies were studied. **N** = total number of flies examined; n = number of independent replicates. **τ ± S.E.M.**, average period of locomotor activity ± standard error of the mean of flies showing a single period. **SR(%)**, percentage of flies showing a single period. **CR(%)**, percentage of flies showing complex rhythms. **AR(%)**, percentage of arrhythmic flies.

[a,b] PDF$^+$CRY$^+$>NaChBac single period flies had > 1h longer activity rhythms than controls (overall [a]ANOVA, $F_{2,84}$ = 98.42, P<<0.01; Bonferroni post-hoc *yw; Pdf-GAL4/UAS-NaChBac4;+/+* **vs** *yw; Pdf-GAL4/+; +/+*, P<<0.01, **vs** *w; +/ UAS-NaChBac4;+/+* , P<<0.01. Overall [b]ANOVA, $F_{2,83}$ = 103.24, P<<0.01; Bonferroni post-hoc: *yw; Pdf-GAL4/+;+/ UAS-NaChBac2* **vs** *yw; Pdf-GAL4/+; +/+*, P<<0.01, **vs** *w; +/+;+/UAS-NaChBac2* , P<<0.01).

[c,d] PDF$^-$ CRY*, PDF$^-$CRY$^-$>NaChBac single period flies had a tendency for shorter activity rhythms but neither line reached significance over both controls (overall [c]ANOVA, $F_{2,92} = 17.92$, P<<0.01; but Bonferroni post-hoc: *w; tim-GAL/UAS-NaChBac4;cry-GAL80*$_{2e3m}$*/+* **vs** *w; tim-GAL4/+; cry-GAL80*$_{2e3m}$*/+*, P=0.25, **vs** *w; +/ UAS-NaChBac4;+/+*, P<<0.01. Overall [d]ANOVA, $F_{2,99} = 15.89$, P<<0.01; but Bonferroni post-hoc: *w; tim-GAL4/+;cry-GAL80*$_{2e3m}$*/ UAS-NaChBac2* **vs** *w; tim-GAL4/+; cry-GAL80*$_{2e3m}$*/+*, P=0.38, **vs** *w; +/+;+/UAS-NaChBac2* , P=0.06).

[e,f] PDF$^-$ CRY‡, PDF$^-$ CRY*, PDF$^-$CRY$^-$>NaChBac single period flies had 0.5-1 h longer activity rhythms than controls. Differences were suggestive for one line and significant for the other (overall [e]ANOVA, $F_{2,90} = 13.40$, P<<0.01; but Bonferroni post-hoc: *yw; tim-GAL4, Pdf-GAL80/UAS-NaChBac4; Pdf-GAL80/+* **vs** *yw; tim-GAL4, Pdf-GAL80/+; Pdf-GAL80/+*, P<<0.01, **vs** *w; +/ UAS-NaChBac4;+/+*, P=0.10. Overall [f]ANOVA, $F_{2,73} = 55.34$, P<<0.01; Bonferroni post-hoc: *yw; tim-GAL4, Pdf-GAL80/+; Pdf-GAL80/UAS-NaChBac2* **vs** *yw; tim-GAL4, Pdf-GAL80/+; Pdf-GAL80/+*, P<<0.01, **vs** *w; +/+;+/UAS-NaChBac2* , P<<0.01).

[g,h] PDF$^+$CRY$^+$, PDF$^-$ CRY‡, PDF$^-$ CRY*>NaChBac single period flies were not significantly different from controls ([g]ANOVA, $F_{2,65} = 3.36$, P=0.41; [h]ANOVA, $F_{2,65} = 3.33$, P=0.42).

[i,j] PDF$^+$CRY$^+$>Kir2.1 single period flies had slightly shorter activity rhythms than controls. Differences were suggestive for one line and significant for the other (overall [i]ANOVA, $F_{2,80} = 20.87$, P<<0.01; but Bonferroni post-hoc *yw; Pdf-GAL4/+; +/ UAS-Kir2.1* **vs** *yw; Pdf-GAL4/+; +/+*, P<<0.01, **vs** *w; +/+; +/ UAS-Kir2.1* , P=0.15. Overall [j]ANOVA, $F_{2,94} = 4.82$, P=0.01; Bonferroni post-hoc: *yw; Pdf-GAL4/UAS-Kir2.1; +/+* **vs** *yw; Pdf-GAL4/+; +/+*, P=0.04, **vs** *w; +/UAS-Kir2.1;+/+* , P=0.01).

[k] PDF$^+$CRY$^+$, PDF$^-$ CRY‡, PDF$^-$ CRY*>Kir2.1 single period had intermediate periods compared to controls (overall ANOVA, $F_{2,70} = 7.68$, P<<0.01; but Bonferroni post-hoc: *w; +/ +; cry*$_{13}$*-GAL4/ UAS-Kir2.1* **vs** *yw; +/+; cry*$_{13}$*-GAL4/+*, P=0.43, **vs** *w; +/+; +/ UAS-Kir2.1*, P=0.03).

[l] PDF^{+}CRY^{+}>HID, RPR single period flies had shorter activity rhythms than controls (overall ANOVA, $F_{2,98} = 43.37$, P<<0.01; Bonferroni post-hoc *yw, UAS-hid, UAS-rpr; Pdf-GAL4/+; +/+* ***vs*** *yw; Pdf-GAL4/+; +/+*, P<<0.01, ***vs*** *yw, UAS-hid, UAS-rpr*; *+/+; +/+*, P<<0.01).

[m] PDF^{-} CRY*, PDF^{-}CRY^{-}> HID, RPR single period flies showed intermediate periods compared to controls (overall ANOVA, $F_{2,103} = 21.35$, P<<0.01; but Bonferroni post-hoc: *yw, UAS-hid, UAS-rpr; tim-GAL4/+; cry-GAL80*$_{2e3m}$ */+* ***vs*** *w; tim-GAL4/+; cry-GAL80*$_{2e3m}$*/+*, P=0.05, ***vs*** *yw, UAS-hid, UAS-rpr*; *+/+; +/+*, P=1.00).

[n,o] PDF^{+}CRY^{+}>CLKΔ single period flies had shorter activity rhythms than controls (overall [n]ANOVA, $F_{2,102} = 9.36$, P<<0.01; Bonferroni post-hoc *yw; Pdf-GAL4/ UAS-ClkΔ1;+/+* ***vs*** *yw; Pdf-GAL4/+; +/+*, P<<0.01, ***vs*** *w; +/UAS-ClkΔ1;+/+*, P<<0.01. Overall [o]ANOVA, $F_{2,86} = 31.10$, P<<0.01; Bonferroni post-hoc *yw; Pdf-GAL4/ UAS-ClkΔ2;+/+* ***vs*** *yw; Pdf-GAL4/+; +/+*, P<<0.01, ***vs*** *w; +/UAS-ClkΔ2;+/+*, P<<0.01).

[p,q] PDF^{+}CRY^{+}, PDF^{-} CRY‡, PDF^{-} CRY*> CLKΔ single period flies were not significantly different from controls (overall [p]ANOVA, $F_{2,83} = 3.50$, P=0.04; but Bonferroni post-hoc: *w; +/ UAS-ClkΔ1; cry*$_{13}$*-GAL4/+* ***vs*** *yw; +/+; cry*$_{13}$*-GAL4/+*, P=0.03, ***vs*** *w; +/UAS-ClkΔ1;+/+*, P=0.40. Overall [q]ANOVA, $F_{2,89} = 12.91$, P<<0.01; but Bonferroni post-hoc: *w; +/ UAS-ClkΔ2; cry*$_{13}$*-GAL4/+* ***vs*** *yw; +/+; cry*$_{13}$*-GAL4/+*, P<<0.01, ***vs*** *w; +/UAS-ClkΔ2;+/+*, P=0.54).

[r] PDF^{-} CRY‡, PDF^{-} CRY*> CLKΔ single period flies were not significantly different from controls (overall ANOVA, $F_{3,99} = 3.97$, P=0.01; but Bonferroni post-hoc: *w; Pdf-GAL80*$_{96a}$ */ UAS-ClkΔ1; cry*$_{13}$*-GAL4/+* ***vs*** *w; yw; +/+; cry*$_{13}$*-GAL4/+*, P=1.00, ***vs*** *w; +/UAS-ClkΔ1;+/+*, P=0.40, ***vs*** *w, UAS-cryΔ14.6; Pdf-GAL80*$_{96a}$*/+; +/+*, P=1.00)

Table S3. Locomotor activity rhythms after overexpression of CRYΔ in DN1p neurons. Related to Figure 4.

Genotype	Over-expressing	N (n)	τ ± S.E.M.	SR(%)	CR(%)	AR(%)	Neurons
w, UAS-cryΔ14.6; +/+; CLK4.1M-GAL4/ +	CRYΔ	114 (2)	23.64 ± 0.03	94	5	1	several DN1p
w, UAS-cryΔ14.6; +/+; CLK4.5F-GAL4/ +	CRYΔ	50 (2)	23.43 ± 0.04	94	4	2	several DN1p
w, UAS-cryΔ14.6; +/+; +/+		46 (4)	23.78 ± 0.06	76	15	9	Controls
w; +/+; CLK4.5F-GAL4/+		32 (1)	23.50 ± 0.04	97	0	3	Controls

The period of locomotor activity was determined by Fourier (spectral) analysis [S4]. Only male flies were studied. **N** = total number of flies examined; n = number of independent replicates. **τ ± S.E.M.**, average period of locomotor activity ± standard error of the mean of flies showing a single period. **SR(%)**, percentage of flies showing a single period. **CR(%)**, percentage of flies showing complex rhythms. **AR(%)**, percentage of arrhythmic flies.

ANOVA, Genotypes, $F_{3,216}$=10.979, P<<0.001. Bonferroni post-hoc comparisons: the genotype *w, UAS-cryΔ14.6; +/+; CLK4.1M-GAL4/* + was not significantly different from the parental control *w, UAS-cryΔ14.6; +/+; +/+* (P=0.088). The genotype *w, UAS-cryΔ14.6; +/+; CLK4.5F-GAL4/* + was not significantly different from the parental control *w; +/+; CLK4.5F-GAL4/+* (P=1). Therefore, driving CRYΔ in several DN1p does not result in a shorter period.

Table S4. Locomotor activity rhythms after developmental stage-specific manipulation of clock neurons. Related to Figure 5.

**Genotype**	**Condition**	**Over-expressing**	**N (n)**	**τ ± S.E.M.**	**SR(%)**	**CR(%)**	**AR(%)**	**Neurons**
w, UAS-cryΔ14.6; UAS-CD8GFP/+; Pdf-GS/+	DO		78 (2)	23.69 ± 0.05[a]	97	0	3	$PDF^{+}CRY^{+}$
w, UAS-cryΔ14.6; UAS-CD8GFP/+; Pdf-GS/+	DA	$CRY\Delta_{A}$	64 (2)	24.01 ± 0.07[a]	92	5	3	
w, UAS-cryΔ14.6; UAS-CD8GFP/+; Pdf-GS/+	DC	$CRY\Delta_{Ch}$	26 (1)	24.22 ± 0.10[a]	100	0	0	
yw; Pdf-GAL4/+; +/UAS-dTrpA1	18 ºC	$dTRPA1_{C}$	20 (1)	23.93 ± 0.07[b]	85	0	15	
yw; Pdf-GAL4/+; +/UAS-dTrpA1	28 ºC	$dTRPA1_{O}$	24 (1)	24.40 ± 0.18[b]	62	21	17	
yw; tim-GAL4/+; cry-GAL80$_{2e3m}$*/ UAS-dTrpA1*	18 ºC	$dTRPA1_{C}$	14 (1)	24.11 ± 0.51[c]	72	7	21	$PDF^{-}CRY^{*}$, $PDF^{-}CRY^{-}$
yw; tim-GAL4/+; cry-GAL80$_{2e3m}$*/ UAS-dTrpA1*	28 ºC	$dTRPA1_{O}$	16 (1)	22.66 ± 0.26[c]	75	19	6	
w; UAS-CD8GFP/+; Pdf-GS/+	DO		81 (3)	23.89 ± 0.05[a]	91	4	5	Controls
w; UAS-CD8GFP/+; Pdf-GS/+	DA		65 (2)	24.08 ± 0.05[a]	91	6	3	
w; UAS-CD8GFP/+; Pdf-GS/+	DC		33 (1)	24.01 ± 0.08[a]	91	3	6	
w, UAS-cryΔ14.6; +/+; +/+	DO		93 (2)	23.89 ± 0.07[a]	70	4	26	
w, UAS-cryΔ14.6; +/+; +/+	DA		80 (2)	24.01 ± 0.16[a]	70	5	25	
w, UAS-cryΔ14.6; +/+; +/+	DC		58 (1)	23.86 ± 0.10[a]	71	10	19	
yw; Pdf-GAL4/+; +/+	18 ºC		22 (1)	23.88 ± 0.07[b]	100	0	0	
yw; Pdf-GAL4/+; +/+	28 ºC		28 (1)	24.06 ± 0.06[b]	100	0	0	
w; tim-GAL4/+; cry-GAL80$_{2e3m}$*/+*	18 ºC		24 (1)	23.88 ± 0.31[c]	38	0	62	
w; tim-GAL4/+; cry-GAL80$_{2e3m}$*/+*	28 ºC		32 (1)	23.76 ± 0.09[c]	69	13	18	
w; +/+; +/ UAS-dTrpA1	18 ºC		11 (1)	22.94± 0.18[b, c]	91	0	9	
w; +/+; +/ UAS-dTrpA1	28 ºC		19 (1)	23.09± 0.11[b, c]	95	0	5	

The period of locomotor activity was determined by Fourier (spectral) analysis [S4]. Only male flies were studied. **N** = total number of flies examined; n = number of independent replicates. **τ ± S.E.M.**, average period of locomotor activity ± standard error of the mean of flies showing a single period. **SR(%)**, percentage of flies showing a single period. **CR(%)**, percentage of flies showing complex rhythms. **AR(%)**, percentage of arrhythmic flies. Experiments carried out with the

temperature activated cation channel dTRPA1 were conducted at 18 ºC and 28 ºC to maintain the channel in the close ($dTRPA1_C$) or open ($dTRPA1_O$) conformation, respectively. All other experiments were conducted at 25 ºC. The *geneswitch* (*GS*) system requires the drug RU486 to be activated into a functional GAL4. Experiments using *GS* were conducted in the absence of drug (DO, drug omitted), exposing the adults only to the drug (DA, drug acute treatment), exposing individuals to the drug since the earliest developmental stages (DC, drug chronic treatment).

[a] ANOVA, Genotype, $F_{2,477}$=0.554, P=0.575, Treatment, $F_{2,477}$=7.218, P=0.001, Genotype*Treatment, $F_{4,477}$=2.529, P=0.04. Bonferroni post-hoc comparisons, DO *vs*. DA, P=0.001, DO *vs*. DC. P=0.037, DA *vs*. DC, P=1.00.

[b] ANOVA, Genotype, $F_{2,104}$=57.694, P<0.001, Temperature, $F_{1,104}$=9.349, P=0.003, Genotype*Temperature, $F_{2,104}$=1.452, P=0.239.

[c] ANOVA, Genotype, $F_{2,75}$=6.219, P=0.003, Temperature, $F_{1,75}$=6.200, P=0.015, Genotype*Temperature, $F_{2,75}$=6.322, P=0.003.

Table S5. Locomotor activity rhythms after overexpression of SGG and CRY. Related to Figure 6.

Genotype	Over-expressing	N (n)	τ ± S.E.M.	SR(%)	CR(%)	AR(%)	Neurons
w, UAS-sgg; tim-GAL4/+; cry-GAL80$_{2e3m}$/ +	SGG	78 (5)	23.57 ± 0.08	65	35	0	PDF$^-$CRY^{+*}, PDF$^-$CRY$^-$
w; tim-GAL4/+; cry-GAL80$_{2e3m}$/ UAS-HAcry16.1	CRY	29 (2)	23.72 ± 0.18	72	21	7	
w, UAS-sgg; tim-GAL4/+; cry-GAL80$_{2e3m}$/ UAS-HAcry16.1	SGG, CRY	46 (3)	22.79 ± 0.13	74	13	13	
w; tim-GAL4/+; cry-GAL80$_{2e3m}$/+		98 (8)	23.50 ± 0.05	75	10	15	Controls
w, UAS-sgg; +/+; +/+		47 (4)	23.75 ± 0.05	96	4	0	
w; +/+; +/ UAS-HAcry16.1		16 (1)	23.90 ± 0.06	88	12	0	

The period of locomotor activity was determined by Fourier (spectral) analysis [S4]. Only male flies were studied. **N** = total number of flies examined; n = number of independent replicates. **τ ± S.E.M.**, average period of locomotor activity ± standard error of the mean of flies showing a single period. **SR(%)**, percentage of flies showing a single period. **CR(%)**, percentage of flies showing complex rhythms. **AR(%)**, percentage of arrhythmic flies. *HAcry16.1* carries at its 5' the sequence for production of the HA epitope tag.

ANOVA, Genotypes, $F_{5,232}$=15.909, P<<0.001. Bonferroni post-hoc comparisons, the genotype *w, UAS-sgg; tim-GAL4/+; cry-GAL80$_{2e3m}$/ UAS-hacry16.1/+* resulted significantly different in all pairwise comparisons with the other genotypes (P<<0.001). The comparisons between all other genotypes were not significant.

Supplemental Experimental Procedures

Fly strains and maintenance.

Flies were raised at 25 °C on standard yeast-corn-sucrose-agar medium under LD 12:12. All strains employed have been previously described, as follows. Reporter (UAS-) lines, *CRY*Δ and *CRY* [5], *NachBac* [19] (obtained from the Bloomington Stock centre), *Kir2*.1 [24] (obtained from K Moffat), *CLK*Δ [28], *HID,RPR* [26], *SGG* (constitutively active form) [S5], RFP^{NLS} (aka *RedStinger*), *GFP* and *TRPA1* (Bloomington Stock centre). Driver lines were as described in [14], with the exception of cry_{39}*-GAL4* reported in [S6], *tim-GAL4, Pdf-GAL80; Pdf-GAL80* reported in [S7], *Pdf-GeneSwitch* reported in [30] and *Pdf-GAVPO* (this publication, see below).

Behavioural analyses and statistics

Locomotor activity was recorded (males only) with DAM2 Trikinetics monitors for 3-5 days in LD 12:12 and 7-12 days in DD, at constant 25 °C. Activity periods were determined using the CLEAN package [S4]. Briefly, flies were considered rhythmic in the circadian range if showing a (spectral analysis) harmonic component (which also defines the period of the fly) in the interval 12 h< > 30 h, such that it exceeded the 99% confidence limit calculated by a Monte Carlo simulation (single rhythm flies, SR). Flies were considered showing complex rhythms (CR) when two or more significant (above the 99% confidence limit) harmonic components in the circadian range where present in the same individual. Arrhythmic (AR) animals did not show significant harmonics in the circadian range. When required we calculated the Power of a rhythm as a measure of its robustness. Based on the empirical observation that the more robust the rhythm the higher the peak value (P) of the harmonic component compared to the 99% confidence limit (CL_{99}), we defined Power as $Pw = [(P-CL_{99})/CL_{99} * 100]$.

Statistical analyses were carried out using PASW Statistics 18 (Release 18.0.2).

Immunohistochemistry

For each experiment, flies were processed in parallel. Flies were first fixed at room temperature (as for the whole procedure except incubation with primary antibodies) on a rotating wheel for 2 h with 4% formaldehyde, 0.1% Triton-X in PBS. Then they were washed for 15 min in PBS, and finally brains were dissected in PBS. Brains were permeabilised 3 x 20 min with PBS, 1% Triton-X. Blocking was performed in PBS, 0.5% Triton-X, 10% goat serum for 1 h. Primary antibodies were diluted in fresh PBS, 0.5% Triton-X, 10% goat serum and incubated for 72 h at 4 °C. Unbound antibodies were washed 3 x 20 min with PBS, 0.5% Triton-X. Secondary fluorescent antibodies were diluted in PBS, 0.5% Triton-X and incubated (in darkness) for 3 h. After washes (3x 20 min, PBS, 0.5% Triton-X) brains were rinsed in distilled water and mounted in 20% PBS (pH = 8.5), 80% Glycerol + 3% n-propylgallate (Sigma). Primary antibodies were as follows. Mouse anti-PDF (1:50, DSHB), rabbit anti-PDP1ε (1:5000, gift of Justin Blau), rabbit anti-GFP (1:1000, Invitrogen), mouse anti-GFP (1:2000, BAbCo), rabbit anti-CRY (1:500, see below). Fluorescent secondary antibodies were as follows. Goat anti-rabbit IgG-Cy3 (1:200, Jackson), goat anti-mouse IgG-Cy2 (1:200, Jackson), goat anti-rabbit IgG-AlexaFluor647 (Jackson, 1:200), goat anti-rabbit IgG-Biotin (1: 600, Jackson), Streptavidin Dylight-649 (1:600, Jackson).

Optical sections were imaged on a Leica TCS SP5 confocal microscope (Figure 4) or with an Olympus FV1000 (Figure S1) confocal microscope.

Images were analysed with ImageJ software to quantify the mean pixel intensity (mpi) of cytoplasm, nucleus and background by an experimenter that was blind to the identity of the sample. Staining Index (SI) was calculated with the following formula:

[(Cell$_{mpi}$ – background$_{mpi}$)/background$_{mpi}$]x(number of cells observed/maximum number of cells).

For each hemisphere we then calculated an average for each cell type. These were the values used for statistical analyses. For the different types of neurons we used the following as the maximum number of cells, s-LNvs=4; l-LNvs=4; LNds=6; DN2=2; DN1s=16; DN3s=40. All cells were quantified individually with the exception of the DN3s that were quantified as a group.

Production of anti-CRY antibody.

We amplified the coding sequence of *cry* as three overlapping fragments:

f1 (from 1 to 600 bp. Primers: F, 5’-GGCAT**ATG**GCCACGCGAGGGGCGAATG-3’;

R, 5’-GGGGATCCTTACTCGAACAACTTAAGACTTCGGCAG-3’),

f2 (540-1296 bp. Primers: F, 5’-CGCAT**ATG**GAAGACGCCACCTTTGTCGAGC-3’;

R, 5’-GGGGATCCTTACAGCAGCCTTTCAAACGCCGAG-3’) and

f3 (999-1626 bp. Primers: F, 5’-GGCAT**ATG**AATGACATCTGCCTGAGCATCCCG-3’;

R, 5’-GGGGATCCTTAAACCACCACGTCGGCCAGCCAG-3’).

All F primers carried a 5’ *Nhe*I site and a start codon (in bold); all R primers carried a 5’ *Xho*I site and a stop codon (underlined). After amplification the three products were cloned as *Nhe*I-*Xho*I fragments into pET14b (Merck Millipore) and were verified by Sanger sequencing. We expressed the three His6x-fusion proteins in *E. coli* BL21(DE3)pLysS strain (Merck Millipore) and we purified them by chromatography on Ni^{+}-resin as insoluble products. Denaturants were removed by dialysis and the proteins were resuspended in PBS at a final concentration of 0.4 mg/ml. The three fragments mixed together were injected in rabbits by a commercial service (Polypeptide Laboratories).

Cloning of Pdf-GAVPO

We amplified *GAVPO* [S3] by PCR (from the first ATG to the HSP70 terminator sequence) using PHUSION DNA polymerase (all enzymes were from New England Biolabs) and the following primers:

Geneart_GAVPO_F$_{KOZAK}$:

5'-TACCAAACGGTACCGCTAGCCAAAAC**ATG**AAGCTACTGTCTTCTATCGAA-3'

(underlined: KOZAK sequence, bold: first codon) and

Geneart_GAVPO_R:

5'-TCGATAAGCTTGTTTAAACTTACTTGTCATCATCGTCTTTGTAG-3'.

The product was cloned into *LexAP65* (Addgene), previously digested with *Nhe*I and *Pme*I, using the GeneArt® Seamless Cloning and Assembly kit (Life Technologies). To increase mRNA stability we replaced *Hsp70* with the *SV40* terminator sequence. We amplified the latter from *pJFRC19* [S8] with primers

*Pme*I_SV40_F: 5'-ACTGGTTTAAACTAAGGTAAATATAAAATTTTTAAGTGTAT-3'

and *Xba*I_SV40_R: 5'-CAGTTCTAGAAGATCGATCCAGACATGATAA-3'

and cloned it into *pCR®-Blunt* (Life Technologies). Then we isolated the *SV40* sequence by *Pme*I and *Xba*I digestion and we inserted it (by T4 ligation) in exchange to the *Hsp70* equally restricted. Finally, we introduced the *Pdf* promoter sequence upstream to the *GAVPO* coding region. We amplified the promoter sequence from genomic DNA with primers:

PDF_pr_F: 5'-GCCCGGGCTCCGTGGGTTTCATCCTTACCA-3' and

Kpn_PDF_pr_R: 5'-GGTACCCAGGAGACTTGCGAATGAACGT-3'.

We cloned it into *pCR®-Blunt* and then we excised it by *Kpn*I and *Eco*53kI restriction. This fragment was ligated upstream of the GAVPO coding sequence into *Kpn*I and *Nae*I (compatible with *Eco*53kI) sites resulting in *Pdf-GAVPO*. The accuracy of the construct was

verified by Sanger sequencing. Transgenic flies were produced by a commercial service (Cambridge University) using PhiC31-recombinase. We chose the *attP40* site for integration.

Supplemental references

S1. Yasuyama, K., and Meinertzhagen, I.A. (2010). Synaptic connections of PDF-immunoreactive lateral neurons projecting to the dorsal protocerebrum of *Drosophila melanogaster*. J Comp Neurol. 518, 292-304.

S2. Choi, C., Cao, G., Tanenhaus, A.K., McCarthy, E.V., Jung, M., Schleyer, W., Shang, Y., Rosbash, M., Yin, J.C., and Nitabach MN. (2012). Autoreceptor control of peptide/neurotransmitter corelease from PDF neurons determines allocation of circadian activity in *Drosophila*. Cell Rep. 2, 332-344.

S3. Wang, X., Chen, X. and Yang, Y. (2012) Spatiotemporal control of gene expression by a light-switchable transgene system. Nature Methods 9, 266-269.

S4. Rosato, E., and Kyriacou, C.P. (2006). Analysis of locomotor activity rhythms in Drosophila. Nat Protoc 1, 559-68.

S5. Martinek, S., Inonog, S., Manoukian, A.S., and Young, M.W. (2001). A role for the segment polarity gene *shaggy*/*GSK*-3 in the *Drosophila* circadian clock. Cell 105, 769-779.

S6. Klarsfeld, A., Malpel, S., Michard-Vanhée, C., Picot, M., Chélot, E. and Rouyer F. (2004) Novel features of cryptochrome-mediated photoreception in the brain circadian clock of *Drosophila*. J. Neurosci. 24, 1468–1477

S7. Murad, A., Emery-Le, M., and Emery, P. (2007). A subset of dorsal neurons modulates circadian behavior and light responses in Drosophila. Neuron 53, 689-701.

S8. Pfeiffer, B.D., Ngo, T.T., Hibbard. K.L, Murphy, C., Jenett, A., Truman, J.W. and Rubin, (2010) Refinement of tools for targeted gene expression in Drosophila. Genetics 186, 735-755.
