# Supplementary material for: The Logic of Circadian Organization in Drosophila
Source: Curr Biol. 2014 Oct 6;24(19):2257–66. doi: 10.1016/j.cub.2014.08.023 (PMC4188814; doi:10.1016/j.cub.2014.08.023)
Supplement: Document S2. Article plus Supplemental Information [file mmc2.pdf]

Current Biology 24, 2257–2266, October 6, 2014 ©2014 The Authors http://dx.doi.org/10.1016/j.cub.2014.08.023

Article

The Logic of Circadian Organization in *Drosophila*

Stephane Dissel,[1,2,3] Celia N. Hansen,[1,2] Özge Özkaya,[1] Matthew Hemsley,[1] Charalambos P. Kyriacou,[1] and Ezio Rosato[1,*]
[1]Department of Genetics, University of Leicester, Leicester LE1 7RH, UK

Summary

Background: In the fruit fly *Drosophila melanogaster*, interlocked negative transcription/translation feedback loops provide the core of the circadian clock that generates rhythmic phenotypes. Although the current molecular model portrays the oscillator as cell autonomous, cross-talk among clock neurons is essential for robust cycling behavior. Nevertheless, the functional organization of the neuronal network remains obscure.
Results: Here we show that shortening or lengthening of the circadian period of locomotor activity can be obtained either by targeting different groups of clock cells with the same genetic manipulation or by challenging the same group of cells with activators and repressors of neuronal excitability.
Conclusions: Based on these observations we interpret circadian rhythmicity as an emerging property of the circadian network and we propose an initial model for its architectural design.

Introduction

The circadian clock provides the interface between an organism and its 24 hr geophysical environment. As currently accepted, the fly clock is constituted by interlocked negative transcription/translation feedback loops (TTFLs). At the core of the system are the activators CLOCK (CLK) and CYCLE (CYC) that bind to the promoters of the *period* (*per*) and *timeless* (*tim*) genes, initiating their transcription. After translation, the negative autoregulators PER and TIM become the substrate of several kinases and phosphatases, they dimerize, translocate into the nucleus, and repress the CLK/CYC complex. The second feedback loop is centered around *Clk* and involves the rhythmic expression of *PAR domain protein 1* (*Pdp1*) and *vrille* (*vri*) (reviewed in [1]). The blue-light-sensitive protein CRYPTOCHROME (CRY) regulates photo-responsiveness and flies with altered CRY function display aberrant circadian light entrainment [2–5] and visual behavior [6]. Moreover, immunofluorescence (IF) and confocal microscopy reveal that α-CRY immunoreactivity (IR) is often found at the level of neuronal projections [7], suggesting that CRY may play additional roles. Null mutants for *cry* (*cry*^{0}) show defects in rhythmic behavior under constant light (LL) [8], so it is possible that CRY exerts direct functions in central clock neurons. Indeed, light-activated CRY increases neuronal firing via an unknown mechanism [9].

Clock neurons constitute a network of cells expressing clock genes that are divided into lateral neurons (six dorsal lateral neurons [LNds], four large ventral lateral neurons [l-LNvs], four small ventral lateral neurons [s-LNvs], a single *Pdf*-null ventral lateral neuron [pn-LNv], and three lateral posterior neurons [LPNs]) and dorsal neurons (~16 DN1s, 2 DN2s, and ~40 DN3s) (reviewed in [10]). Although the classic molecular model portrays the clock as cell autonomous, cellular cross-talk appears to be essential for its function [11–14].

The circadian neurons can be further grouped or differentiated by the activation of promoters of clock and clock-related genes. For instance, all clock neurons express *tim* [15] whereas the *Pigment-dispersing factor* (*Pdf*) promoter is active only in the s- and l-LNvs [16]. A *cry* 5.5-kb promoter [17] is seemingly expressed in all LNvs, in the LNds, and in two DN1s [11] although Shafer et al. [18] reported further expression in two additional DN1s (called DN1a) and two DN3s. By combining the expression of these promoters, coupled to either *GAL4* (to drive transcription of a reporter gene) or *GAL80* (to inhibit GAL4 function), it is possible to define subsets of clock cells and to manipulate them selectively by expressing genes whose products alter the electrical properties of the neurons or the running of the clock.

In our study we have introduced local alterations in the neuronal network and have investigated the period of locomotor activity under constant darkness and temperature (DD), an artificial condition where the interactions among neurons is independent from the light-dark (LD) cycle. We did this by initially distinguishing among clock cells based on whether they express both the *Pdf* and *cry* promoters (PDF$^+$CRY$^+$), the *cry* promoter only (PDF$^-$CRY$^+$), or neither (PDF$^-$CRY$^-$). Our results suggest a model for the logic that regulates the neuronal network under these conditions.

Results and Discussion

CRYΔ Lengthens or Shortens the Endogenous Period of Locomotor Activity when Expressed in Different Clock Neurons

CRYΔ, a C-terminal deletion of CRY, renders CRY constitutively active, as revealed in a number of molecular (light-independent binding to PER and TIM) and behavioral (long period of locomotor activity in DD) phenotypes [4, 5]. Based on the discovery that light-activated CRY increases neuronal firing [9], we presumed that CRYΔ could activate neurons and enhance their output in DD, which might be reflected in period changes. We used a *tim-GAL4* driver and several *UAS-cryΔ* lines and confirmed that overexpression of CRYΔ in all clock cells, namely PDF$^+$CRY$^+$ ∩ PDF$^-$CRY$^+$ ∩ PDF$^-$CRY$^-$, results in ~1 hr lengthening of the endogenous period of locomotor activity compared to controls [5] (Table S1 available online). We noticed that the vast majority of CRYΔ flies had simple rhythmicity (SR) although a few showed complex rhythms (CR, more than one periodicity in a single fly) or arrhythmicity (AR) but in proportions no different from controls.

We asked whether all clock cells contribute to the free running period or whether one group of neurons imposes its

[2]Co-first author
[3]Present address: Department of Anatomy and Neurobiology, Washington University in St Louis, 660 South Euclid Avenue, St Louis, MO 63110, USA
*Correspondence: er6@le.ac.uk
This is an open access article under the CC BY license (http://creativecommons.org/licenses/by/3.0/).

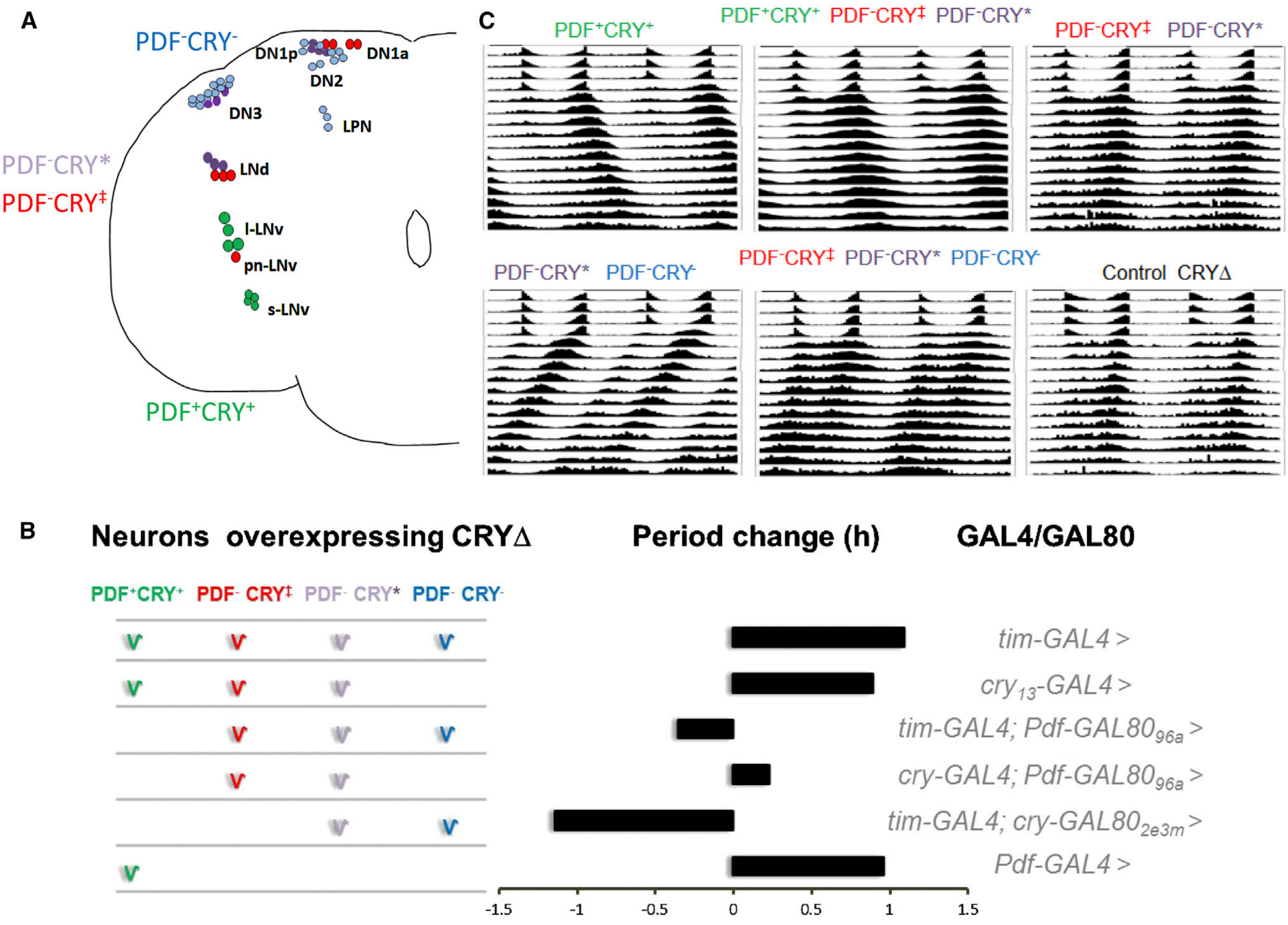

Figure 1. Different Groups of Neurons Affect Rhythmic Behavior

(A) Operational classification of clock neurons according to the expression of the *Pdf* and *cry* promoters (see also text and Figure S1).

(B) Period differences between CRYΔ-expressing and control flies. Horizontal black bars refer to the period change (in hours) compared to controls when CRYΔ (*UAS-cryΔ14.6*) is expressed in particular groups of neurons (shown on the left) as a result of different GAL4/GAL80 combinations (shown on the right).

(C) Average locomotor activity profiles of CRYΔ-expressing flies showing 4 days in LD 12:12 and 12 days in DD. Genotypes and statistics are as reported in Table S1; control CRYΔ was *w*, *UAS-cryΔ14.6*.

own rhythmicity to behavior [13]. We focused on one line (*UAS-cryΔ14.6*), which will hereafter be referred to as *cryΔ*.

PDF$^+$CRY$^+$ > CRYΔ (*Pdf-GAL4* > *UAS-cryΔ*) and PDF$^+$ CRY$^+$ ∩ PDF$^-$CRY$^+$ > CRYΔ (*cry*$_{13}$*-GAL4* > *UAS-cryΔ*) flies both revealed a ~1 hr longer period compared to controls. We obtained a similar value for the PDF$^+$CRY$^+$ ∩ PDF$^-$ CRY$^+$ ∩ PDF$^-$CRY$^-$ > CRYΔ genotype (*tim-GAL4* > *UAS-cryΔ*), which argues against the assumption that these three drivers largely differ in their "strength" [13] (Figure 1, Table S1). Then, we expressed CRYΔ in smaller groups of neurons by combining the GAL80 repressor with GAL4 drivers. We used the lines *cry-GAL80*$_{2e3m}$ and *Pdf-GAL80*$_{96a}$, each carrying two copies of GAL80, that have been reported to repress UAS-dependent expression in the PDF$^+$CRY$^+$ ∩ PDF$^-$CRY$^+$ and the PDF$^+$CRY$^+$ cells, respectively [11]. To confirm the extent of GAL80-mediated inhibition of GAL4 activity, we measured anti-GFP IR of PDF$^+$CRY$^+$ cells in *tim-GAL4* > *UAS-GFP*, in *tim-GAL4* ∩ *cry-GAL80*$_{2e3m}$ > *UAS-GFP*, and in *tim-GAL4* ∩ *Pdf-GAL80*$_{96a}$ > *UAS-GFP* flies. For both GAL80 configurations, we were able to confirm a good and equivalent level of repression of GAL4 activity in PDF$^+$CRY$^+$ cells (Figures S1A and S1B). However, in all *tim-GAL4* ∩ *cry-GAL80*$_{2e3m}$ > *UAS-GFP* preparations, we observed a robust anti-GFP signal in three LNds, meaning that GAL80 cannot efficiently inhibit GAL4 in these cells because of lower *cry* expression (Figures S1A and S1C). GAL80-mediated repression of GAL4 was inefficient also in four DN1ps and in three or four *cry*-positive DN3s. Conversely, good repression was achieved in the DN1as, in two DN1ps, and in the pn-LNv (Figure S1C and data not shown). Thus, the PDF$^-$CRY$^+$ group is heterogeneous and should be divided into two. Henceforth we identify cells that strongly or weakly express *cry* as PDF$^-$CRY‡ and PDF$^-$ CRY*, respectively.

We tested PDF$^-$CRY‡ ∩ PDF$^-$CRY* > CRYΔ (*cry*$_{13}$*-GAL4* ∩ *Pdf-GAL80*$_{96a}$ > *UAS-cryΔ*) and PDF$^-$CRY‡ ∩ PDF$^-$CRY* ∩ PDF$^-$CRY$^-$ > CRYΔ (*tim-GAL4* ∩ *Pdf-GAL80*$_{96a}$ > *UAS-cryΔ*) flies; for both genotypes the free running period of locomotor activity was not significantly different from controls. However, when we analyzed PDF$^-$CRY* ∩ PDF$^-$CRY$^-$ > CRYΔ (*tim-GAL4* ∩ *cry-GAL80*$_{2e3m}$ > *UAS-cryΔ*) flies, we observed a significant 1.2 hr shortening of the period (Figure 1; Table S1). This result cannot be explained in terms of residual GAL4 expression in the PDF$^+$CRY$^+$ cells as we detected similar levels of IR in them when driving GFP in both *tim-GAL4* ∩ *Pdf-GAL80*$_{96a}$

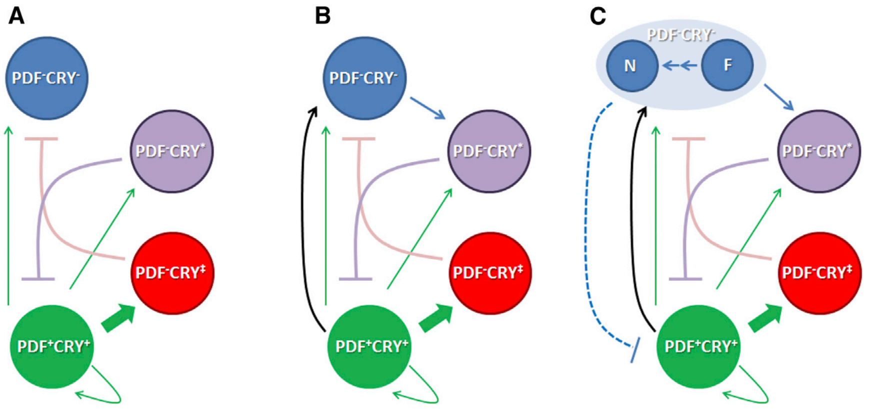

Figure 2. Circadian Logic

We operationally divided the circadian neurons into PDF$^+$CRY$^+$, PDF$^-$CRY‡, PDF$^-$CRY*, and PDF$^-$CRY$^-$ groups (see also Figures 1 and S1).

(A) The PDF$^+$CRY$^+$ cells have a large influence on the network as they can communicate with a significant number of neurons through PDF (green arrows). Probably, the PDF$^-$CRY‡ neurons are particularly responsive to PDF signaling (thick green arrow) as they express the PDF receptor (PDFR) at the highest level [37]. Activation of the PDF$^+$CRY$^+$ cells results first in a longer period of locomotor activity and then, as activation increases, in complex rhythms (Figure S3). Activation of the PDF$^-$CRY* ∩ PDF$^-$CRY$^-$ cells results in a shorter period of locomotor activity and then in arrhythmicity. The PDF$^-$CRY‡ and the PDF$^-$CRY* cells have an inhibitory role toward the output of the PDF$^+$CRY$^+$ and the PDF$^-$CRY$^-$ neurons, respectively, thus balancing slower and faster components in the network. This could provide a simple mechanism to achieve phase changes under different environmental conditions.

(B) The PDF$^+$CRY$^+$ also use PDF-independent connections (black arrow) to promote the activity of the PDF$^-$CRY$^-$ cells (see also Figure S2). The latter exert a negative feedback on the former through activation of the PDF$^-$CRY* neurons. Thus, activation of PDF$^+$CRY$^+$ results in direct (PDF-independent) activation and in indirect (mediated and amplified by PDF and the PDF$^-$CRY‡ neurons) repression of the PDF$^-$CRY$^-$ cells. These integrate both pathways and feedback to regulate the activity of the PDF$^+$CRY$^+$ neurons. This could explain how the PDF$^+$CRY$^+$ and PDF$^-$CRY$^-$ groups synchronize together.

(C) Within the PDF$^-$CRY$^-$ group, the N neurons (including the majority of DN1s) are involved in stabilizing behavioral rhythms through synchronization with the PDF$^+$CRY$^+$, which suggests they might have an independent connection with those cells (broken blue line). The F neurons (which include the DN2s and perhaps unrecognized PDF$^-$CRY$^-$ neurons) have an intrinsically faster molecular rhythm. We assume that the signal to inhibit the PDF$^+$CRY$^+$ group can be passed on to the PDF$^-$CRY* before the PDF$^-$CRY‡ cells have time to exert their repression. This would explain how the fast PDF$^-$CRY$^-$ counteract the slower cycling of the PDF$^+$CRY$^+$ neurons, resulting in a 24 hr period. However, the signal for a faster rhythm would not usually reach the N neurons before the repression from the PDF$^-$CRY‡ cells takes effect, suggesting a delay in the connection between the F and N groups of PDF$^-$CRY$^-$ neurons. The activation of the PDF$^-$CRY* and PDF$^-$CRY$^-$ groups (or a reduction in the activation of the PDF$^-$CRY‡ cells) would overcome that delay, causing the N cells (such as the DN1s) to cycle in synchrony with the F cells (such as the DN2s); see Figure 4. The N neurons would then pass on the shorter-period signal to the PDF$^+$CRY$^+$ group (such as the s-LNvs, Figure 4), causing a shorter behavioral period.

and *tim-GAL4* ∩ *cry-GAL80$_{2e3m}$* flies (Figures S1A and S1B). CRYΔ overexpression resulted in a faster rhythm only for the latter genotype, thereby unveiling the contribution of the PDF$^-$CRY* and PDF$^-$CRY$^-$ groups to period, which would otherwise be inhibited by the PDF$^-$CRY‡ cells (this is because the two genotypes above differ in period and in the inclusion of the latter group of cells).

Interestingly, in wild-type flies the DN2 cluster of PDF$^-$CRY$^-$ neurons show faster (∼22 hr) endogenous molecular cycling that is not reflected in the ∼24 hr behavioral rhythms [19]. Under seminatural conditions, they show an advance in molecular cycling compared to other clock neurons, also consistent with a faster period [20, 21]. Conversely, it has been reported that by reducing the contribution of the PDF$^+$CRY$^+$ cells to the network, either by eliminating their main signaling molecule, the neuropeptide PDF [16] (Figure S2), or by reducing their numbers [11] (Table S2), a short activity period is generated. Thus, the overall evidence supports a role for the PDF$^-$CRY$^-$ neurons in generating a short-rhythm phenotype when the balance of the network is tilted in their favor, with the DN2s being a likely but perhaps not exclusive component of this function.

We can rationalize this first set of observations as follows. The overexpression of CRYΔ in PDF$^+$CRY$^+$ cells (*Pdf-GAL4* > *UAS-cryΔ*) results in a longer period. However, these slower-paced cells are not alone in controlling self-sustained behavior because increasing the impact on the network of the PDF$^-$CRY* and PDF$^-$CRY$^-$ neurons (*tim-GAL4* ∩ *cry-GAL80$_{2e3m}$* > *UAS-cryΔ*) results in a shorter period. The effect is reversed by adding PDF$^-$CRY‡ to the ensemble (*tim-GAL4* ∩ *Pdf-GAL80$_{96a}$* > *UAS-cryΔ*), suggesting the latter intervene in a negative control, possibly on the faster PDF$^-$CRY$^-$ neurons (Figure 2A). The simultaneous activation of PDF$^-$CRY‡ and PDF$^-$CRY* neurons (*cry$_{13}$-GAL4* ∩ *Pdf-GAL80$_{96a}$* > *UAS-cryΔ*) had no consequence in terms of period (Figure 1; Table S1). This suggests a balance, with the PDF$^-$CRY‡ neurons inhibiting the faster-paced PDF$^-$CRY$^-$ cells and in turn the PDF$^-$CRY* neurons inhibiting the slower-paced PDF$^+$CRY$^+$ cells (Figure 2A). Note that in this context, the words "activation" and "inhibition" do not have a physiological connotation but are used as logical operators.

Additional Manipulations Consolidate the Model

We wanted to show that the effects observed on period are not specific to CRYΔ but are reproducible by other manipulations. Thus, we employed the same GAL4/GAL80 combinations described above, using effectors that have a known and consistent mode of action independent of the type of neuron.

The overexpression of the depolarization-activated bacterial sodium channel NaChBac [19] using *tim-GAL4* (PDF$^+$CRY$^+$ ∩ PDF$^-$CRY‡ ∩ PDF$^-$CRY* ∩ PDF$^-$CRY$^-$) resulted predominantly in arrhythmicity, confirming the effectiveness of this manipulation (Table S2). Limiting NaChBac expression to the PDF$^+$CRY$^+$ (*Pdf-GAL4>UAS-NaChBac*) neurons generated ∼50% of flies with complex rhythms, showing a major long-period and a minor short-period component. Moreover, those ∼30%–40% individuals with a single activity rhythm had a period 1.5–2.5 hr longer than controls (Figure 3A; Table S2). NaChBac overexpression in PDF$^+$CRY$^+$ induces longer molecular cycles in these cells and directly increases their output, explaining the origin of the major, slower (longer period) activity component [22, 23]. Immunocytochemistry experiments have also indicated that the DN2s show a faster cycle in both controls and NaChBac-overexpressing flies [19]. Thus, it is tempting to speculate that fast PDF$^-$CRY$^-$ neurons (we cannot exclude that neurons in addition to the DN2s might also be

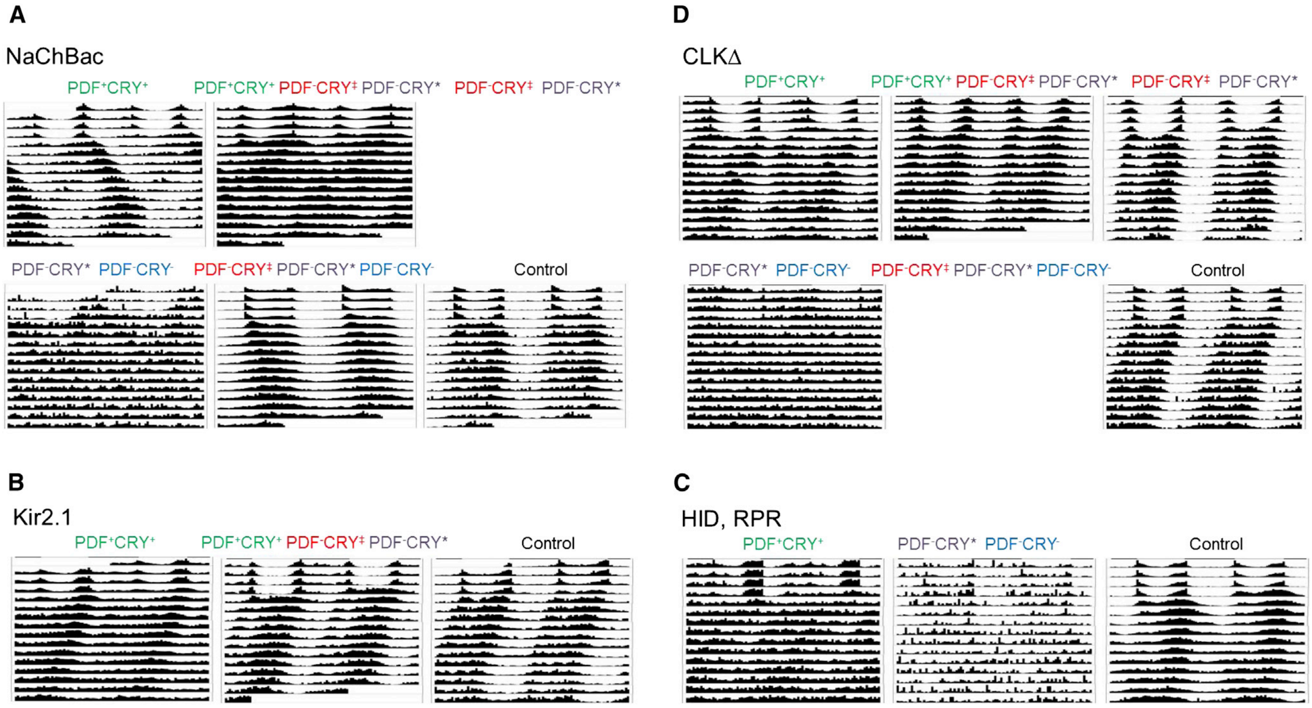

Figure 3. Locomotor Activity Profiles after Manipulation of Clock Neurons

Average locomotor activity profiles of flies showing 3–4 days in LD 12:12 and 12 days in DD. Genotypes and statistics are reported in Table S2.
(A) NaChBac overexpression, line *NaChBac4* is shown. Control: *w; +/ UAS-NaChBac4;+/+*.
(B) Kir2.1 overexpression, line *Kir2.1(III)* is shown. Control: *w; +/+; UAS-Kir2.1/+*.
(C) HID, RPR overexpression. Control: *w, UAS-hid,UAS-rpr; +/+;+/+*.
(D) CLKΔ overexpression, line *CLKΔ1* is shown. Control: *w; UAS-ClkΔ1/+; +/+*.

involved) might be indirectly activated through a positive, PDF-independent connection linking the PDF^{+}CRY^{+} and the PDF^{-}CRY^{-} cells and be responsible for the minor, short activity component detected in this genotype (Figures 2B and S2).

Only 13%–25% of PDF^{-}CRY* ∩ PDF^{-}CRY^{-} > NaChBac (*tim-GAL4* ∩ *cry-GAL80*$_{2e3m}$ > *UAS-NaChBac*) flies showed single rhythms (Figure 3A; Table S2). This result supports the hypothesis that the PDF^{-}CRY* cells might inhibit the PDF^{+}CRY^{+} neurons, explaining why they do not sustain behavioral rhythms in this genotype (Figure 2B). Subsequently we tested PDF^{-}CRY‡ ∩ PDF^{-}CRY* ∩ PDF^{-}CRY^{-} > NaChBac flies (*tim-GAL4* ∩ *2xPdf-GAL80* > *UAS-NaChBac*; Figure 3A; Table S2). Now, about 70%–80% of individuals had a single activity component with a period 0.5–1 hr longer than controls. This implies that the addition of the PDF^{-}CRY‡ group (which is not included in the largely arrhythmic *tim-GAL4* ∩ *cry-GAL80*$_{2e3m}$ > *UAS-NaChBac* genotype) to the ensemble of activated neurons results in the inhibition of the output of the PDF^{-}CRY^{-} cells (because of the longer activity period) and of the PDF^{-}CRY* cells (because the majority of flies are rhythmic). Possibly, the latter inhibition is indirect, in agreement with our previous hypothesis (Figure 2B).

The expression of NaChBac in PDF^{+}CRY^{+} ∩ PDF^{-}CRY‡ ∩ PDF^{-}CRY* (*cry*$_{13}$*-GAL4* > *UAS-NaChBac*) neurons caused a decrease in the number of rhythmic individuals and a shortening in the activity period (now reaching wild-type values for single-rhythm flies) compared to the PDF^{+}CRY^{+} > NaChBac (*PdfGAL4* > *UAS-NaChBac*) genotype (Figure 3A; Table S2). This result is different from the comparison between the same genotypes for CRYΔ flies, which were both rhythmic with a period about 1 hr longer than controls (Figure 1; Table S1). The different strength of the two activators likely accounts for these discrepancies, which in general result from exaggerated effects on period and on rhythmicity following NaChBac overexpression (Figures S3).

We also applied the opposite manipulation, namely the overexpression of the mammalian inward rectifier K^{+} channel KIR2.1, a tool for decreasing membrane excitability in vivo [24, 25]. PDF^{+}CRY^{+} > KIR2.1 (*Pdf-GAL4* > *UAS-kir2.1*) resulted in more than a third of the flies being arrhythmic and in the tendency for a shorter period for those showing a single rhythm. This argues for a disruption in the balance of the network and, in the flies still rhythmic, for a tilt toward the faster PDF^{-}CRY^{-} neurons. Extending the misexpression of KIR2.1 to include also the PDF^{-}CRY‡ and the PDF^{-}CRY* cells (*cry*$_{13}$*-GAL4* > *UAS-kir2.1*) abolished these effects (Figure 3B; Table S2). Finally, we tested the expression of KIR2.1 in PDF^{-}CRY* ∩ PDF^{-}CRY^{-} neurons (*tim-GAL4* ∩ *cry-GAL80*$_{2e3m}$ > *UAS-kir2.1*), but this genotype was not viable.

We then overexpressed the proapoptotic genes *head involution defective* (*hid*) and *reaper* (*rpr*) [26] in PDF^{+}CRY^{+} cells (*Pdf-GAL4* > *UAS-hid, UAS-rpr*) and observed that 50% of the flies were equally either arrhythmic or showed complex rhythms. The remaining half cycled with a period of locomotor activity ~1 hr shorter than controls. Expression of HID and RPR in PDF^{-}CRY* ∩ PDF^{-}CRY^{-} cells (*tim-GAL4* ∩ *cry-GAL80*$_{2e3m}$ > *UAS-hid, UAS-rpr*) resulted in poor viability and ~60% of the surviving flies were arrhythmic, further underscoring the importance of these neurons to the network (Figure 3C; Table S2). The remaining flies showed complex or

single rhythms, the latter having periods on average intermediate to their corresponding controls (Table S2). Although we did not verify this experimentally, it is likely that viable and rhythmic flies must have largely escaped the apoptotic response triggered by HID and RPR. This explanation is in line with the lethality of the *tim-GAL4* ∩ *cry-GAL80$_{2e3m}$* > *UAS-kir2.1* genotype and with previously reported variability in the induction of the apoptotic response in neurons [27].

Driving the production of the dominant-negative CLKΔ mutant [28] in PDF$^-$CRY* ∩ PDF$^-$CRY$^-$ cells (*tim-GAL4* ∩ *cry-GAL80$_{2e3m}$* > *UAS-ClkΔ*) resulted in virtually complete arrhythmicity. Thus, as with NachBac and HID-RPR overexpression, disrupting (independently of mechanism) the PDF$^-$CRY* ∩ PDF$^-$CRY$^-$ groups generated a largely arrhythmic profile, which argues against a simple dominant effect of the PDF$^+$CRY$^+$ cells on the neuronal network (Figure 3D; Table S2). The expression of CLKΔ in PDF$^+$CRY$^+$ cells caused 20%–50% arrhythmicity and 10%–30% of complex rhythms, depending on the line. The remaining 40%–50% of flies showed a single rhythm of activity with a period 0.5–1 hr shorter than controls. This is in line with an increased influence of the PDF$^-$CRY$^-$ cells on the network. By expressing CLKΔ in PDF$^-$CRY‡ ∩ PDF$^-$CRY* cells in addition to the PDF$^+$CRY$^+$ group (*cry$_{13}$-GAL4* > *UAS-ClkΔ*), we increased the ratio of single-rhythm flies to 72%–94% and reverted to a period closer to that of the parental control strains, but slightly shorter (Figure 3D; Table S2). Thus, as previously seen with the overexpression of KIR2.1, we could rescue rhythmicity in flies with weakened PDF$^+$CRY$^+$ neurons by reducing the inhibitory influence of the PDF$^-$CRY* cells on them and by buffering changes in activation of the PDF$^-$CRY‡ cells. Again, this shows that the PDF$^+$CRY$^+$ cells do not act alone in determining circadian rhythmicity and its period. We were unable to obtain viable PDF$^-$CRY‡ ∩ PDF$^-$CRY* ∩ PDF$^-$CRY$^-$ > CLKΔ (*tim-GAL4,* ∩ *Pdf-GAL80$_{96a}$* > *UAS-ClkΔ*) adults but PDF$^-$CRY‡ ∩ PDF$^-$CRY* > CLKΔ (*cry$_{13}$-GAL4,* ∩ *Pdf-GAL80$_{96a}$* > *UAS-ClkΔ*) flies were rhythmic with periods indistinguishable from controls (Figure 3D; Table S2). This result is the same as that observed with CRYΔ, again suggesting that the PDF$^-$CRY‡ and the PDF$^-$CRY* cells inhibit the faster PDF$^-$CRY$^-$ and the slower PDF$^+$CRY$^+$ cycling groups respectively, so the balance of the network is undisturbed (Figure 2B).

Expression of CRYΔ Sets off Nonautonomous Effects on Cellular Clocks

CRYΔ flies with a long behavioral period exhibit a delay in the cycling of clock proteins in PDF$^+$CRY$^+$ neurons [5]. Here we investigated the cellular changes caused by CRYΔ overexpression in PDF$^-$CRY* ∩ PDF$^-$CRY$^-$ (*tim-GAL4* ∩ *cry-GAL80$_{2e3m}$* > *UAS-cryΔ*) and in PDF$^-$CRY‡ ∩ PDF$^-$CRY* ∩ PDF$^-$CRY$^-$ (*tim-GAL4* ∩ *Pdf-GAL80$_{96a}$* > *UAS-cryΔ*) neurons, resulting in short and normal (compensatory) activity rhythms, respectively. We analyzed the cycling of the clock protein PDP1ε in the major classes of clock neurons by IF. Figure 4 shows the staining index (SI) for PDP1 ε at CT0, CT6, CT12, and CT18 (circadian time, CT0 = subjective lights ON, CT12 = subjective lights OFF) during day 2 and 5 in DD.

Control flies (*UAS-cryΔ*) showed, at day 2, maximum SI at about CT18 for all cell types. At day 5 the SI peaked also at CT18 for s-LNvs, LNds, and DN1s, suggesting that these cells cycle with a period of about 24 hr. Instead the SI maximum was reached earlier (about CT12) by the DN2s, suggesting that these neurons run with a shorter period of ∼22 hr. These cells also showed a pronounced reduction in their cycling amplitude compared to the other clusters. The DN3 did not cycle at day 5. Thus, in control flies, s-LNvs, LNds, and DN1s showed molecular oscillations that were compatible with the corresponding behavioral period attributed to them.

The overexpression of CRYΔ in PDF$^-$CRY* ∩ PDF$^-$CRY$^-$ cells (*tim-GAL4* ∩ *cry-GAL80$_{2e3m}$* > *UAS-cryΔ*) targeted directly the DN2s, the large majority of DN1s, the DN3s, and half of the LNds. The DN1s and even more so the DN2s showed a phase advance in PDP1ε immune staining at day 2; at day 5 both groups reached their maximum at around CT6. However, while the DN1s experienced a shortening of their period (in this genotype compared to the control), the DN2s maintained the same faster rhythm seen in wild-type flies. The DN3 did not show significant cycling at day 5. The LNds were considered as a group because we could not distinguish the three PDF$^-$CRY‡ from the three PDF$^-$CRY* cells (we could not discriminate them by anti-CRY staining because both the anti-CRY and the anti-PDP1ε antibodies available to us are made in rabbit), which limits our interpretation of these data. Overall, the LNds showed a reduction in cycling amplitude especially at day 5, perhaps resulting from averaging different cycling profiles in the two groups. The s-LNvs experienced phase advance and shortening of their rhythm such that by day 5 they reached the SI maximum at CT6 (although because of the 6 hr resolution of our data, the actual peak might be a few hours later). Thus, in this genotype, the s-LNvs, the DN1s, and the DN2s were synchronous and showed endogenous cycling compatible with the short behavioral period. A significant "time × cell type" ANOVA interaction (we compared the SI of cell types s-LNvs, DN1s, and DN2s, at different time points, at day 2 and 5; see legend to Figure 4) provides support to the view that the advance in the phase of PDP1ε expression occurred first in the DN2s, then in the DN1s, and finally in the s-LNvs. The changes above appeared first at day 2 and were consolidated at day 5, confirmed with a significant "day × time × cell type" ANOVA interaction (Figure 4).

We then overexpressed CRYΔ in PDF$^-$CRY‡ ∩ PDF$^-$CRY* ∩ PDF$^-$CRY$^-$ cells (*tim-GAL4* ∩ *Pdf-GAL80$_{96a}$* > *UAS-cryΔ*), namely in the DNs and in both types of LNds (the pn-LNv was also affected but it has not been analyzed here). Extending the expression of CRYΔ to the PDF$^-$CRY‡ cells caused a broadening in the expression profile of PDP1ε in the DNs, supporting our hypothesis that the PDF$^-$CRY‡ modulate the PDF$^-$CRY$^-$ cells. Especially at day 2, the levels of PDP1ε started to rise earlier (compared to controls) than for PDF$^-$CRY* ∩ PDF$^-$CRY$^-$ > CRYΔ flies, but declined later in all dorsal neurons, suggesting a combination of phase advance and phase delay inputs. The DN2s maintained their shorter molecular cycling while the DN1s and seemingly the DN3s moved to a ∼24 hr period. The LNds, also expressing CRYΔ directly, showed the most pronounced phase delay (supported by a significant "time × cell type" ANOVA interaction) and a reduction in cycling amplitude (although smaller than for the previous genotype). Those differences were maintained at day 5, in agreement with a nonsignificant "day × time × cell type" ANOVA interaction (see legend to Figure 4). The profile of PDP1ε IR in the s-LNvs (not expressing CRYΔ) remained unchanged at day 2 and 5, which, compared to the controls, corresponds to a small delay in phase but no change in period. In this genotype, only the sLNvs and the DN1s showed robust endogenous cycling compatible with the behavioral period.

Overall, only the s-LNvs and the DN1s consistently showed cellular cycling matching the behavioral periods of the flies in all genotypes. We suggest that locomotor activity with simple

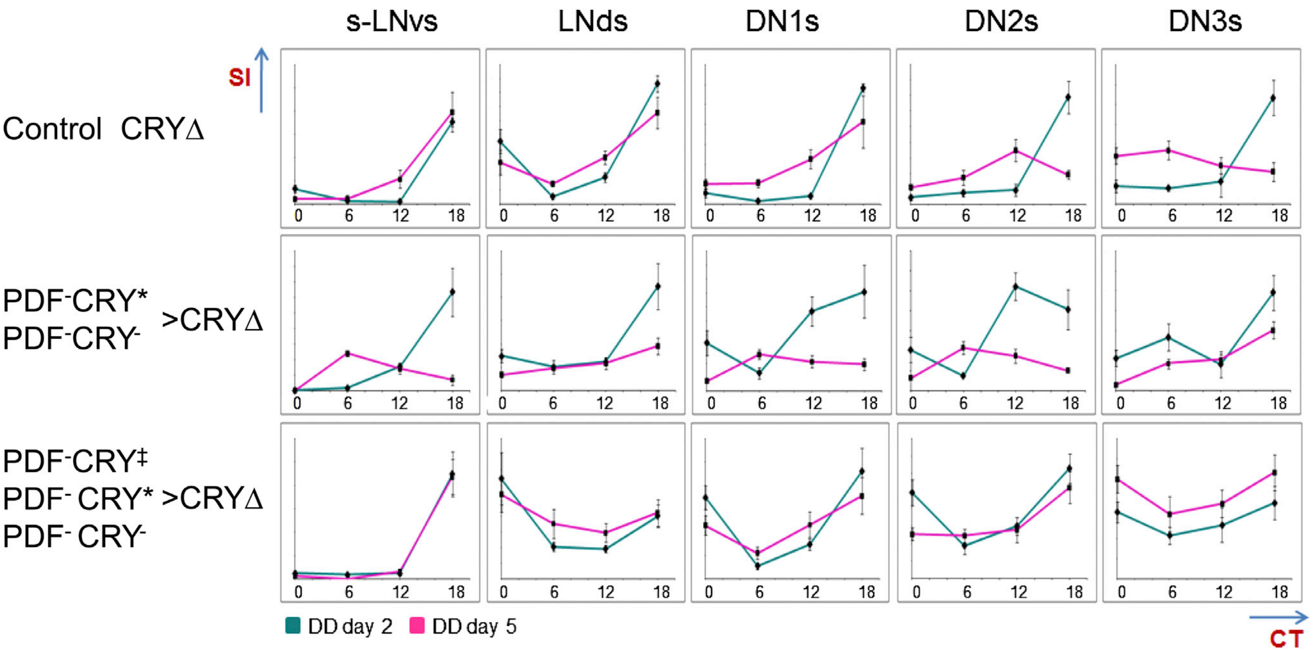

Figure 4. PDP1ε Immunoreactivity after Expression of CRYΔ in Different Groups of Neurons

Staining index (SI, error bars correspond to the standard error of the mean) for PDP1ε at CT0, CT6, CT12, and CT18 during day 2 (green) and 5 (magenta) in DD. ANOVA showed a significant effect ($p < 0.05$) of time of day on SI values, with the following exceptions. Control CRYΔ, DN3s at day 5. PDF$^-$CRY* ∩ PDF$^-$CRY$^-$ > CRYΔ, LNds at day 5 and DN3s at day 5. PDF$^-$CRY‡ ∩ PDF$^-$CRY* ∩ PDF$^-$CRY$^-$ > CRYΔ, LNds at day 5 and DN3s at day 2 and 5. For the two CRYΔ-expressing genotypes, we also tested the interaction among factors by comparing the SI of cell types s-LNvs, DN1s, and DN2s at different time points, at day 2 and 5. (PDF$^-$CRY* ∩ PDF$^-$CRY$^-$ > CRYΔ, ANOVA, Day, $F_{1,215} = 31.09$, $p << 0.01$; Time, $F_{3,215} = 24.83$, $p << 0.01$; Cell type, $F_{2,215} = 6.29$, $p < 0.01$; Day*Time, $F_{3,215} = 18.26$, $p << 0.01$; Day*Cell type, $F_{2,215} = 0.88$, $p = 0.42$; Time*Cell type, $F_{6,215} = 4.50$, $p << 0.01$; Day*Time*Cell type, $F_{6,215} = 3.50$, $p < 0.01$. PDF$^-$CRY‡ ∩ PDF$^-$CRY* ∩ PDF$^-$CRY$^-$ > CRYΔ, ANOVA, Day, $F_{1,193} = 2.33$, $p = 0.13$; Time, $F_{3,193} = 50.73$, $p << 0.01$; Cell type, $F_{2,193} = 36.67$, $p << 0.01$; Day*Time, $F_{3,193} = 2.71$, $p = 0.046$; Day*Cell type, $F_{2,193} = 0.83$, $p = 0.44$; Time*Cell type, $F_{6,193} = 3.06$, $p < 0.01$; Day*Time*Cell type, $F_{6,193} = 0.82$, $p = 0.56$.) PDP1ε did not cycle in l-LNvs, so they are not shown. We did not measure the PDF-negative LNv and the lateral posterior neurons (LPNs) because we were unable to identify them unequivocally in all preparations. For each cluster of neurons, we calculated the average SI per hemisphere (each considered as an independent observation) and we used those values for statistical comparisons. The number of hemispheres analyzed are given below as GENOTYPE, DAY [cell type1 (time points), cell type2 (time points), etc.]. Cell types are s-LNvs, LNds, DN1s, DN2s, DN3s, respectively. Time point are CTO, CT6, CT12, CT18, respectively. Control CRYΔ, DD2 [(9, 10, 11, 8), (9, 8, 11, 7), (10, 9, 11, 9), (10, 6, 10, 8), (10, 10, 10, 6]; DD5 [(11, 9, 12, 14), (11, 9, 11, 14), (9, 10, 9, 11), (10, 5, 9, 10), (10, 9, 10, 12)]. PDF$^-$CRY* ∩ PDF$^-$CRY$^-$ > CRYΔ, DD2 [(11, 9, 10, 9), (11, 9, 10, 10), (12, 7, 10, 10), (11, 8, 9, 10), (11, 8, 8, 10)]; DD5 [(11, 10, 12, 9), (11, 10, 13, 11), (10, 10, 10, 11), (10, 9, 10, 11), (9, 10, 10, 8)]. PDF$^-$CRY‡ ∩ PDF$^-$CRY* ∩ PDF$^-$CRY$^-$ > CRYΔ, DD2 [(9, 13, 10, 8), (9, 10, 10, 8), (8, 7, 10, 8), (8, 5, 10, 7), (8, 7, 9, 8)]; DD5 [(11, 10, 10, 11), (12, 11, 10, 11), (9, 9, 8, 11), (9, 6, 8, 12), (10, 9, 9, 9)]. Genotypes: Control CRYΔ, *w, UAS-cryΔ14.6; +/+; +/+*. PDF$^-$CRY* ∩ PDF$^-$CRY$^-$ > CRYΔ, *w, UAS-cryΔ14.6; tim-GAL4/+; cry-GAL80*$_{2e3m}$/+. PDF$^-$CRY‡ ∩ PDF$^-$CRY* ∩ PDF$^-$CRY$^-$ > CRYΔ, *w, UAS-cryΔ14.6; tim-GAL4/Pdf-GAL80*$_{96a}$; +/+.

rhythmicity requires synchronization between these two groups of neurons, although the contribution of neurons from other clusters may also be significant. In the PDF$^-$CRY* ∩ PDF$^-$CRY$^-$ > CRYΔ (*tim-GAL4* ∩ *cry-GAL80*$_{2e3m}$ > *UAS-cryΔ*) flies, the faster rhythm of the DN2s and perhaps of additional unidentified fast PDF$^-$CRY$^-$ cells was shared by DN1s and s-LNvs. In PDF$^-$CRY‡ ∩ PDF$^-$CRY* ∩ PDF$^-$CRY$^-$ > CRYΔ (*tim-GAL4* ∩ *Pdf-GAL80*$_{96a}$ > *UAS-cryΔ*) individuals, the DN2s were still cycling with a faster pace but, as in wild-type flies, the shorter period did not spread to DN1s and s-LNvs, suggesting this is the role of the highly PDF-responsive PDF$^-$CRY‡ cells (included in the second but not in the first genotype). The expressions of CRYΔ in the DN1ps only [29] did not result in faster behavioral rhythms, showing that the shorter period originates from a different group of neurons (Table S3); see Figure 2C for a possible scenario.

Network Perturbations Can Drive Changes in the Period of Locomotor Activity Independently of Development, although Ontogeny Is Not without Effect

The behavioral changes discussed above might depend on development. We employed *Pdf-Geneswitch* (*Pdf-GS*) [30] to trigger drug-dependent expression of CRYΔ in PDF$^+$CRY$^+$ neurons either in adults only (acute) or through the whole development (chronic). For acute treatment, flies were fed for 48 hr from the day of eclosion with medium supplemented with either 200 μg/ml of the drug RU486 (induction) or with an equal volume of vehicle (80% ethanol, no induction). Note, all flies were raised on this medium and were then examined (on their respective medium) for locomotor activity rhythms. For chronic treatment, flies were exposed to either drug or vehicle during their whole life and adult locomotor activity was examined accordingly. As controls, we tested (heterozygous) driver and effector flies raised under acute or chronic exposure to drug or on vehicle only (Table S4). Under both treatment conditions, we observed a significant, albeit small, lengthening of the period of locomotor activity for CRYΔ-overexpressing flies compared to controls (Figures 5A and 5B).

As a further test we directed the expression of the temperature-sensitive cation channel TRPA1 in PDF$^+$CRY$^+$ and in PDF$^-$CRY* ∩ PDF$^-$CRY$^-$ cells (Figures 5C and 5D; Table S4). This channel is inactive at temperatures below 25°C in *Drosophila* and originally it was reported as being

endogenously expressed in the adult brain in only about a dozen cells, which are not part of the circadian system [31]. However, recent evidence has shown that *trpa1* transcription does occur in at least some cells in each cluster of clock neurons but that the effects of *trpa1*-null mutations on rhythmic behavior are quite modest [32]. Flies overexpressing TRPA1 and (heterozygous) parental controls were raised at 18°C (TRPA1 is inactive) and then tested as adults at 18°C and 28°C (TRPA1 is active). Compared to controls, we would expect a longer and a shorter period for $PDF^{+}CRY^{+}$ > TRPA1 (*Pdf-GAL4* > *UAS-TrpA1*) and $PDF^{-}CRY^{*}$ ∩ $PDF^{-}CRY^{-}$ > TRPA1 (*tim-GAL4* ∩ *cry-GAL80*$_{2e3m}$ > *UAS-TrpA1*) flies at 28°C, respectively. For $PDF^{+}CRY^{+}$ > TRPA1 flies, the increase in period length with temperature was comparable to controls (Figures 5C and 5D). Conversely, we observed a significant decrease in period length for $PDF^{-}CRY^{*}$ ∩ $PDF^{-}CRY^{-}$ > TRPA1 flies at higher temperature but not for controls (Figures 5C and 5D).

In summary, the manipulation of the circadian network limited to adults can affect the free-running period of locomotor activity. However, the modest effects obtained with adult $PDF^{+}CRY^{+}$ cells suggest that the ontogeny of the clock is particularly sensitive to the status of these neurons. Similar conclusions were reached by Depetris-Chauvin et al. [30] and Gorostiza and Ceriani [33]. Conversely, here we show that the $PDF^{-}CRY^{*}$ and the $PDF^{-}CRY^{-}$ cells appear to be less sensitive to developmental effects (Figures 5C and 5D).

Confirming the Model

Stoleru et al. [13] had shown that overexpression of a constitutive active form of the kinase SHAGGY (SGG), although able to shorten the period of locomotor activity when driven in $PDF^{+}CRY^{+}$ cells, was unable to elicit a behavioral effect when directed in $PDF^{-}CRY^{*}$ ∩ $PDF^{-}CRY^{-}$ neurons [13]. This was in spite of causing a faster molecular oscillation in some cells, for instance the DN2s [13], and was considered evidence that those cells are not relevant for free-running behavior. This explanation contradicts our model, so we investigated further.

$PDF^{-}CRY^{*}$ ∩ $PDF^{-}CRY^{-}$ > SGG (*tim-GAL4* ∩ *cry-GAL80*$_{2e3m}$ > *UAS-sgg*) flies showed a free-running period of locomotor activity no different from controls, in agreement with previous results [13] (Figure 6A; Table S5). However, a visual inspection of the activity profiles revealed that these flies, both in LD and DD, showed earlier anticipation of the actual or subjective dark-to-light transition (Figure 6B). Perhaps this anticipation of phase reflects a tendency for the shorter rhythm to propagate to the network, which instead is prevented by other neuronal groups. Previous work has suggested that there is a physical and functional interaction between CRY and SGG [14]. There is little or no CRY in $PDF^{-}CRY^{*}$ and $PDF^{-}CRY^{-}$ neurons, which could preclude SGG from exerting any robust effect. According to our model, the $PDF^{-}CRY^{*}$ repress the $PDF^{+}CRY^{+}$ cells; thus, increasing the output of the former should reduce the resistance of the network to the propagation of the faster rhythm. We coexpressed CRY and SGG and indeed observed a faster free-running period of locomotor activity in $PDF^{-}CRY^{*}$ ∩ $PDF^{-}CRY^{-}$ > SGG, CRY (*tim-GAL4* ∩ *cry-GAL80*$_{2e3m}$ > *UAS-sgg, UAS-cry*) but not in $PDF^{-}CRY^{*}$ ∩ $PDF^{-}CRY^{-}$ > CRY (*tim-GAL4* ∩ *cry-GAL80*$_{2e3m}$ > *UAS-cry*) flies (Figure 6C; Table S5). These results reveal that the DN2s are indeed part of the circuit that generates self-sustained behavior, although they do not exclude that the establishment of a faster rhythm might require additional $PDF^{-}CRY^{-}$ neurons. Implicitly, they also contradict the view of a defined hierarchical organization of the clock under constant conditions.

Considering all results reported above, we favor a model based on a flexible network of circadian neurons, which operates under any environmental condition. Our model complements and extends evidence by others of network organization as opposed to hierarchical dominance among neurons under LD and DD conditions [34–36].

Conclusions

We have used genetic manipulations limited to discrete groups of neurons to unveil the logic governing self-sustained rhythmic locomotor activity in *Drosophila*. Activity rhythms and observations of molecular cycling in defined cellular groups consistently support a model (Figure 2) where endogenous behavioral rhythmicity is based upon synchronization between $PDF^{+}CRY^{+}$ and $PDF^{-}CRY^{-}$ cells. This requires modulation of the $PDF^{-}CRY^{-}$ cells by the $PDF^{+}CRY^{+}$ neurons, largely but not exclusively via PDF signaling, and multistep feedback adjustment of the latter group of neurons by the former. The behavioral outcomes of manipulations that alter but do not destroy the equilibrium of the network reveal that the $PDF^{+}CRY^{+}$ cells have a tendency for driving rhythms longer than 24 hr whereas some $PDF^{-}CRY^{-}$ cells promote rhythms shorter than 24 hr. However, the organization of the network is such that wild-type flies reach a combined ~24 hr oscillation. This requires the $PDF^{-}CRY^{‡}$ and the $PDF^{-}CRY^{*}$ cells that tune, through inhibitory interactions, the relative contribution of the other two groups of neurons to the network. We note that the interposition of the $PDF^{-}CRY^{‡}$ and the $PDF^{-}CRY^{*}$ cells adds delay and signal amplification to the feedback regulation that links the $PDF^{+}CRY^{+}$ to the $PDF^{-}CRY^{-}$ neurons and vice versa.

In conclusion, we have shown that the endogenous behavioral period reflects the nonadditive interplay of all clock cells and not the dominant action of a single group of neurons (i.e., the s-LNvs), as previously suggested. Second, our overall results suggest that intercellular coupling is fundamental for synchronizing different cellular oscillators in a coherent clock, and therefore additional elements must complement the known molecular model focused around transcriptional regulators. Finally, we have shown that excitatory and inhibitory interactions are instrumental for changing the strength and the time delay in the coupling among neurons, resulting in different (collective) behavioral periods. This suggests that contrary to current belief, the period of circadian rhythms is not a fixed feature genetically encoded in a group of neurons. We propose that it is an emerging property of a network of multiple, different circadian oscillators and that it is the wiring of the system, whose logic is genetically encoded, that determines period length. Perhaps not surprisingly, the same principles of negative feedback with amplification and delay, which are central to the TTL model, re-emerge in the constitution of the clock at the intercellular level. We hope this view will inspire formal modeling of the *Drosophila* circadian network. The development of novel tools will be required to investigate how the known molecular mechanisms translate into different cellular features and for the experimental validation of the "logical" connections we postulate in our model. These matters will be the subject of our future work.

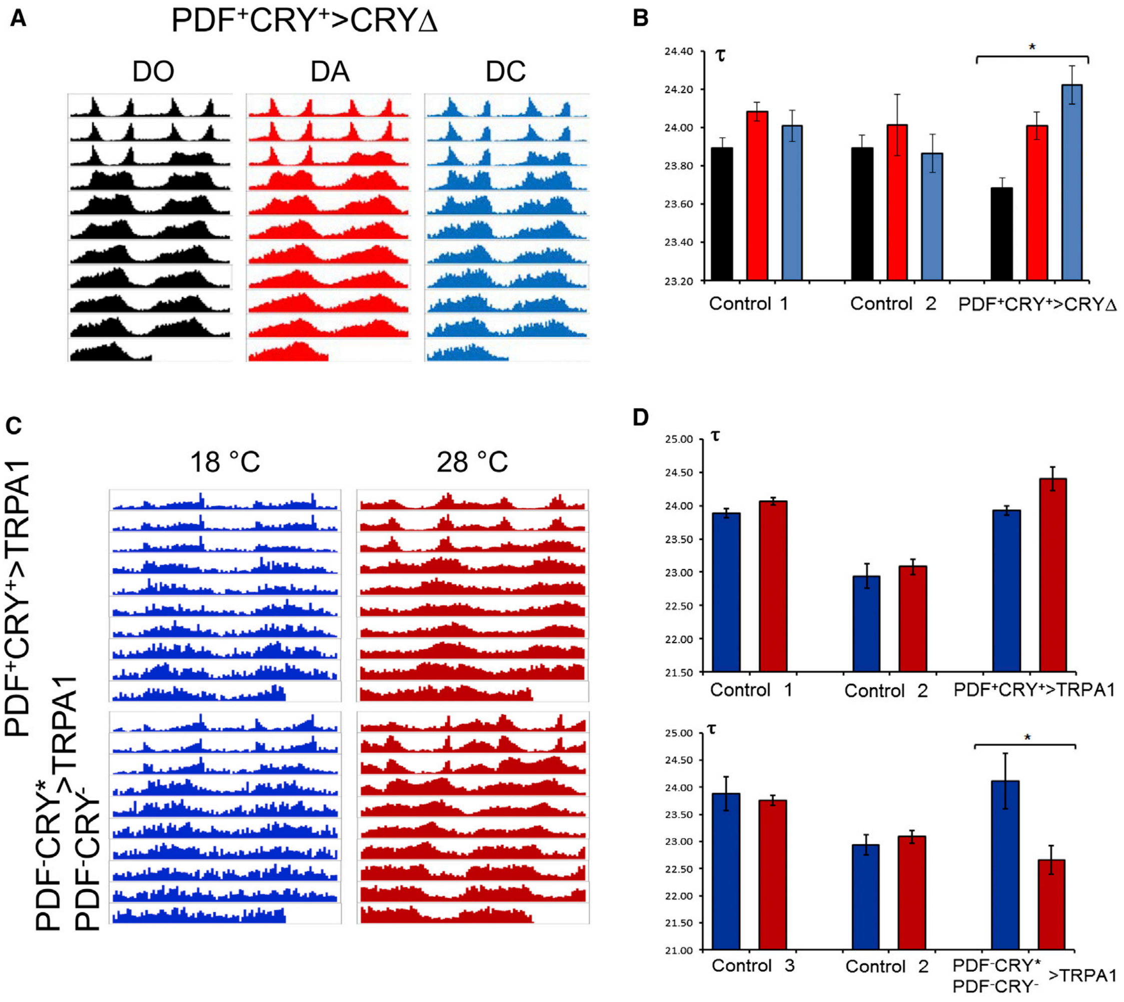

Figure 5. Network Perturbations Independently of Development

(A and B) Expression of CRYΔ in PDF+CRY+ neurons after activation of the *Geneswitch* system by the drug RU486.

(A) Average locomotor activity profiles (3 days LD, 8 days DD) of *Geneswitch* PDF+CRY+ > CRYΔ flies that were never subjected to drug treatment (DO), that were subjected to treatment as adults only (DA), or that were exposed to the drug since early development (DC).

(B) The period of locomotor activity of *Geneswitch* PDF+CRY+ > CRYΔ flies and their parental controls were compared across the three different treatments: DO (black), DA (red), and DC (blue). ANOVA showed a nonsignificant effect of genotype ($F_{2,477} = 0.554$, $p = 0.58$) but a significant effect of treatment ($F_{2,477} = 7.22$, $p < 0.01$) and (asterisk) of the interaction term (genotype × treatment, $F_{4,477} = 2.53$, $p = 0.04$). Post-hoc analyses revealed significant differences comparing DA (Bonferroni, $p < 0.01$) and DC (Bonferroni, $p = 0.04$) to DO but not between DA and DC (Bonferroni, $p = 1$).

Genotypes: *Geneswitch* PDF+CRY+ > CRYΔ, *w, UAS-cryΔ14.6; UAS-CD8GFP/+; Pdf-GS/+*; Control 1, *w; UAS-CD8GFP/+; Pdf-GS/+*; Control 2, *w, UAS-cryΔ14.6; +/+; +/+*. See also Table S4.

(C and D) Expression of the temperature sensitive cation channel TRPA1 in PDF+CRY+ and in PDF−CRY* ∩ PDF−CRY− cells under restrictive (18°C) and permissive (28°C) temperature.

(C) Average locomotor activity profiles (3 days LD, 7 days DD) of both genotypes under both conditions.

(D) The period of locomotor activity of PDF+CRY+ > TRPA1 (top) and PDF−CRY* ∩ PDF−CRY− > TRPA1 (bottom) flies were compared, at the two temperatures (18°C, blue and 28°C, red), to their parental controls. The increase in period length for PDF+CRY+ > TRPA1 flies at higher temperature did not reach significance compared to controls as ANOVA showed a significant effect of genotype ($F_{2,104} = 57.69$, $p << 0.01$) and temperature ($F_{1,104} = 9.35$, $p < 0.01$) but not of the interaction term (genotype × temperature, $F_{2,104} = 1.45$, $p = 0.24$). Conversely, we observed a significant decrease (asterisk) in period length for PDF−CRY* ∩ PDF−CRY− > TRPA1 flies at higher temperature (ANOVA, genotype, $F_{2,75} = 6.22$, $p < 0.01$; temperature, $F_{1,75} = 6.20$, $p = 0.02$; genotype × temperature, $F_{2,75} = 6.32$, $p < 0.01$). Genotypes: PDF+CRY+ > TRPA1, *yw; Pdf-GAL4/+; +/UAS-TrpA1*; Control 1, *yw; Pdf-GAL4/+; +/+*; Control 2 *w; +/+; +/ UAS-TrpA1*; PDF−CRY* ∩ PDF−CRY− > TRPA1 *yw; tim-GAL4/+; cry-GAL80$_{2e3m}$/ UAS-TrpA1*; Control 3, *w; tim-GAL4/+; cry-GAL80$_{2e3m}$/+*. See also Table S4.

Supplemental Information

Supplemental Information includes three figures, five tables, and Supplemental Experimental Procedures and can be found with this article online at http://dx.doi.org/10.1016/j.cub.2014.08.023.

Author Contributions

S.D. and E.R. generated the hypotheses. S.D., C.N.H., and E.R. designed the experiments. S.D., C.N.H., Ö.Ö., M.H., and E.R. performed experiments and analyzed data. S.D., C.P.K., and E.R. wrote the manuscript.

Acknowledgments

We thank J. Blau, P. Emery, P. Hardin, K. Moffat, M. Rosbash, F. Rouyer, A. Sehgal, F. Ceriani, and R. Stanewsky for reagents. The α-PDF monoclonal antibody developed by J. Blau was obtained from the Developmental Studies Hybridoma Bank developed under the auspices of the NICHD and maintained by Department of Biology, The University of Iowa. We

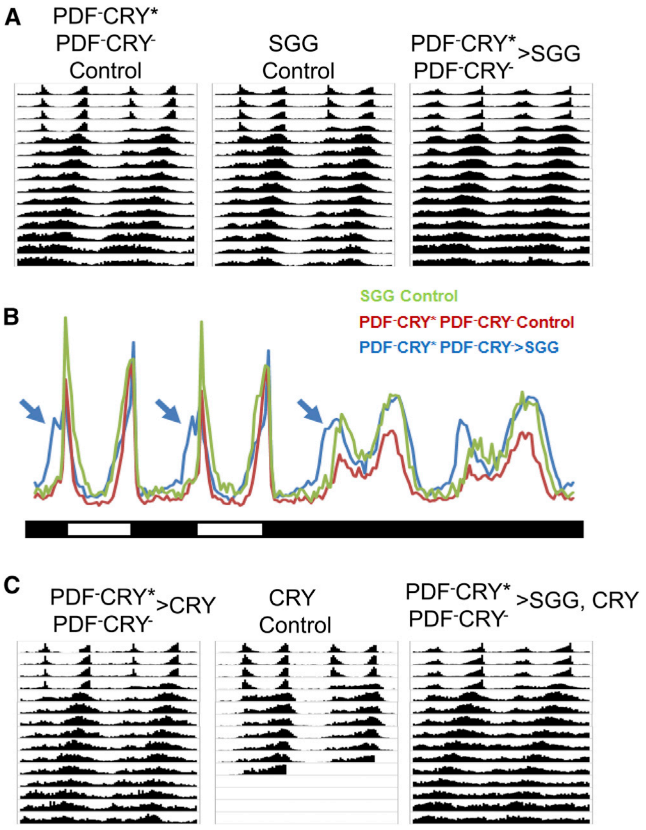

Figure 6. Ectopic Expression of CRY Reveals a Functional Interaction with SGG

(A) Average locomotor activity profiles (4 days LD, 11 days DD) of PDF$^-$CRY* ∩ PDF$^-$CRY$^-$ > SGG flies and controls.

(B) Same data as in (A) but limited to the last 2 days of LD and first 2 days of DD. The profile for PDF$^-$CRY* ∩ PDF$^-$CRY$^-$ > SGG flies (blue) shows earlier anticipation of the dark-to-light transitions (blue arrows) compared to the other genotypes (PDF$^-$CRY* ∩ PDF$^-$CRY$^-$ Control, red and SGG Control, green).

(C) Average locomotor activity profiles (4 days LD, 11 days DD) of PDF$^-$CRY* ∩ PDF$^-$CRY$^-$ > CRY, CRY Control and PDF$^-$CRY* ∩ PDF$^-$CRY$^-$ > SGG, CRY flies. Only the latter genotype showed a shorter period of locomotor activity (see Table S5). Genotypes: PDF$^-$CRY* ∩ PDF$^-$CRY$^-$ > SGG, *w, UAS-sgg; tim-GAL4/+; cry-GAL80*$_{2e3m}$/ +; SGG Control, *w, UAS-sgg; +/+; +/+*; PDF$^-$CRY* ∩ PDF$^-$CRY$^-$ Control, *w; tim-GAL4/+; cry-GAL80*$_{2e3m}$/+; PDF$^-$CRY* ∩ PDF$^-$CRY$^-$ > CRY, *w; tim-GAL4/+; cry-GAL80*$_{2e3m}$*/UAS-HAcry16.1*; CRY Control, *w; +/+; +/UAS-HAcry16.1*; PDF$^-$CRY* ∩ PDF$^-$CRY$^-$ > SGG, CRY, *w, UAS-sgg; tim-GAL4/+; cry-GAL80*$_{2e3m}$*/UAS-HAcry16.1*. See also Table S5.

acknowledge funding from the BBSRC (S.D. and C.N.H., grants BB/C003241/1 and BB/F008988/1; M.H., doctoral studentship) and the NC3Rs (Ö.Ö., G1100597).

Received: March 29, 2014
Revised: July 12, 2014
Accepted: August 13, 2014
Published: September 11, 2014

References

1. Özkaya, Ö., and Rosato, E. (2012). The circadian clock of the fly: a neurogenetics journey through time. Adv. Genet. *77*, 79–123.
2. Stanewsky, R., Kaneko, M., Emery, P., Beretta, B., Wager-Smith, K., Kay, S.A., Rosbash, M., and Hall, J.C. (1998). The cry^b mutation identifies cryptochrome as a circadian photoreceptor in *Drosophila*. Cell *95*, 681–692.
3. Emery, P., So, W.V., Kaneko, M., Hall, J.C., and Rosbash, M. (1998). CRY, a *Drosophila* clock and light-regulated cryptochrome, is a major contributor to circadian rhythm resetting and photosensitivity. Cell *95*, 669–679.
4. Rosato, E., Codd, V., Mazzotta, G., Piccin, A., Zordan, M., Costa, R., and Kyriacou, C.P. (2001). Light-dependent interaction between *Drosophila* CRY and the clock protein PER mediated by the carboxy terminus of CRY. Curr. Biol. *11*, 909–917.
5. Dissel, S., Codd, V., Fedic, R., Garner, K.J., Costa, R., Kyriacou, C.P., and Rosato, E. (2004). A constitutively active cryptochrome in *Drosophila melanogaster*. Nat. Neurosci. *7*, 834–840.
6. Mazzotta, G., Rossi, A., Leonardi, E., Mason, M., Bertolucci, C., Caccin, L., Spolaore, B., Martin, A.J., Schlichting, M., Grebler, R., et al. (2013). Fly cryptochrome and the visual system. Proc. Natl. Acad. Sci. USA *110*, 6163–6168.
7. Yoshii, T., Todo, T., Wülbeck, C., Stanewsky, R., and Helfrich-Förster, C. (2008). Cryptochrome is present in the compound eyes and a subset of *Drosophila's* clock neurons. J. Comp. Neurol. *508*, 952–966.
8. Dolezelova, E., Dolezel, D., and Hall, J.C. (2007). Rhythm defects caused by newly engineered null mutations in *Drosophila*'s *cryptochrome* gene. Genetics *177*, 329–345.
9. Fogle, K.J., Parson, K.G., Dahm, N.A., and Holmes, T.C. (2011). CRYPTOCHROME is a blue-light sensor that regulates neuronal firing rate. Science *331*, 1409–1413.
10. Helfrich-Förster, C., Yoshii, T., Wülbeck, C., Grieshaber, E., Rieger, D., Bachleitner, W., Cusamano, P., and Rouyer, F. (2007). The lateral and dorsal neurons of *Drosophila* melanogaster: new insights about their morphology and function. Cold Spring Harb. Symp. Quant. Biol. *72*, 517–525.
11. Stoleru, D., Peng, Y., Agosto, J., and Rosbash, M. (2004). Coupled oscillators control morning and evening locomotor behaviour of *Drosophila*. Nature *431*, 862–868.
12. Grima, B., Chélot, E., Xia, R., and Rouyer, F. (2004). Morning and evening peaks of activity rely on different clock neurons of the *Drosophila* brain. Nature *431*, 869–873.
13. Stoleru, D., Peng, Y., Nawathean, P., and Rosbash, M. (2005). A resetting signal between *Drosophila* pacemakers synchronizes morning and evening activity. Nature *438*, 238–242.
14. Stoleru, D., Nawathean, P., Fernández, M.P., Menet, J.S., Ceriani, M.F., and Rosbash, M. (2007). The *Drosophila* circadian network is a seasonal timer. Cell *129*, 207–219.
15. Sehgal, A., Rothenfluh-Hilfiker, A., Hunter-Ensor, M., Chen, Y., Myers, M.P., and Young, M.W. (1995). Rhythmic expression of *timeless*: a basis for promoting circadian cycles in *period* gene autoregulation. Science *270*, 808–810.
16. Renn, S.C., Park, J.H., Rosbash, M., Hall, J.C., and Taghert, P.H. (1999). A *pdf* neuropeptide gene mutation and ablation of PDF neurons each cause severe abnormalities of behavioral circadian rhythms in *Drosophila*. Cell *99*, 791–802.
17. Emery, P., Stanewsky, R., Helfrich-Förster, C., Emery-Le, M., Hall, J.C., and Rosbash, M. (2000). *Drosophila* CRY is a deep brain circadian photoreceptor. Neuron *26*, 493–504.
18. Shafer, O.T., Helfrich-Förster, C., Renn, S.C., and Taghert, P.H. (2006). Reevaluation of *Drosophila melanogaster*'s neuronal circadian pacemakers reveals new neuronal classes. J. Comp. Neurol. *498*, 180–193.
19. Nitabach, M.N., Wu, Y., Sheeba, V., Lemon, W.C., Strumbos, J., Zelensky, P.K., White, B.H., and Holmes, T.C. (2006). Electrical hyperexcitation of lateral ventral pacemaker neurons desynchronizes downstream circadian oscillators in the fly circadian circuit and induces multiple behavioral periods. J. Neurosci. *26*, 479–489.
20. Vanin, S., Bhutani, S., Montelli, S., Menegazzi, P., Green, E.W., Pegoraro, M., Sandrelli, F., Costa, R., and Kyriacou, C.P. (2012). Unexpected features of *Drosophila* circadian behavioural rhythms under natural conditions. Nature *484*, 371–375.
21. Menegazzi, P., Vanin, S., Yoshii, T., Rieger, D., Hermann, C., Dusik, V., Kyriacou, C.P., Helfrich-Förster, C., and Costa, R. (2013). *Drosophila* clock neurons under natural conditions. J. Biol. Rhythms *28*, 3–14.
22. Sheeba, V., Fogle, K.J., Kaneko, M., Rashid, S., Chou, Y.T., Sharma, V.K., and Holmes, T.C. (2008a). Large ventral lateral neurons modulate arousal and sleep in *Drosophila*. Curr. Biol. *18*, 1537–1545.
23. Sheeba, V., Sharma, V.K., Gu, H., Chou, Y.T., O'Dowd, D.K., and Holmes, T.C. (2008b). Pigment dispersing factor-dependent and -independent circadian locomotor behavioral rhythms. J. Neurosci. *28*, 217–227.

24. Baines, R.A., Uhler, J.P., Thompson, A., Sweeney, S.T., and Bate, M. (2001). Altered electrical properties in *Drosophila* neurons developing without synaptic transmission. J. Neurosci. *21*, 1523–1531.
25. Nitabach, M.N., Blau, J., and Holmes, T.C. (2002). Electrical silencing of *Drosophila* pacemaker neurons stops the free-running circadian clock. Cell *109*, 485–495.
26. Cusumano, P., Klarsfeld, A., Chélot, E., Picot, M., Richier, B., and Rouyer, F. (2009). PDF-modulated visual inputs and cryptochrome define diurnal behavior in *Drosophila*. Nat. Neurosci. *12*, 1431–1437.
27. Thum, A.S., Knapek, S., Rister, J., Dierichs-Schmitt, E., Heisenberg, M., and Tanimoto, H. (2006). Differential potencies of effector genes in adult *Drosophila*. J. Comp. Neurol. *498*, 194–203.
28. Tanoue, S., Krishnan, P., Krishnan, B., Dryer, S.E., and Hardin, P.E. (2004). Circadian clocks in antennal neurons are necessary and sufficient for olfaction rhythms in *Drosophila*. Curr. Biol. *14*, 638–649.
29. Zhang, L., Chung, B.Y., Lear, B.C., Kilman, V.L., Liu, Y., Mahesh, G., Meissner, R.A., Hardin, P.E., and Allada, R. (2010). DN1(p) circadian neurons coordinate acute light and PDF inputs to produce robust daily behavior in *Drosophila*. Curr. Biol. *20*, 591–599.
30. Depetris-Chauvin, A., Berni, J., Aranovich, E.J., Muraro, N.I., Beckwith, E.J., and Ceriani, M.F. (2011). Adult-specific electrical silencing of pacemaker neurons uncouples molecular clock from circadian outputs. Curr. Biol. *21*, 1783–1793.
31. Hamada, F.N., Rosenzweig, M., Kang, K., Pulver, S.R., Ghezzi, A., Jegla, T.J., and Garrity, P.A. (2008). An internal thermal sensor controlling temperature preference in *Drosophila*. Nature *454*, 217–220.
32. Lee, Y., and Montell, C. (2013). *Drosophila* TRPA1 functions in temperature control of circadian rhythm in pacemaker neurons. J. Neurosci. *33*, 6716–6725.
33. Gorostiza, E.A., and Ceriani, M.F. (2013). Retrograde bone morphogenetic protein signaling shapes a key circadian pacemaker circuit. J. Neurosci. *33*, 687–696.
34. Sheeba, V., Fogle, K.J., and Holmes, T.C. (2010). Persistence of morning anticipation behavior and high amplitude morning startle response following functional loss of small ventral lateral neurons in *Drosophila*. PLoS ONE *5*, e11628.
35. Potdar, S., and Sheeba, V. (2012). Large ventral lateral neurons determine the phase of evening activity peak across photoperiods in *Drosophila melanogaster*. J. Biol. Rhythms *27*, 267–279.
36. Yao, Z., and Shafer, O.T. (2014). The *Drosophila* circadian clock is a variably coupled network of multiple peptidergic units. Science *343*, 1516–1520.
37. Im, S.H., Li, W., and Taghert, P.H. (2011). PDFR and CRY signaling converge in a subset of clock neurons to modulate the amplitude and phase of circadian behavior in *Drosophila*. PLoS ONE *6*, e18974.

Current Biology, Volume *24*

Supplemental Information

The Logic of Circadian Organization in *Drosophila*

Stephane Dissel, Celia N. Hansen, Özge Özkaya, Matthew Hemsley, Charalambos P. Kyriacou, and Ezio Rosato

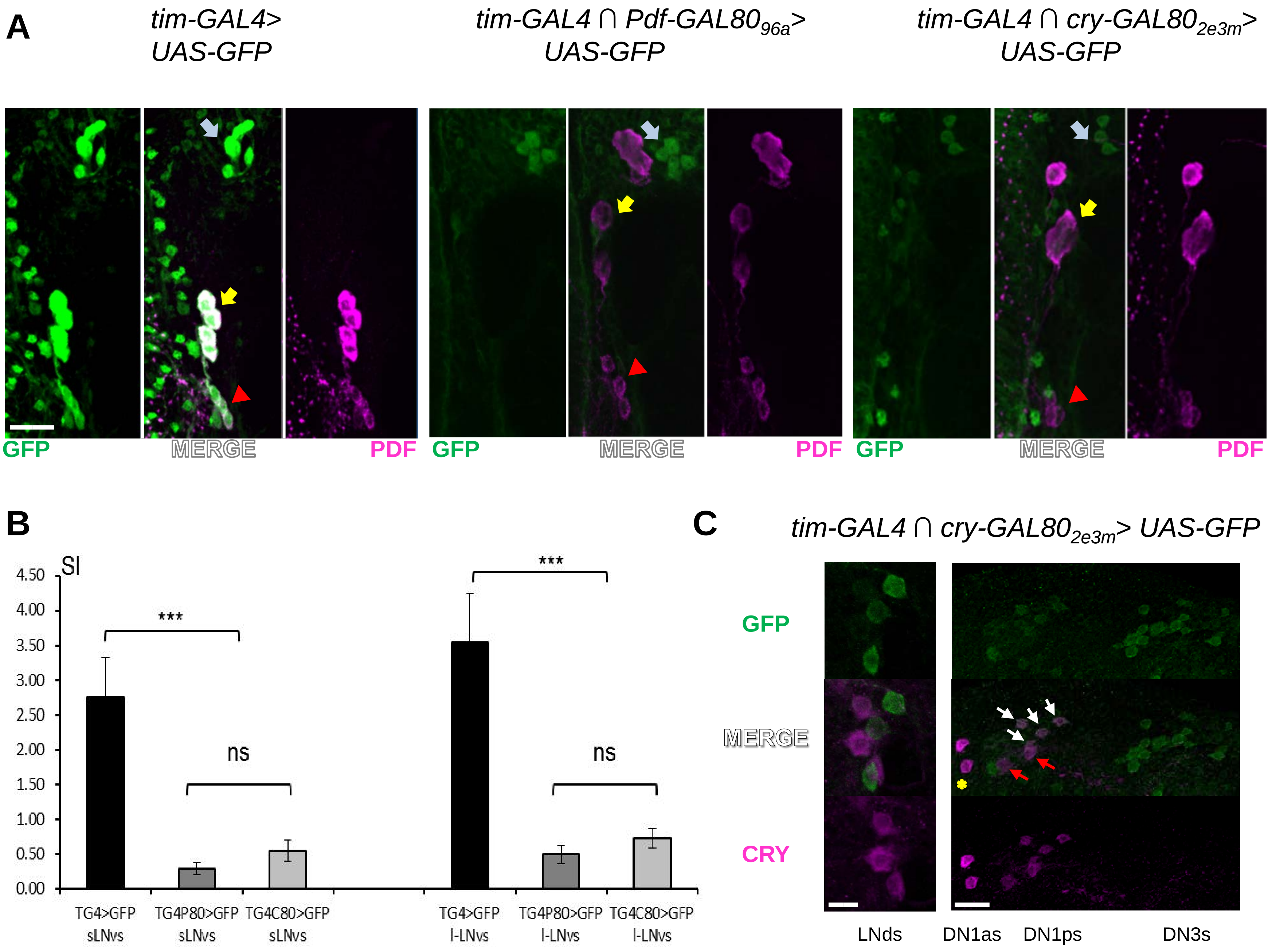
A
tim-GAL4>
UAS-GFP
tim-GAL4 ∩ Pdf-GAL80 96a>
UAS-GFP
tim-GAL4 ∩ cry-GAL80 2e3m>
UAS-GFP
GFP
MERGE
PDF
GFP
MERGE
PDF
GFP
MERGE
PDF
B
SI
4.50
4.00
3.50
3.00
2.50
2.00
1.50
1.00
0.50
0.00
***
ns
***
ns
TG4>GFP
sLNvs
TG4P80>GFP
sLNvs
TG4C80>GFP
sLNvs
TG4>GFP
l-LNvs
TG4P80>GFP
l-LNvs
TG4C80>GFP
l-LNvs
C
tim-GAL4 ∩ cry-GAL80 2e3m> UAS-GFP
GFP
MERGE
CRY
LNds
DN1as
DN1ps
DN3s

Figure S1. *Pdf-GAL8096a* and *cry-GAL80*$_{2e3m}$ mediated repression of *tim-GAL4* activity in clock neurons. Related to Figure 1.

(A) Anti-GFP immunoreactivity (green) in *tim-GAL4>UAS-GFP*, *tim-GAL4* ∩ *Pdf-GAL80*$_{96a}$*>UAS-GFP* and *tim-GAL4* ∩ *cry-GAL80*$_{2e3m}$*>UAS-GFP* flies. Anti-PDF staining (magenta) was used as a cellular marker. Intense GFP staining of the s-LNvs (red arrowheads) and l-LNvs (yellow arrows) was observed in *tim-GAL4>UAS-GFP* but not in the two GAL80 expressing genotypes. Six LNds (blue arrows) were observed in *tim-GAL4>UAS-GFP* and *tim-GAL4*, *Pdf-GAL80*$_{96a}$*>UAS-GFP* flies. Unexpectedly, three LNds were consistently observed in *tim-GAL4*, *cry-GAL80*$_{2e3m}$*>UAS-GFP* flies. Size bar = 20 µm.

(B) Anti-GFP immunoreactivity was quantified in s-LNvs (right) and l-LNvs (left) of *tim-GAL4>UAS-GFP, tim-GAL4* ∩ *Pdf-GAL80*$_{96a}$*>UAS-GFP* and *tim-GAL4* ∩ *cry-GAL80*$_{2e3m}$*>UAS-GFP* flies and reported as Staining Index (SI); the error bars correspond to the standard error of the mean (SEM). SI was calculated as described in Materials and Methods, with the exception that values were not normalized for the expected number of cells according to cell type. The two GAL80 expressing strains showed a significant reduction in SI compared to *tim-GAL4>UAS-GFP* flies for both cell types (ANOVA, Genotype $F_{2,66}$=39.79, P<<0.01; Cell type $F_{1,66}$=2.10, P=0.15, Genotype*Cell type $F_{2,66}$=0.52, P=0.60). Importantly, there was no difference between the GAL80 carrying strains (Bonferroni post-hoc: *tim-GAL4>UAS-GFP vs. tim-GAL4* ∩ *cry-GAL80>UAS-GFP*, P<<0.01; *tim-GAL4>UAS-GFP vs. tim-GAL4* ∩ *Pdf-GAL80>UAS-GFP*, P<<0.01; *tim-GAL4* ∩ *Pdf-GAL80>UAS-GFP vs. tim-GAL4* ∩ *cry-GAL80>UAS-GFP*, P=1.00). We analysed 10, 14, and 12 hemispheres for the genotypes *tim-GAL4>UAS-GFP*, *tim-GAL4* ∩ *Pdf-GAL80>UAS-GFP* and *tim-GAL4* ∩ *cry-GAL80>UAS-GFP*, respectively.

(C) Published observations suggest that the *cry* promoter is expressed in all 6 LNds in *cry-GAL4* transgenics [11, 18], but that the CRY protein is robustly detected in only 3 LNds in wild type flies [7]. This would suggest that the GFP-positive and negative LNds in *tim-GAL4* ∩ *cry-GAL80*$_{2e3m}$*>UAS-GFP* as seen in (A) correspond to the CRY immunonegative and immunopositive LNds, respectively. Anti-GFP (green) and anti-CRY (magenta) immunoreactivities did not overlap in the LNds of *tim-GAL4* ∩ *cry-GAL80>UAS-GFP* flies, verifying this hypothesis (left panel, size bar = 5 µm.). We also compared anti-GFP and anti-CRY staining in the dorsal brain (right panel, size bar = 15 µm.). Two DN1as (yellow asterisk) and two DN1ps (red arrows) showed anti-CRY staining only (*cry* expression is high). Four DN1ps (white arrows) were labelled by both anti-GFP and anti-CRY antibodies (*cry* expression is low). The DN3s showed anti-GFP immunoreactivity only (*cry* expression is very low or absent).

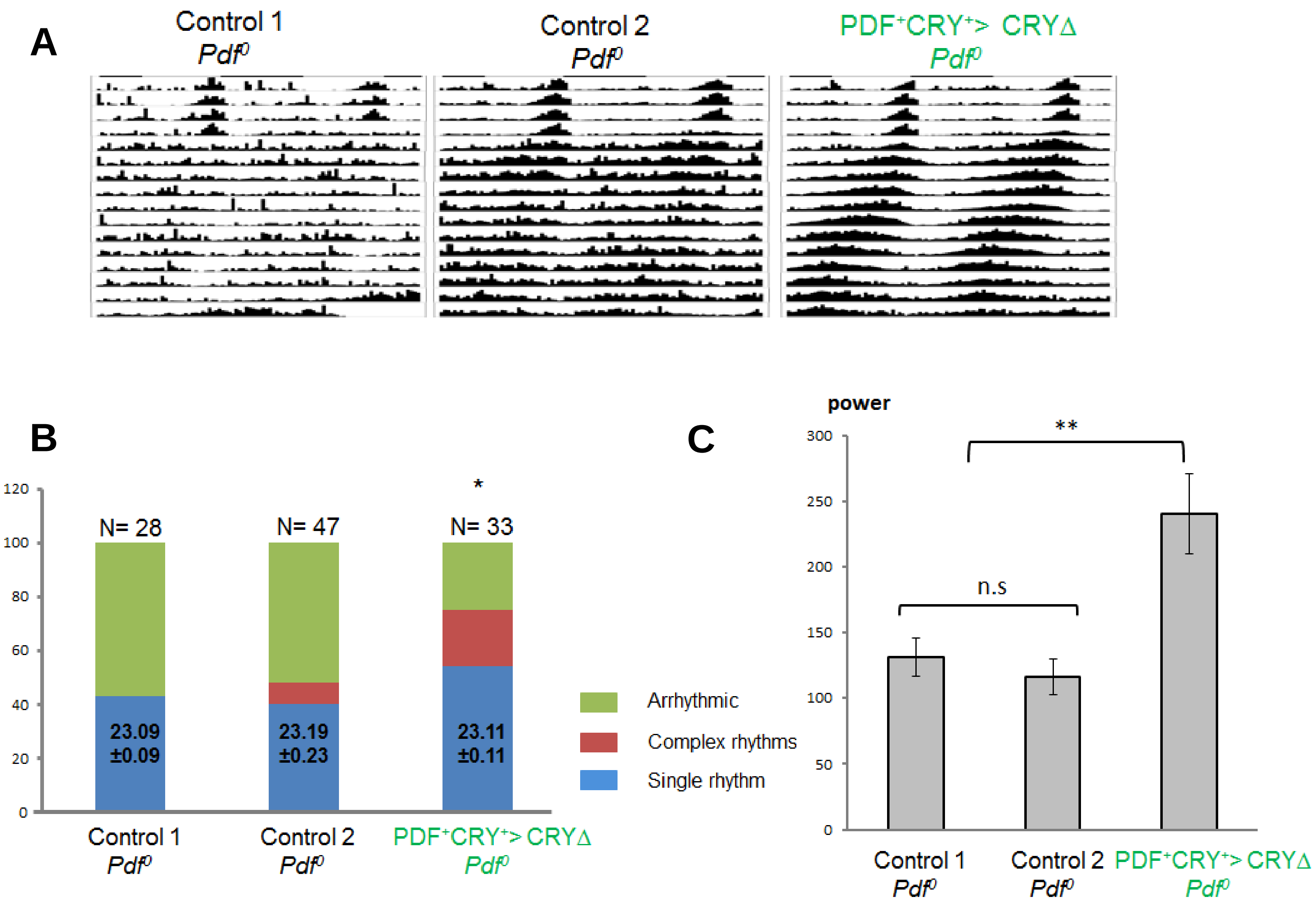

A
Control 1
Pdf0
Control 2
Pdf0
PDF+CRY+> CRYΔ
Pdf0
B
120
100
80
60
40
20
0
*
N= 28
N= 47
N= 33
23.09
±0.09
23.19
±0.23
23.11
±0.11
Arrhythmic
Complex rhythms
Single rhythm
Control 1
Pdf0
Control 2
Pdf0
PDF+CRY+> CRYΔ
Pdf0
C
power
300
250
200
150
100
50
0
**
n.s
Control 1
Pdf0
Control 2
Pdf0
PDF+CRY+> CRYΔ
Pdf0

Figure S2.Expression of CRYΔ in PDF+CRY+ neurons improves behavioural rhythmicity in *Pdf*^{0} flies suggesting a link between PDF+CRY+ and PDF-CRY- cells. Related to Figure 2.
Although mainly arrhythmic, a moderate proportion of *Pdf*^{0} mutants are still rhythmic in DD but have a (~ 1 h) shorter activity period [16], suggesting that faster PDF-CRY- neurons might be responsible for it. A significant increase in the number of rhythmic individuals (from ~30% to ~60%) has been reported for PDF+CRY+>NaChBac (*Pdf*^{0}) flies (*Pdf-GAL4, Pdf*^{0}*/Pdf*^{0}*>UAS-NaChBac*) [23]. We confirmed those results by testing PDF+CRY+>CRYΔ (*Pdf*^{0}) mutants (*Pdf-GAL4, Pdf*^{0}*/Pdf*^{0}*>UAS-cryΔ*) and parental controls.
(A) Average locomotor activity profiles of flies showing 4 days in LD 12:12 and 12 days in DD. The majority of PDF+CRY+>CRYΔ (*Pdf*^{0}) flies sustained rhythmic behaviour under DD, contrary to *Pdf*^{0} controls.
(B) Each column shows the percentage of single rhythm (blue) complex rhythms (red) and arrhythmic flies (green) per genotype. The proportion of rhythmic (single+complex) flies was significantly higher for the PDF+CRY+>CRYΔ (*Pdf*^{0}) genotype than controls (two-tailed Fisher's exact test, P<0.01), this is indicated by an asterisk. The (average ±SEM) period of locomotor activity of single rhythm flies is shown in the blue sections. There were no significant differences in period among genotypes (ANOVA, $F_{2,46}$=0.10, P=0.91).
(C) Columns represent the mean power (robustness) calculated for single rhythm flies of each genotype. Error bars correspond to SEM. The power was significantly higher for the PDF+CRY+>CRYΔ (*Pdf*^{0}) genotype than controls (ANOVA, $F_{2,46}$=9.95, P<0.001. Bonferroni post-hoc comparisons: Control1 (*Pdf*^{0}) *vs*. Control2 (*Pdf*^{0}), P=1.00. Control1 (*Pdf*^{0}) *vs*. PDF+CRY+>CRYΔ (*Pdf*^{0}), P=0.006. Control2 (*Pdf*^{0}) *vs*. PDF+CRY+>CRYΔ (*Pdf*^{0}), P<0.001).
Genotypes: PDF+CRY+>CRYΔ (*Pdf*^{0}): *w, UAS-cryΔ14.6; Pdf-GAL4/+; Pdf*^{01}*/Pdf*^{01}. Control1 *Pdf*^{0}: *w; Pdf-GAL4/+; Pdf*^{01}*/Pdf*^{01}; Control2 *Pdf*^{0}: *w, UAS- cryΔ14.6; +/+; Pdf*^{01}*/Pdf*^{01}. N=total number of flies. The same number of flies contributed to the activity profiles shown in (A).
Thus, in *Pdf*^{0} mutants the overexpression of NaChBac or CRYΔ restricted to the PDF+CRY+ cells (*Pdf-GAL4* driver) results in more rhythmic flies with shorter behavioural periods. The latter suggest the involvement of fast PDF-CRY- neurons, although the *Pdf-GAL4* driver does not directly target them. We conclude that a positive, PDF-independent connection likely links the PDF+CRY+ and the PDF-CRY- cells. In support of this hypothesis, we notice that the s-LNvs, which project to the dorsal area of the brain where the PDF-CRY- cells are located, express a small neurotransmitter in addition to PDF [S1, S2]. This signalling mechanism seems important as preferential activation of the s-LNvs through a membrane tethered PDF molecule (PDF+CRY+>t-PDF stimulates predominantly the s-LNvs because the l-LNvs express very little or no PDFR, see ref. [37]) results in short period rhythms [S1].
Furthermore, these data suggest that CRYΔ is an activator of neuronal function but weaker than NaChBac, which possibly explains why the short period behavioural component is absent in PDF+CRY+>CRYΔ flies (see also Figures S3).

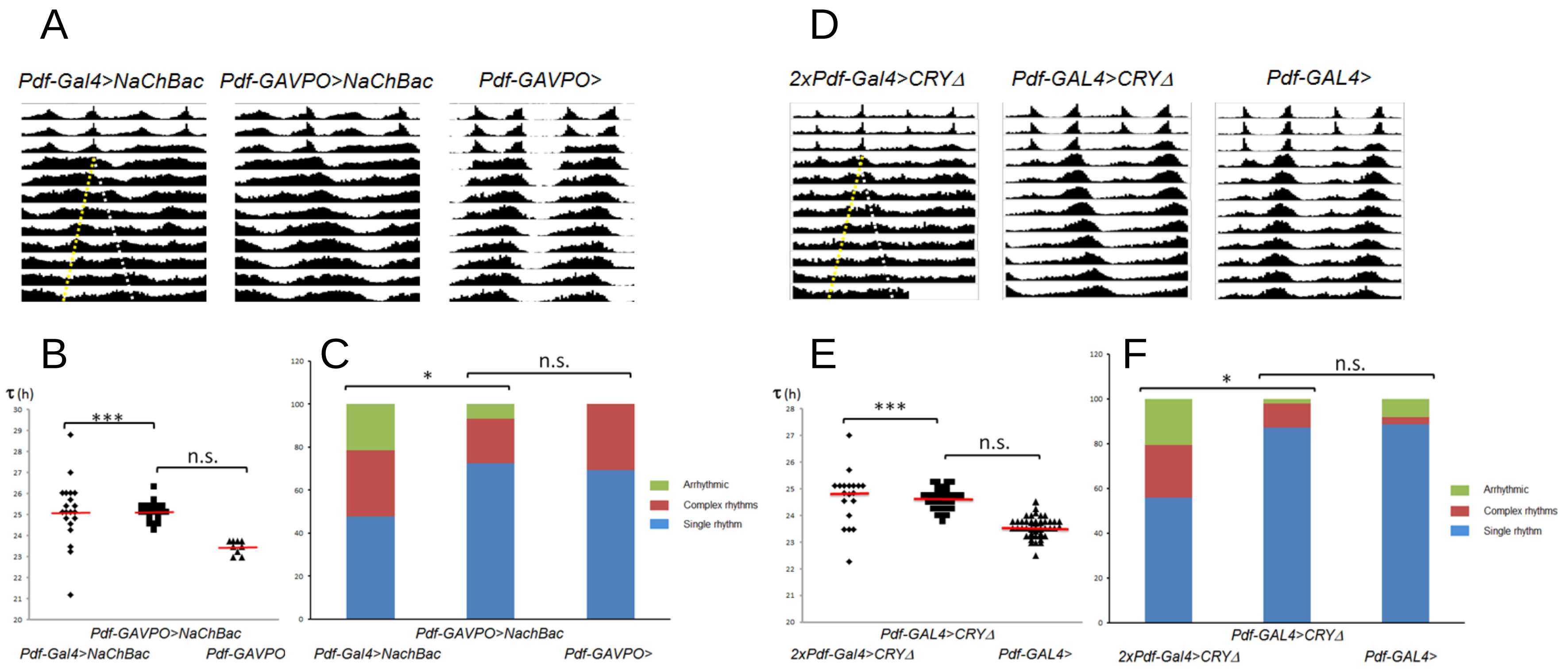
A
Pdf-Gal4>NaChBac
Pdf-GAVPO>NaChBac
Pdf-GAVPO>
D
2xPdf-Gal4>CRYΔ
Pdf-GAL4>CRYΔ
Pdf-GAL4>
B
τ (h)
***
n.s.
Pdf-GAVPO>NaChBac
Pdf-Gal4>NaChBac
Pdf-GAVPO
C
*
n.s.
Arrhythmic
Complex rhythms
Single rhythm
Pdf-GAVPO>NachBac
Pdf-Gal4>NachBac
Pdf-GAVPO>
E
τ (h)
***
n.s.
Pdf-GAL4>CRYΔ
2xPdf-Gal4>CRYΔ
Pdf-GAL4>
F
*
n.s.
Arrhythmic
Complex rhythms
Single rhythm
Pdf-GAL4>CRYΔ
2xPdf-Gal4>CRYΔ
Pdf-GAL4>

Figure S3. NaChBac and CRYΔ generate similar phenotypes when expressed in PDF+CRY+ neurons, but at different levels. Related to Figure 3.

(A-B) To reduce the expression of NachBac we implemented in *Drosophila* a recently described chimeric GAL4-VIVID-P65 transcription factor called GAVPO that requires induction by blue light to initiate UAS-dependent transcription. GAVPO can only dimerize (which is required for transcriptional activity) in the presence of light but the dimer is then stable until cleared by cellular turnover. [S3]. We generated a *Pdf-GAVPO* line and we used it to drive NaChBac expression under LD 12:12; then we released the flies into DD for nine days so that active GAVPO would not be further produced.

(A) Average locomotor activity profiles of NaChBac expressing and control flies showing 3 days in LD 12:12 and 9 days in DD. The DD activity profile of *Pdf-GAL4>NaChBac* flies showed a long and a short activity components. The latter was lost when NaChBac overexpression was reduced in DD using the *Pdf-GAVPO* driver. *Pdf-GAVPO>* flies were included as a control. The white and the yellow hatched lines indicate the long and the short activity components, respectively.

(B) Scatter plot showing the distribution of free run single rhythm periodicities for *Pdf-GAL4>NaChBac*, *Pdf-GAVPO>NaChBac* and *Pdf-GAVPO>* flies. *Pdf-GAL4>NaChBac* had significantly higher variance (two-tailed F-test, P<<0.01) than *Pdf-GAVPO>NaChBac* flies, whereas the latter were not significantly different from controls (two-tailed F-test, P=0.31). Red Bar = median.

(C) Columns represent the percentage of single rhythm (blue), complex rhythm (red) and arrhythmic (green) individuals for *Pdf-GAL4>NaChBac*, *Pdf-GAVPO>NaChBac* and *Pdf-GAVPO>* flies in DD. Reducing DD expression of NaChBac *via Pdf-GAVPO* resulted in an increased proportion of single rhythm flies, as expected (one-tailed Fisher's exact test, P=0.03). The proportions of single rhythm flies were not different between *Pdf-GAVPO>NaChBac* and *Pdf-GAVPO>* controls (one-tailed Fisher's exact test, P=0.55).

Genotypes: *yw; Pdf-GAL4/UAS-NaChBac4;+/+* (N=42), *w; +/UAS-NaChBac4; Pdf-GAVPO/+* (N=29), *w; +/+; Pdf-GAVPO/+* (N=13). All genotypes were tested in a single, parallel experiment.

(D-F) We increased the expression of CRYΔ in PDF+CRY+ neurons by combining two copies of the driver, namely *2xPdf-GAL4.*

(D) Average locomotor activity profiles of CRYΔ expressing and control flies showing 3 days in LD 12:12 and 9 days in DD. The DD activity profile of *Pdf-GAL4>CRYΔ* flies showed a long activity components. An additional short period component was acquired when CRYΔ overexpression was increased by virtue of two copies of the *Pdf-GAL4* driver. *Pdf-GAL4>* flies were included as a control. The white and the yellow hatched lines indicate the long and the short activity components, respectively.

(E) Scatter plot showing the distribution of free run single rhythm periodicities for 2x*Pdf-GAL4>CRYΔ*, *Pdf-GAL4>CRYΔ* and *Pdf-GAL4>* flies. 2x*Pdf-GAL4>CRYΔ* had significantly higher variance (two-tailed F-test, P<<0.01) than *Pdf-GAL4>CRYΔ* flies, whereas the latter were not significantly different from controls (two-tailed F-test, P=0.90). Red Bar = median.

(F) Columns represent the percentage of single rhythm (blue), complex rhythm (red) and arrhythmic (green) individuals for 2x*Pdf-GAL4>CRYΔ*, *Pdf-GAL4>CRYΔ* and *Pdf-GAL4>* flies. Increasing CRYΔ expression via 2x*Pdf-GAL4* resulted in a reduced proportion of single rhythm flies, as expected (one-tailed Fisher's exact test, P=0.002). The proportions of single rhythm flies were not different between *Pdf-GAL4>CRYΔ* flies and *Pdf-GAL4>* controls (one-tailed Fisher's exact test, P=0.53).

Genotypes: *w, UAS-cryΔ14.6; Pdf-GAL4/Pdf-GAL4; +/+* (N=34), *w, UAS-cryΔ14.6; Pdf-GAL4/+; +/+* (N=47*), *yw; Pdf-GAL4/+; +/+* (N=61*). * Data are the same as in Table S1.

Table S1. Locomotor activity period (τ) after overexpression of CRYΔ in combinations of PDF+/- and CRY+/- neurons. Related to Figure 1

Genotype	Over-expressing	N (n)	τ ± S.E.M.	SR(%)	CR(%)	AR(%)	Neurons
w, UAS-cryΔ14.6; tim-GAL4/+; +/+	CRYΔ	48 (3)	24.90 ± 0.07[a1]	71	25	4	PDF$^+$ CRY$^+$, PDF$^-$ CRY‡,
w; tim-GAL4/+; UAS-cryΔ4.1/+	CRYΔ	38 (3)	25.75 ± 0.22	84	11	5	PDF$^-$ CRY*, PDF$^-$ CRY$^-$
w, UAS-HAcryΔ15.3; tim-GAL4/+; +/+	CRYΔ	27 (2)	25.44 ± 0.16	89	11	0	
w, UAS-cryΔ14.6; Pdf-GAL4/+; +/+	CRYΔ	47 (3)	24.64 ± 0.06[a2]	87	11	2	PDF$^+$CRY$^+$
w; Pdf-GAL4/+;UAS-cryΔ4.1/+	CRYΔ	32 (2)	25.13 ± 0.12	94	3	3	
w, UAS-HAcryΔ15.3; Pdf-GAL4/+; +/+	CRYΔ	18 (1)	26.28 ± 0.20	72	22	6	
w, UAS-cryΔ14.6; tim-GAL4/+; cry-GAL80$_{2e3m}$/+	CRYΔ	54 (3)	22.50 ± 0.06[a3]	79	15	6	PDF$^-$ CRY*, PDF$^-$ CRY$^-$
w; tim-GAL4/+; cry-GAL80$_{2e3m}$/UAS-cryΔ4.1	CRYΔ	48 (4)	22.77 ± 0.08	77	6	17	
w,UAS-HAcryΔ15.3; tim-GAL4/+;cry-GAL80$_{2e3m}$/+	CRYΔ	38 (2)	22.63 ± 0.06	68	11	21	
w, UAS-cryΔ14.6; Pdf-GAL80$_{96a}$/+; cry$_{13}$-GAL4/+	CRYΔ	33 (2)	24.08 ± 0.08[a4]	88	9	3	PDF$^-$ CRY‡, PDF$^-$ CRY*
w, UAS-cryΔ14.6; tim-GAL4/Pdf-GAL80$_{96a}$; +/+	CRYΔ	71 (5)	23.51 ± 0.09[a5]	69	28	3	PDF$^-$ CRY‡, PDF$^-$ CRY*, PDF$^-$ CRY$^-$
w, UAS-cryΔ14.6; tim-GAL4, Pdf-GAL80/+; Pdf-GAL80/+	CRYΔ	27 (2)	23.66 ± 0.08[a5]	78	22	0	
w, UAS-cryΔ14.6; +/+; cry$_{13}$-GAL4/+	CRYΔ	67 (4)	24.67 ± 0.06[a6]	90	10	0	PDF$^+$ CRY$^+$, PDF$^-$ CRY‡, PDF$^-$ CRY*
w, UAS-cryΔ14.6; +/+; +/+		46 (4)	23.78 ± 0.06[a7]	76	15	9	Controls
w; +/+; UAS-cryΔ4.1/+		47 (4)	23.81 ± 0.09	77	17	6	
w, UAS-HAcryΔ15.3;+/+; +/+		15 (1)	24.10 ± 0.12	100	0	0	
w; tim-GAL4/+; +/+		70 (7)	23.79 ± 0.05[a8]	82	11	7	
w; Pdf-GAL4/+; +/+		61 (5)	23.57 ± 0.05[a9]	89	3	8	

w; +/+; cry_{13}-GAL4/+	43 (3)	23.76 ± 0.05[a10]	86	12	2
w, UAS-cryΔ14.6; Pdf-$GAL80_{96a}$/+; +/+	24 (3)	23.93 ± 0.07[a11]	87	13	0
yw; tim-GAL4, Pdf-GAL80/+; Pdf-GAL80/+	38 (3)	23.84 ± 0.07[a12]	84	16	0
w; tim-GAL4/+; cry-$GAL80_{2e3m}$/+	98 (8)	23.50 ± 0.05[a13]	75	10	15

The period of locomotor activity was determined by Fourier (spectral) analysis [S4]. Only male flies were studied. **N**= total number of flies examined; n = number of independent replicates. **τ ± S.E.M.**, average period of locomotor activity ± standard error of the mean of flies showing a single period. **SR(%)**, percentage of flies showing a single period. **CR(%)**, percentage of flies showing complex rhythms. **AR(%)**, percentage of arrhythmic flies.

14.6 (X chromosome), 4.1 (3rd Chromosome) and 15.3 (X Chromosome) indicate three independent *UAS-cryΔ* insertions. *HAcryΔ15.3* carries at its 5' the sequence for production of the HA epitope tag.

ANOVA a1-a13, $F_{12,574}$ = 94.92, P<<0.01; Bonferroni post-hoc:

- PDF^{+} CRY^{+}, PDF^{-} CRY‡, PDF^{-} CRY*, PDF^{-} CRY^{-}>CRYΔ single rhythm flies had ~1h longer activity rhythms than controls, a1 ***vs*** a8, P<<0.001; a1 ***vs*** a7, P<<0.001
- PDF^{+} CRY^{+}>CRYΔ single rhythm flies had ~1h longer activity rhythms than controls, a2 ***vs*** a9, P<<0.001; a2 ***vs*** a7, P<<0.001
- PDF^{+} CRY^{+}, PDF^{-} CRY‡, PDF^{-} CRY*> CRYΔ single rhythm flies had ~1h longer activity rhythms than controls, a6 ***vs*** a10, P<<0.001; a6 ***vs*** a7, P<<0.001
- PDF^{+} CRY^{+}, PDF^{-} CRY‡, PDF^{-} CRY*, PDF^{-} CRY^{-}>CRYΔ were not different from PDF^{+} CRY^{+}>CRYΔ and PDF^{+} CRY^{+}, PDF^{-} CRY‡, PDF^{-} CRY*> CRYΔ single rhythm flies, a1 ***vs*** a2, P=0.63; a1 ***vs*** a6, P=0.82; a2 ***vs*** a6, P=1.00
- PDF^{-} CRY*, PDF^{-} CRY^{-}>CRYΔ single rhythm flies had ~1h shorter activity rhythms than controls, a3 ***vs*** a7, P<<0.001; a3 ***vs*** a13, P<<0.001
- PDF^{-} CRY‡, PDF^{-} CRY*, PDF^{-} CRY^{-}>CRYΔ single period flies were not different from controls, a5 ***vs*** a7, P=0.80; a5 ***vs*** a11, P=0.04; a5 ***vs*** a12, P=0.12
- PDF^{-} CRY‡, PDF^{-} CRY*>CRYΔ single period flies were not different from controls, a4 ***vs*** a10, P=0.14; a4 ***vs*** a11, P=1.00.

Table S2. Locomotor activity rhythms after manipulation of clock neurons. Related to Figure 3.

Genotype	Over-expressing	N (n)	τ ± S.E.M.	SR(%)	CR(%)	AR(%)	Neurons
yw; tim-GAL4/UAS-NaChBac4;+/+	NaChBac4	14 (1)		0	7	93	PDF^{+} CRY^{+}, PDF^{-} CRY‡,
yw; tim-GAL4/+;+/ UAS-NaChBac2	NaChBac2	16 (1)	24.04 ± 0.17	44	0	56	PDF^{-} CRY*, PDF^{-} CRY^{-}
w; tim-GAL4/ UAS-ClkΔ1;+/+	CLKΔ1	26 (2)		0	4	96	
yw; Pdf-GAL4/UAS-NaChBac4;+/+	NaChBac4	39 (2)	25.40 ± 0.18[a]	36	54	10	PDF^{+}CRY^{+}
yw; Pdf-GAL4/+;+/ UAS-NaChBac2	NaChBac2	26 (2)	26.28 ± 0.41[b]	42	50	8	
yw; Pdf-GAL4/+; +/ UAS-Kir2.1	KIR2.1	42 (2)	22.94 ± 0.10[i]	43	12	45	
yw; Pdf-GAL4/UAS-Kir2.1; +/+	KIR2.1	43 (3)	23.34 ± 0.08[j]	53	14	33	
yw, UAS-hid, UAS-rpr; Pdf-GAL4/+; +/+	HID, RPR	35 (1)	22.8 ± 0.12[l]	54	23	23	
yw; Pdf-GAL4/ UAS-ClkΔ1;+/+	CLKΔ1	35 (2)	23.15 ± 0.16[n]	48	29	23	
yw; Pdf-GAL4/ UAS-ClkΔ2;+/+	CLKΔ2	32 (3)	22.52 ± 0.24[o]	40	13	47	
w; tim-GAL/UAS-NaChBac4;cry-GAL80$_{2e3m}$*/+*	NaChBac4	24 (2)	23.06 ± 0.49[c]	13	8	79	PDF^{-} CRY*, PDF^{-} CRY^{-}
w; tim-GAL4/+;cry-GAL80$_{2e3m}$*/ UAS-NaChBac2*	NaChBac2	39 (2)	23.72 ± 0.16[d]	25	3	72	
yw, UAS-hid, UAS-rpr; tim-GAL4/+; cry-GAL80$_{2e3m}$ */+*	HID, RPR	18 (3)	24.12 ± 0.39[m]	28	11	61	
w; tim-GAL4/ UAS-ClkΔ1;cry-GAL80$_{2e3m}$*/+*	CLKΔ1	34 (3)	32.00	3	3	94	
w; tim-GAL4/ UAS-ClkΔ2;cry-GAL80$_{2e3m}$*/+*	CLKΔ2	16 (3)		0	0	100	
yw; tim-GAL4, Pdf-GAL80/UAS-NaChBac4; Pdf-GAL80/+	NaChBac4	52 (2)	24.36 ± 0.07[e]	81	17	2	PDF^{-} CRY‡, PDF^{-} CRY*, PDF^{-} CRY^{-}
yw; tim-GAL4, Pdf-GAL80/+; Pdf-GAL80/UAS-NaChBac2	NaChBac2	34 (2)	24.83 ± 0.07[f]	67	24	9	
yw; +/UAS-NaChBac4; cry$_{13}$*-GAL4/+*	NaChBac4	46 (2)	23.57 ± 0.37[g]	26	41	33	PDF^{+} CRY^{+}, PDF^{-} CRY‡, PDF^{-} CRY*
yw; +/+; cry$_{13}$*-GAL4/ UAS-NaChBac2*	NaChBac2	33 (2)	23.76 ± 0.35[h]	31	45	24	
w; +/ UAS-ClkΔ1; cry$_{13}$*-GAL4/+*	CLKΔ1	22 (2)	23.47 ± 0.13[p]	72	14	14	

w; +/ UAS-ClkΔ2; cry13-GAL4/+	CLKΔ2	35 (2)	23.43 ± 0.04[q]	94	6	0	
w; +/ +; cry13-GAL4/ UAS-Kir2.1	KIR2.1	32 (2)	23.60 ± 0.11[k]	78	13	9	
w; Pdf-GAL80_{96a} / UAS-ClkΔ1; cry13-GAL4/+	CLKΔ1	13 (2)	23.83 ± 0.07[r]	92	8	0	PDF$^-$ CRY‡, PDF$^-$ CRY*
yw; tim-GAL4/+; +/+		70 (7)	23.79 ± 0.05	72	11	7	Controls
yw; Pdf-GAL4/+; +/+		61 (5)	23.57 ± 0.05[a,b,i,j, l,n,o]	89	3	8	
yw; +/+; cry13-GAL4/+		43 (2)	23.76 ± 0.05[g,h,k,p,q,r]	86	12	2	
w; tim-GAL4/+; cry-GAL80_{2e3m}/+		98 (8)	23.50 ± 0.05[c,d,m]	75	10	15	
yw; tim-GAL4, Pdf-GAL80/+; Pdf-GAL80/+		38 (3)	23.84 ± 0.07[e,f]	84	16	0	
w; +/ UAS-NaChBac4;+/+		22 (2)	24.11 ± 0.09 [a,c,e,g]	86	9	5	
w; +/+;+/ UAS-NaChBac2		24 (2)	24.08 ± 0.05 [b,d,f,h]	87	13	0	
w; +/+; +/ UAS-Kir2.1		12 (1)	23.22± 0.09[i,k]	92	0	8	
w; +/UAS-Kir2.1;+/+		22 (1)	23.67± 0.07[j]	91	9	0	
yw, *UAS-hid*, *UAS-rpr*; +/+; +/+		30 (1)	24.27 ± 0.15[l,m]	93	7	0	
w; +/UAS-ClkΔ1;+/+		35 (3)	23.64 ± 0.06[n,p,r]	94	3	3	
w; +/UAS-ClkΔ2;+/+		24 (4)	23.53 ± 0.06[o,q]	92	4	4	
w, UAS-cryΔ14.6; Pdf-GAL80_{96a}/+; +/+		24 (2)	23.93 ± 0.07[r]	87	13	0	

The period of locomotor activity was determined by Fourier (spectral) analysis [S4]. Only male flies were studied. **N** = total number of flies examined; n = number of independent replicates. **τ ± S.E.M.**, average period of locomotor activity ± standard error of the mean of flies showing a single period. **SR(%)**, percentage of flies showing a single period. **CR(%)**, percentage of flies showing complex rhythms. **AR(%)**, percentage of arrhythmic flies.

[a,b] PDF$^+$CRY$^+$>NaChBac single period flies had > 1h longer activity rhythms than controls (overall [a]ANOVA, $F_{2,84}$ = 98.42, P<<0.01; Bonferroni post-hoc *yw; Pdf-GAL4/UAS-NaChBac4;+/+* **vs** *yw; Pdf-GAL4/+; +/+*, P<<0.01, **vs** *w; +/ UAS-NaChBac4;+/+* , P<<0.01. Overall [b]ANOVA, $F_{2,83}$ = 103.24, P<<0.01; Bonferroni post-hoc: *yw; Pdf-GAL4/+;+/ UAS-NaChBac2* **vs** *yw; Pdf-GAL4/+; +/+*, P<<0.01, **vs** *w; +/+;+/UAS-NaChBac2* , P<<0.01).

[c,d] PDF^{-} CRY*, PDF^{-}CRY^{-}>NaChBac single period flies had a tendency for shorter activity rhythms but neither line reached significance over both controls (overall [c]ANOVA, $F_{2,92}$ = 17.92, P<<0.01; but Bonferroni post-hoc: *w; tim-GAL/UAS-NaChBac4;cry-GAL80*$_{2e3m}$*/+* **vs** *w; tim-GAL4/+; cry-GAL80*$_{2e3m}$/+, P=0.25, **vs** *w; +/ UAS-NaChBac4;+/+*, P<<0.01. Overall [d]ANOVA, $F_{2,99}$ = 15.89, P<<0.01; but Bonferroni post-hoc: *w; tim-GAL4/+;cry-GAL80*$_{2e3m}$*/ UAS-NaChBac2* **vs** *w; tim-GAL4/+; cry-GAL80*$_{2e3m}$/+, P=0.38, **vs** *w; +/+;+/UAS-NaChBac2* , P=0.06).

[e,f] PDF^{-} CRY‡, PDF^{-} CRY*, PDF^{-}CRY^{-}>NaChBac single period flies had 0.5-1 h longer activity rhythms than controls. Differences were suggestive for one line and significant for the other (overall [e]ANOVA, $F_{2,90}$ = 13.40, P<<0.01; but Bonferroni post-hoc: *yw; tim-GAL4, Pdf-GAL80/UAS-NaChBac4; Pdf-GAL80/+* **vs** *yw; tim-GAL4, Pdf-GAL80/+; Pdf-GAL80/+*, P<<0.01, **vs** *w; +/ UAS-NaChBac4;+/+*, P=0.10. Overall [f]ANOVA, $F_{2,73}$ = 55.34, P<<0.01; Bonferroni post-hoc: *yw; tim-GAL4, Pdf-GAL80/+; Pdf-GAL80/UAS-NaChBac2* **vs** *yw; tim-GAL4, Pdf-GAL80/+; Pdf-GAL80/+*, P<<0.01, **vs** *w; +/+;+/UAS-NaChBac2* , P<<0.01).

[g,h] PDF^{+}CRY^{+}, PDF^{-} CRY‡, PDF^{-} CRY*>NaChBac single period flies were not significantly different from controls ([g]ANOVA, $F_{2,65}$ = 3.36, P=0.41; [h]ANOVA, $F_{2,65}$ = 3.33, P=0.42).

[i,j] PDF^{+}CRY^{+}>Kir2.1 single period flies had slightly shorter activity rhythms than controls. Differences were suggestive for one line and significant for the other (overall [i]ANOVA, $F_{2,80}$ = 20.87, P<<0.01; but Bonferroni post-hoc *yw; Pdf-GAL4/+; +/ UAS-Kir2.1* **vs** *yw; Pdf-GAL4/+; +/+*, P<<0.01, **vs** *w; +/+; +/ UAS-Kir2.1* , P=0.15. Overall [j]ANOVA, $F_{2,94}$ = 4.82, P=0.01; Bonferroni post-hoc: *yw; Pdf-GAL4/UAS-Kir2.1; +/+* **vs** *yw; Pdf-GAL4/+; +/+*, P=0.04, **vs** *w; +/UAS-Kir2.1;+/+* , P=0.01).

[k] PDF^{+}CRY^{+}, PDF^{-} CRY‡, PDF^{-} CRY*>Kir2.1 single period had intermediate periods compared to controls (overall ANOVA, $F_{2,70}$ = 7.68, P<<0.01; but Bonferroni post-hoc: *w; +/ +; cry*$_{13}$*-GAL4/ UAS-Kir2.1* **vs** *yw; +/+; cry*$_{13}$*-GAL4/+*, P=0.43, **vs** *w; +/+; +/ UAS-Kir2.1*, P=0.03).

[l] PDF^{+}CRY^{+}>HID, RPR single period flies had shorter activity rhythms than controls (overall ANOVA, $F_{2,98}$ = 43.37, P<<0.01; Bonferroni post-hoc *yw, UAS-hid, UAS-rpr; Pdf-GAL4/+; +/+* ***vs*** *yw; Pdf-GAL4/+; +/+*, P<<0.01, ***vs*** *yw*, *UAS-hid*, *UAS-rpr*; *+/+; +/+*, P<<0.01).

[m] PDF^{-} CRY*, PDF^{-}CRY^{-}> HID, RPR single period flies showed intermediate periods compared to controls (overall ANOVA, $F_{2,103}$ = 21.35, P<<0.01; but Bonferroni post-hoc: *yw, UAS-hid, UAS-rpr; tim-GAL4/+; cry-GAL80*$_{2e3m}$ */+* ***vs*** *w; tim-GAL4/+; cry-GAL80*$_{2e3m}$*/+*, P=0.05, ***vs*** *yw*, *UAS-hid*, *UAS-rpr*; *+/+; +/+*, P=1.00).

[n,o] PDF^{+}CRY^{+}>CLKΔ single period flies had shorter activity rhythms than controls (overall [n]ANOVA, $F_{2,102}$ = 9.36, P<<0.01; Bonferroni post-hoc *yw; Pdf-GAL4/ UAS-ClkΔ1;+/+* ***vs*** *yw; Pdf-GAL4/+; +/+*, P<<0.01, ***vs*** *w; +/UAS-ClkΔ1;+/+*, P<<0.01. Overall [o]ANOVA, $F_{2,86}$ = 31.10, P<<0.01; Bonferroni post-hoc *yw; Pdf-GAL4/ UAS-ClkΔ2;+/+* ***vs*** *yw; Pdf-GAL4/+; +/+*, P<<0.01, ***vs*** *w; +/UAS-ClkΔ2;+/+*, P<<0.01).

[p,q] PDF^{+}CRY^{+}, PDF^{-} CRY‡, PDF^{-} CRY*> CLKΔ single period flies were not significantly different from controls (overall [p]ANOVA, $F_{2,83}$ = 3.50, P=0.04; but Bonferroni post-hoc: *w; +/ UAS-ClkΔ1; cry*$_{13}$*-GAL4/+* ***vs*** *yw; +/+; cry*$_{13}$*-GAL4/+*, P=0.03, ***vs*** *w; +/UAS-ClkΔ1;+/+*, P=0.40. Overall [q]ANOVA, $F_{2,89}$ = 12.91, P<<0.01; but Bonferroni post-hoc: *w; +/ UAS-ClkΔ2; cry*$_{13}$*-GAL4/+* ***vs*** *yw; +/+; cry*$_{13}$*-GAL4/+*, P<<0.01, ***vs*** *w; +/UAS-ClkΔ2;+/+*, P=0.54).

[r] PDF^{-} CRY‡, PDF^{-} CRY*> CLKΔ single period flies were not significantly different from controls (overall ANOVA, $F_{3,99}$ = 3.97, P=0.01; but Bonferroni post-hoc: *w; Pdf-GAL80*$_{96a}$ */ UAS-ClkΔ1; cry*$_{13}$*-GAL4/+* ***vs*** *w; yw; +/+; cry*$_{13}$*-GAL4/+*, P=1.00, ***vs*** *w; +/UAS-ClkΔ1;+/+*, P=0.40, ***vs*** *w, UAS-cryΔ14.6; Pdf-GAL80*$_{96a}$*/+; +/+*, P=1.00)

Table S3. Locomotor activity rhythms after overexpression of CRYΔ in DN1p neurons. Related to Figure 4.

Genotype	Over-expressing	N (n)	τ ± S.E.M.	SR(%)	CR(%)	AR(%)	Neurons
w, UAS-cryΔ14.6; +/+; CLK4.1M-GAL4/ +	CRYΔ	114 (2)	23.64 ± 0.03	94	5	1	several DN1p
w, UAS-cryΔ14.6; +/+; CLK4.5F-GAL4/ +	CRYΔ	50 (2)	23.43 ± 0.04	94	4	2	several DN1p
w, UAS-cryΔ14.6; +/+; +/+		46 (4)	23.78 ± 0.06	76	15	9	Controls
w; +/+; CLK4.5F-GAL4/+		32 (1)	23.50 ± 0.04	97	0	3	Controls

The period of locomotor activity was determined by Fourier (spectral) analysis [S4]. Only male flies were studied. **N** = total number of flies examined; n = number of independent replicates. **τ ± S.E.M.**, average period of locomotor activity ± standard error of the mean of flies showing a single period. **SR(%)**, percentage of flies showing a single period. **CR(%)**, percentage of flies showing complex rhythms. **AR(%)**, percentage of arrhythmic flies.

ANOVA, Genotypes, $F_{3,216}$=10.979, P<<0.001. Bonferroni post-hoc comparisons: the genotype *w, UAS-cryΔ14.6; +/+; CLK4.1M-GAL4/* + was not significantly different from the parental control *w, UAS-cryΔ14.6; +/+; +/+* (P=0.088). The genotype *w, UAS-cryΔ14.6; +/+; CLK4.5F-GAL4/* + was not significantly different from the parental control *w; +/+; CLK4.5F-GAL4/+* (P=1). Therefore, driving CRYΔ in several DN1p does not result in a shorter period.

Table S4. Locomotor activity rhythms after developmental stage-specific manipulation of clock neurons. Related to Figure 5.

Genotype	Condition	Over-expressing	N (n)	τ ± S.E.M.	SR(%)	CR(%)	AR(%)	Neurons
w, UAS-cryΔ14.6; UAS-CD8GFP/+; Pdf-GS/+	DO		78 (2)	23.69 ± 0.05^{a}	97	0	3	$PDF^{+}CRY^{+}$
w, UAS-cryΔ14.6; UAS-CD8GFP/+; Pdf-GS/+	DA	$CRY\Delta_{A}$	64 (2)	24.01 ± 0.07^{a}	92	5	3	
w, UAS-cryΔ14.6; UAS-CD8GFP/+; Pdf-GS/+	DC	$CRY\Delta_{Ch}$	26 (1)	24.22 ± 0.10^{a}	100	0	0	
yw; Pdf-GAL4/+; +/UAS-dTrpA1	18 ºC	$dTRPA1_{C}$	20 (1)	23.93 ± 0.07^{b}	85	0	15	
yw; Pdf-GAL4/+; +/UAS-dTrpA1	28 ºC	$dTRPA1_{O}$	24 (1)	24.40 ± 0.18^{b}	62	21	17	
yw; tim-GAL4/+; cry-GAL80$_{2e3m}$*/ UAS-dTrpA1*	18 ºC	$dTRPA1_{C}$	14 (1)	24.11 ± 0.51^{c}	72	7	21	$PDF^{-}CRY^{*}$, $PDF^{-}CRY^{-}$
yw; tim-GAL4/+; cry-GAL80$_{2e3m}$*/ UAS-dTrpA1*	28 ºC	$dTRPA1_{O}$	16 (1)	22.66 ± 0.26^{c}	75	19	6	
w; UAS-CD8GFP/+; Pdf-GS/+	DO		81 (3)	23.89 ± 0.05^{a}	91	4	5	Controls
w; UAS-CD8GFP/+; Pdf-GS/+	DA		65 (2)	24.08 ± 0.05^{a}	91	6	3	
w; UAS-CD8GFP/+; Pdf-GS/+	DC		33 (1)	24.01 ± 0.08^{a}	91	3	6	
w, UAS-cryΔ14.6; +/+; +/+	DO		93 (2)	23.89 ± 0.07^{a}	70	4	26	
w, UAS-cryΔ14.6; +/+; +/+	DA		80 (2)	24.01 ± 0.16^{a}	70	5	25	
w, UAS-cryΔ14.6; +/+; +/+	DC		58 (1)	23.86 ± 0.10^{a}	71	10	19	
yw; Pdf-GAL4/+; +/+	18 ºC		22 (1)	23.88 ± 0.07^{b}	100	0	0	
yw; Pdf-GAL4/+; +/+	28 ºC		28 (1)	24.06 ± 0.06^{b}	100	0	0	
w; tim-GAL4/+; cry-GAL80$_{2e3m}$*/+*	18 ºC		24 (1)	23.88 ± 0.31^{c}	38	0	62	
w; tim-GAL4/+; cry-GAL80$_{2e3m}$*/+*	28 ºC		32 (1)	23.76 ± 0.09^{c}	69	13	18	
w; +/+; +/ UAS-dTrpA1	18 ºC		11 (1)	22.94± 0.18$^{b, c}$	91	0	9	
w; +/+; +/ UAS-dTrpA1	28 ºC		19 (1)	23.09± 0.11$^{b, c}$	95	0	5	

The period of locomotor activity was determined by Fourier (spectral) analysis [S4]. Only male flies were studied. **N** = total number of flies examined; n = number of independent replicates. **τ ± S.E.M.**, average period of locomotor activity ± standard error of the mean of flies showing a single period. **SR(%)**, percentage of flies showing a single period. **CR(%)**, percentage of flies showing complex rhythms. **AR(%)**, percentage of arrhythmic flies. Experiments carried out with the

temperature activated cation channel dTRPA1 were conducted at 18 ºC and 28 ºC to maintain the channel in the close ($dTRPA1_C$) or open ($dTRPA1_O$) conformation, respectively. All other experiments were conducted at 25 ºC. The *geneswitch* (*GS*) system requires the drug RU486 to be activated into a functional GAL4. Experiments using *GS* were conducted in the absence of drug (DO, drug omitted), exposing the adults only to the drug (DA, drug acute treatment), exposing individuals to the drug since the earliest developmental stages (DC, drug chronic treatment).

[a] ANOVA, Genotype, $F_{2,477}$=0.554, P=0.575, Treatment, $F_{2,477}$=7.218, P=0.001, Genotype*Treatment, $F_{4,477}$=2.529, P=0.04. Bonferroni post-hoc comparisons, DO *vs*. DA, P=0.001, DO *vs*. DC. P=0.037, DA *vs*. DC, P=1.00.

[b] ANOVA, Genotype, $F_{2,104}$=57.694, P<0.001, Temperature, $F_{1,104}$=9.349, P=0.003, Genotype*Temperature, $F_{2,104}$=1.452, P=0.239.

[c] ANOVA, Genotype, $F_{2,75}$=6.219, P=0.003, Temperature, $F_{1,75}$=6.200, P=0.015, Genotype*Temperature, $F_{2,75}$=6.322, P=0.003.

Table S5. Locomotor activity rhythms after overexpression of SGG and CRY. Related to Figure 6.

**Genotype**	**Over-expressing**	**N (n)**	**τ ± S.E.M.**	**SR(%)**	**CR(%)**	**AR(%)**	**Neurons**
w, UAS-sgg; tim-GAL4/+; cry-GAL80$_{2e3m}$/ +	SGG	78 (5)	23.57 ± 0.08	65	35	0	$PDF^{-}CRY^{*}$, $PDF^{-}CRY^{-}$
w; tim-GAL4/+; cry-GAL80$_{2e3m}$/ UAS-HAcry16.1	CRY	29 (2)	23.72 ± 0.18	72	21	7	
w, UAS-sgg; tim-GAL4/+; cry-GAL80$_{2e3m}$/ UAS-HAcry16.1	SGG, CRY	46 (3)	22.79 ± 0.13	74	13	13	
w; tim-GAL4/+; cry-GAL80$_{2e3m}$/+		98 (8)	23.50 ± 0.05	75	10	15	Controls
w, UAS-sgg; +/+; +/+		47 (4)	23.75 ± 0.05	96	4	0	
w; +/+; +/ UAS-HAcry16.1		16 (1)	23.90 ± 0.06	88	12	0	

The period of locomotor activity was determined by Fourier (spectral) analysis [S4]. Only male flies were studied. **N** = total number of flies examined; n = number of independent replicates. **τ ± S.E.M.**, average period of locomotor activity ± standard error of the mean of flies showing a single period. **SR(%)**, percentage of flies showing a single period. **CR(%)**, percentage of flies showing complex rhythms. **AR(%)**, percentage of arrhythmic flies. *HAcry16.1* carries at its 5' the sequence for production of the HA epitope tag.

ANOVA, Genotypes, $F_{5,232}$=15.909, P<<0.001. Bonferroni post-hoc comparisons, the genotype *w, UAS-sgg; tim-GAL4/+; cry-GAL80$_{2e3m}$/ UAS-hacry16.1/+* resulted significantly different in all pairwise comparisons with the other genotypes (P<<0.001). The comparisons between all other genotypes were not significant.

Supplemental Experimental Procedures

Fly strains and maintenance.

Flies were raised at 25 °C on standard yeast-corn-sucrose-agar medium under LD 12:12. All strains employed have been previously described, as follows. Reporter (UAS-) lines, *CRY*Δ and *CRY* [5], *NachBac* [19] (obtained from the Bloomington Stock centre), *Kir2*.1 [24] (obtained from K Moffat), *CLK*Δ [28], *HID,RPR* [26], *SGG* (constitutively active form) [S5], *RFP*^{NLS} (aka *RedStinger*), *GFP* and *TRPA1* (Bloomington Stock centre). Driver lines were as described in [14], with the exception of *cry*$_{39}$-*GAL4* reported in [S6], *tim-GAL4, Pdf-GAL80; Pdf-GAL80* reported in [S7], *Pdf-GeneSwitch* reported in [30] and *Pdf-GAVPO* (this publication, see below).

Behavioural analyses and statistics

Locomotor activity was recorded (males only) with DAM2 Trikinetics monitors for 3-5 days in LD 12:12 and 7-12 days in DD, at constant 25 °C. Activity periods were determined using the CLEAN package [S4]. Briefly, flies were considered rhythmic in the circadian range if showing a (spectral analysis) harmonic component (which also defines the period of the fly) in the interval 12 h< > 30 h, such that it exceeded the 99% confidence limit calculated by a Monte Carlo simulation (single rhythm flies, SR). Flies were considered showing complex rhythms (CR) when two or more significant (above the 99% confidence limit) harmonic components in the circadian range where present in the same individual. Arrhythmic (AR) animals did not show significant harmonics in the circadian range. When required we calculated the Power of a rhythm as a measure of its robustness. Based on the empirical observation that the more robust the rhythm the higher the peak value (P) of the harmonic component compared to the 99% confidence limit (CL_{99}), we defined Power as $Pw = [(P-CL_{99})/CL_{99} * 100]$.

Statistical analyses were carried out using PASW Statistics 18 (Release 18.0.2).

Immunohistochemistry

For each experiment, flies were processed in parallel. Flies were first fixed at room temperature (as for the whole procedure except incubation with primary antibodies) on a rotating wheel for 2 h with 4% formaldehyde, 0.1% Triton-X in PBS. Then they were washed for 15 min in PBS, and finally brains were dissected in PBS. Brains were permeabilised 3 x 20 min with PBS, 1% Triton-X. Blocking was performed in PBS, 0.5% Triton-X, 10% goat serum for 1 h. Primary antibodies were diluted in fresh PBS, 0.5% Triton-X, 10% goat serum and incubated for 72 h at 4 °C. Unbound antibodies were washed 3 x 20 min with PBS, 0.5% Triton-X. Secondary fluorescent antibodies were diluted in PBS, 0.5% Triton-X and incubated (in darkness) for 3 h. After washes (3x 20 min, PBS, 0.5% Triton-X) brains were rinsed in distilled water and mounted in 20% PBS (pH = 8.5), 80% Glycerol + 3% n-propylgallate (Sigma). Primary antibodies were as follows. Mouse anti-PDF (1:50, DSHB), rabbit anti-PDP1ε (1:5000, gift of Justin Blau), rabbit anti-GFP (1:1000, Invitrogen), mouse anti-GFP (1:2000, BAbCo), rabbit anti-CRY (1:500, see below). Fluorescent secondary antibodies were as follows. Goat anti-rabbit IgG-Cy3 (1:200, Jackson), goat anti-mouse IgG-Cy2 (1:200, Jackson), goat anti-rabbit IgG-AlexaFluor647 (Jackson, 1:200), goat anti-rabbit IgG-Biotin (1: 600, Jackson), Streptavidin Dylight-649 (1:600, Jackson).

Optical sections were imaged on a Leica TCS SP5 confocal microscope (Figure 4) or with an Olympus FV1000 (Figure S1) confocal microscope.

Images were analysed with ImageJ software to quantify the mean pixel intensity (mpi) of cytoplasm, nucleus and background by an experimenter that was blind to the identity of the sample. Staining Index (SI) was calculated with the following formula:

$[(Cell_{mpi} - background_{mpi})/background_{mpi}]$x(number of cells observed/maximum number of cells).

For each hemisphere we then calculated an average for each cell type. These were the values used for statistical analyses. For the different types of neurons we used the following as the maximum number of cells, s-LNvs=4; l-LNvs=4; LNds=6; DN2=2; DN1s=16; DN3s=40. All cells were quantified individually with the exception of the DN3s that were quantified as a group.

Production of anti-CRY antibody.

We amplified the coding sequence of *cry* as three overlapping fragments:

f1 (from 1 to 600 bp. Primers: F, 5'-GGCAT**ATG**GCCACGCGAGGGGCGAATG-3';

R, 5'-GGGGATCCTTACTCGAACAACTTAAGACTTCGGCAG-3'),

f2 (540-1296 bp. Primers: F, 5'-CGCAT**ATG**GAAGACGCCACCTTTGTCGAGC-3';

R, 5'-GGGGATCCTTACAGCAGCCTTTCAAACGCCGAG-3') and

f3 (999-1626 bp. Primers: F, 5'-GGCAT**ATG**AATGACATCTGCCTGAGCATCCCG-3';

R, 5'-GGGGATCCTTAAACCACCACGTCGGCCAGCCAG-3').

All F primers carried a 5' *Nhe*I site and a start codon (in bold); all R primers carried a 5' *Xho*I site and a stop codon (underlined). After amplification the three products were cloned as *Nhe*I-*Xho*I fragments into pET14b (Merck Millipore) and were verified by Sanger sequencing. We expressed the three His6x-fusion proteins in *E. coli* BL21(DE3)pLysS strain (Merck Millipore) and we purified them by chromatography on Ni^+-resin as insoluble products. Denaturants were removed by dialysis and the proteins were resuspended in PBS at a final concentration of 0.4 mg/ml. The three fragments mixed together were injected in rabbits by a commercial service (Polypeptide Laboratories).

Cloning of Pdf-GAVPO

We amplified *GAVPO* [S3] by PCR (from the first ATG to the HSP70 terminator sequence) using PHUSION DNA polymerase (all enzymes were from New England Biolabs) and the following primers:

Geneart_GAVPO_F_{KOZAK}:

5’-TACCAAACGGTACCGCTAGCCAAAAC**ATG**AAGCTACTGTCTTCTATCGAA-3’

(underlined: KOZAK sequence, bold: first codon) and

Geneart_GAVPO_R:

5’-TCGATAAGCTTGTTTAAACTTACTTGTCATCATCGTCTTTGTAG-3’.

The product was cloned into *LexAP65* (Addgene), previously digested with *Nhe*I and *Pme*I, using the GeneArt® Seamless Cloning and Assembly kit (Life Technologies). To increase mRNA stability we replaced *Hsp70* with the *SV40* terminator sequence. We amplified the latter from *pJFRC19* [S8] with primers

*Pme*I_SV40_F: 5’-ACTGGTTTAAACTAAGGTAAATATAAAATTTTTAAGTGTAT-3’

and *Xba*I_SV40_R: 5’-CAGTTCTAGAAGATCGATCCAGACATGATAA-3’

and cloned it into *pCR®-Blunt* (Life Technologies). Then we isolated the *SV40* sequence by *Pme*I and *Xba*I digestion and we inserted it (by T4 ligation) in exchange to the *Hsp70* equally restricted. Finally, we introduced the *Pdf* promoter sequence upstream to the *GAVPO* coding region. We amplified the promoter sequence from genomic DNA with primers:

PDF_pr_F: 5’-GCCCGGGCTCCGTGGGTTTCATCCTTACCA-3’ and

Kpn_PDF_pr_R: 5’-GGTACCCAGGAGACTTGCGAATGAACGT-3’.

We cloned it into *pCR®-Blunt* and then we excised it by *Kpn*I and *Eco*53kI restriction. This fragment was ligated upstream of the GAVPO coding sequence into *Kpn*I and *Nae*I (compatible with *Eco*53kI) sites resulting in *Pdf-GAVPO*. The accuracy of the construct was

verified by Sanger sequencing. Transgenic flies were produced by a commercial service (Cambridge University) using PhiC31-recombinase. We chose the *attP40* site for integration.

Supplemental references

S1. Yasuyama, K., and Meinertzhagen, I.A. (2010). Synaptic connections of PDF-immunoreactive lateral neurons projecting to the dorsal protocerebrum of *Drosophila melanogaster*. J Comp Neurol. 518, 292-304.

S2. Choi, C., Cao, G., Tanenhaus, A.K., McCarthy, E.V., Jung, M., Schleyer, W., Shang, Y., Rosbash, M., Yin, J.C., and Nitabach MN. (2012). Autoreceptor control of peptide/neurotransmitter corelease from PDF neurons determines allocation of circadian activity in *Drosophila*. Cell Rep. 2, 332-344.

S3. Wang, X., Chen, X. and Yang, Y. (2012) Spatiotemporal control of gene expression by a light-switchable transgene system. Nature Methods 9, 266-269.

S4. Rosato, E., and Kyriacou, C.P. (2006). Analysis of locomotor activity rhythms in Drosophila. Nat Protoc 1, 559-68.

S5. Martinek, S., Inonog, S., Manoukian, A.S., and Young, M.W. (2001). A role for the segment polarity gene *shaggy*/*GSK*-3 in the *Drosophila* circadian clock. Cell 105, 769-779.

S6. Klarsfeld, A., Malpel, S., Michard-Vanhée, C., Picot, M., Chélot, E. and Rouyer F. (2004) Novel features of cryptochrome-mediated photoreception in the brain circadian clock of *Drosophila*. J. Neurosci. 24, 1468–1477

S7. Murad, A., Emery-Le, M., and Emery, P. (2007). A subset of dorsal neurons modulates circadian behavior and light responses in Drosophila. Neuron 53, 689-701.

S8. Pfeiffer, B.D., Ngo, T.T., Hibbard. K.L, Murphy, C., Jenett, A., Truman, J.W. and Rubin, (2010) Refinement of tools for targeted gene expression in Drosophila. Genetics 186, 735-755.
